# Biophysical assessments and blood profiling reveal physiological adaptations and environmental interactions of hilsa shad (*Tenualosa ilisha*)

**DOI:** 10.1371/journal.pone.0320628

**Published:** 2025-04-01

**Authors:** Saeed Anwar, Abdul Kader, Smrity Kona Debnath, Faria Jarin, Abu Saleh Mohammad Sayem, Md. Faruque Miah

**Affiliations:** 1 Department of Genetic Engineering and Biotechnology, School of Life Sciences, Shahjalal University of Science and Technology, Sylhet, Bangladesh; 2 Department of Food Engineering and Tea Technology, School of Applied Sciences and Technology, Shahjalal University of Science and Technology, Sylhet, Bangladesh; Sher-e-Kashmir University of Agricultural Sciences and Technology of Kashmir, INDIA

## Abstract

The hilsa shad (*Tenualosa ilisha*) is a migratory fish of great economic and cultural importance in Bangladesh. However, its physiological adaptations to diverse environmental conditions are not well understood. This study provides a comprehensive assessment of the biophysical, hematological, and biochemical characteristics of hilsa shad from riverine, estuarine, and marine environments in Bangladesh. We sampled 180 adult fish from nine sites, revealing significant habitat-specific variations in water quality, with marine sites showing the highest levels of total dissolved solids (TDS) and specific conductance. Hematological analysis showed that fish from riverine environments had higher hematocrit values, which are crucial for oxygen transport during migration. Post-spawning fish exhibited elevated hemoglobin, RBC counts, and hematocrit levels, reflecting adaptations to the metabolic demands of migration and spawning. WBC counts were higher in females, particularly in estuarine and marine environments, suggesting a stronger immune response to environmental stressors. Serum biochemical analysis showed significant variations in liver enzyme activity, albumin, and cortisol levels, with elevated cortisol in riverine fish during spawning season, indicating heightened stress. These findings enhance our understanding of hilsa shad’s physiological ecology, providing critical insights for conservation and management strategies amid environmental changes.

## Introduction

Hilsa shad (*Tenualosa ilisha*) is a critically important transboundary species that is found in the Bay of Bengal and migrates upstream to rivers in Bangladesh (86% share), India (8% share), Myanmar (4% share), Pakistan, and the Persian Gulf area for breeding and nursing of fries [1,2]. The annual catch of hilsa shad exceeds half a million tons, making it the largest single-species fishery in the region [3]. This species contributes over 40% of the total catch in Bangladesh and accounts for a significant portion of the country’s fish production, representing more than 1/8th of the total fish production and over 1% of the country’s gross domestic product [3]. The high economic value of hilsa shad, along with its taste, distinctive flavor, and culinary properties, makes it a highly sought-after species in the region, particularly in Bangladesh and India’s West Bengal [4]. The non-consumptive value of hilsa shad is also substantial, estimated at nearly 0.4 billion USD per year, making it an important part of many cultural occasions and festivals in Bengali culture [5]. However, due to over-exploitation and environmental changes, the natural production of this fish has been declining recently [6,7]. This highlights the urgent need for research and the development of captive breeding strategies to sustainably manage this valuable species.

Recent attempts at captive breeding and artificial feeding for hilsa have gained momentum. However, as hilsa shad is an anadromous species, rearing it in captivity has long been a significant challenge. Furthermore, there is a lack of knowledge on the genetic, biochemical, and physiological features of hilsa shad, which hinders the development of this fishery in captivity. Understanding the molecular and physiological mechanisms of this fish’s adaptation during migration for spawning and growth is crucial for effective larval hatching and rearing in captivity [8,9]. Advanced studies on hilsa shad have only recently been initiated [10–13]. The species’ genome and transcriptome has recently been catalogued, offering key insights into its biology and evolution [10–13]. However, there is currently no systematic information available on the general physiology, e.g., blood physiology, of this species.

Blood physiology is critical in the homeostatic regulation of pH, temperature, and other internal conditions. Blood indices are essential indicators of fish health and immune status and have been widely recognized as powerful tools for fish health assessments [14–21]. These indices are closely linked to the fish’s physiological responses to various biological and environmental factors [20–22]. Any physical or chemical changes in the fish’s habitat, such as water quality, oxygen, temperature, and salinity, can impact fish growth, development, and various body functions [23]. It is well-established that fish blood indices quickly reflect the physiological impacts of these environmental changes [15,16,23]. As such, knowledge of hematology plays a crucial role in analyzing the physiological responses of fish to environmental changes [24–26]. These studies are especially valuable for migrating fishes, e.g., hilsa shad, which showcase large- or small-scale movements between environmentally different habitats that fulfill competing needs that may occur within and between different life stages [27].

During large-scale migration, e.g., from marine to freshwater and vice versa, these fish face challenges in osmoregulation processes, which significantly influence ionic and hormonal homeostasis [28]. These influences in homeostasis ultimately impact the health status and breeding biology of these fish through several signaling and physiological response mechanisms [29]. Understanding how migrating fish, e.g., hilsa shad, adapt to different environments for growth and breeding offers crucial insights for effective management of natural stocks and the development of captive breeding strategies for this and other species.

Given the lack of existing data on the blood indices of hilsa shad, the objective of this study is to conduct a comprehensive examination of the blood indices profile and immunity parameters of hilsa shad specimens collected from various habitats, including marine, brackish water, and freshwater, in order to gain insights into the physiological changes that occur in response to varying environmental conditions.

## Methods

### Ethics statement

The research involving the hilsa shad (*Tenualosa ilisha*) was conducted in accordance with the ethical guidelines and regulations outlined by the Institutional Ethical Committee (IEC) of Shahjalal University of Science and Technology (SUST), Sylhet, Bangladesh (IEC/101(1)/02). Field collections were conducted in compliance with national and institutional guidelines. No specific permits were required for the collection of hilsa shad as it is a commercially harvested species and not classified as threatened under the IUCN Red List. The fieldwork and sample collection were conducted in coordination with the Bangladesh Fisheries Research Institute (BFRI) and the Department of Fisheries, Ministry of Fisheries and Livestock, Government of the People’s Republic of Bangladesh. In Chandpur, Barisal, and Bhola, a personnel from the Department of Fisheries accompanied our site visits.

### Study site and sample collection

We collected 180 live and healthy specimens of *Tenualosa ilisha* from 9 sites, traditionally known for hilsa capture, which included 3 riverine (Kirtankhola River/Barisal, 22°43’37“N 90°26’06”E; Upper Padma River/Rajshahi, 24°26’09”N 88°19’44”E; Meghna River/Chandpur, 23°29’20”N 90°34’58”E), 3 estuarine (Meghna River Estuary/Bhola, 22°36’03”N 90°49’33”E; Tentulia River Estuary/Lalmohon/Dashmina, 22°15’18”N 90°37’08”E), Pashur River/Khulna, 21°59’13”N 89°31’15”E), and 3 marine (Bay of Bengal/Maheshkhali, 21°28’44”N 91°50’06”E; Bay of Bengal/Kuakata, 21°45’36”N 90°06’25”E; Bay of Bengal/Mongla/Khulna, 21°40’44”N 89°30’28”E) sites/ecosystems in Bangladesh. We collected at least 20 individuals (≥10 males and ≥ 10 females) from each site and only considered adult fish (individuals greater than 23 cm in length) for this study. Blood samples were collected immediately on-site by caudal vein puncture. We obtained ~ 2 mL or as much as possible of blood from each fish using a sterile syringe and 22G needle. A subsample of 1 mL of blood was transferred to a heparinized tube for hematological analyses, while the rest was transferred to a clot activator tube on ice for serum separation. Additionally, blood smears were prepared immediately after blood collection. The weight and length of each fish were recorded as soon as possible.

### Biophysical assessments of the sample collection sites

Data on the physicochemical parameters of water quality were recorded using a digital multimeter from each sampling location (HQ 40D Multimeter). The parameters measured included air and water temperature, pH, total dissolved solids (TDS), total suspended solids (TSS), salinity, dissolved oxygen, specific conductance, and water-current velocity. The biochemical oxygen demand over a five-day period (BOD_5_) was assessed in the water samples during a 5-day incubation period at a temperature of 20°C. Additionally, nitrogenous compounds (NO_3_^-^, NO_2_^-^ and NH_3_^+^), phosphate compound (PO_4_^++)^, Chlorophyll-a concentration was measured using spectrophotometric techniques [30].

### Hematological analysis

Hemoglobin levels were determined using the cyanmethemoglobin method [31]. The total red blood cells, white blood cells, and platelet counts were assessed using an automated cell counter (HeCo Vet C, SEAC, Italy), in accordance with previously established protocols [32]. To estimate the percent hematocrit (%), we transferred ~ 50 µL of heparinized blood to a micro-hematocrit capillary and spun it at 8,000*g* for 6 minutes in a micro-centrifuge machine (REMI RM-12C BL, India). The resulting values were expressed as percentages [33]. Secondary hematological indices—mean corpuscular hemoglobin (MCH), mean corpuscular hemoglobin concentration (MCHC), and mean corpuscular volume (MCV)—were computed based on hemoglobin levels, red blood cell counts, and packed cell volume (PCV), following established methodologies [34].

### Serum biochemical analysis

The serum was extracted from the blood samples collected in clot-activator tubes through centrifugation at 4000g for a duration of 10 minutes. Following this process, the serum was transferred to a fresh 1.5 mL tube, securely placed on dry ice, and subsequently stored at − 20˚C for future analysis. Serum levels of glucose, cholesterol, urea, total protein, albumin, and globulin were measured utilizing a biochemistry analyzer (Vetscan VS2, Abaxis, USA).

### Statistical analysis

The data were analyzed using standard statistical procedures, which included the calculation of means and standard errors. To assess the differences in blood indices among individuals from different habitats, one-way ANOVA was employed, followed by Tukey’s post hoc analysis when significant effects were detected. Additionally, Student’s t-tests were utilized to compare specific groups. Correlation analyses were conducted to explore the relationships between variables in one of the experiments. Statistical significance was assessed at four α-levels: 0.05, 0.01, 0.001, and 0.0001 (*p* =  0.05, *p* =  0.01, *p* =  0.001, and *p* =  0.0001, respectively). All analyses were performed using GraphPad Prism 10.2.1 (GraphPad Software, USA) and Microsoft Excel (Microsoft Corporation, USA).

## Results

### Variability in physicochemical parameters across ecosystems reflects distinct environmental conditions

Table 1 summarizes the physicochemical parameters of water from the sampled sites (Table 1). Air and water temperatures ranged from 24.32°C to 32.87°C and 20.35°C to 30.9°C, respectively, with no significant differences among the sites (S1 Fig). Total dissolved solids (TDS) varied significantly across ecosystems, with riverine sites ranging from 39 to 235 mgL^–1^, estuarine sites from 592 to 1792 mgL^–1^, and marine sites from 3392 to 8341 mgL^–1^ (*p* <  0.0001; S1 Fig). TSS values were similarly higher in marine ecosystems (1551–3587 mgL^–1^) compared to estuarine (148–448 mg/L) and riverine sites (25–148 mgL^–1^) (*p* <  0.0001; S1 Fig). A strong positive correlation was observed between TDS and TSS (R^2^ =  0.945, *p* <  0.0001). Specific conductance increased significantly from riverine (179–637 µ Scm^–1^) to estuarine (6777–9714 µ Scm^–1^) to marine sites (30,085–35,782 µ Scm^–1^) (*p* <  0.0001; S1 Fig). The pH ranged from 7.47 to 7.65 in riverine, 7.71 to 7.94 in estuarine, and 7.82 to 8.07 in marine sites, with significant differences across ecosystems (*p* <  0.0001; S2 Fig). Total alkalinity increased from riverine (52–97 mgL^–1^) to marine sites (80–116 mgL^–1^; *p* <  0.01; S2 Fig). Salinity was lowest in riverine sites (908–1399 ppm) and increased in estuarine (8246–14,951 ppm) and marine (32,353–38,734 ppm) habitats (*p* <  0.0001; S2 Fig). Dissolved oxygen (DO) ranged from 6.34–8.3 mgL^–1^ in riverine, 6.72–8.32 mgL^–1^ in estuarine, and 7.19–8.26 mgL^–1^ in marine sites, with significant differences (*p* <  0.05; S2 Fig). Nitrogenous compounds showed variations, with nitrate (NO_3_⁻) concentrations of 0.12 ±  0.04 mgL^–1^ (riverine), 0.25 ±  0.09 mgL^–1^ (estuarine), and 0.4 ±  0.08 mgL^–1^ (marine) (*p* <  0.01; S2 Fig). Phosphate (PO_4_^3–^) levels increased from 0.04–0.29 mgL^–1^ in riverine to 0.26–0.52 mgL^–1^ in marine habitats (*p* <  0.001; S2 Fig). Chlorophyll-a concentrations ranged from 5.29–8.99 mgL^–1^ (riverine), 3.32–5.91 mgL^–1^ (estuarine), and 2.88–5.66 mgL^–1^ (marine), showing significant habitat-specific differences (*p* <  0.05; S2 Fig). Biological oxygen demand (BOD_5_) was lowest in marine sites (0.89–3 mgL^–1^) and highest in riverine and estuarine sites (2.63–7.97 mgL^–1^; *p* <  0.001; S3 Fig). Chemical oxygen demand (COD) varied significantly, ranging from 233.17–696.59 mgL^–1^ (riverine), 335.42–644.4 mgL^–1^ (estuarine), and 238.7–500.5 mgL^–1^ (marine; *p* <  0.01; S3 Fig).

**Table 1:** Physicochemical assessment of the three sample collection sites. This table presents the mean ±  standard error of various physicochemical parameters measured at the riverine, estuarine, and marine sample collection sites. Parameters include air temperature (°C), water temperature (°C), total dissolved solids (TDS, mgL^ − 1^), total suspended solids (TSS, mgL^ − 1^), specific conductance (µScm^ − 1^), pH, salinity (ppm), alkalinity (mgL^−1^), dissolved oxygen (DO, mgL^ − 1^), nitrogenous compounds including nitrate (NO_3_-N, mgL^ −1^), nitrite (NO_2_-N, mgL^ − 1^), and ammonium (NH_3_-N, mgL^ − 1^), phosphate (PO_4_-P, mgL^ − 1^), chlorophyll-a (µgL^ − 1^), biological oxygen demand (BOD_5_, mgL^ − 1^), and chemical oxygen demand (COD, mgL^ − 1^). The data reveal significant differences in TDS, TSS, specific conductance, and salinity across the ecosystems, with marine sites showing the highest values, reflecting the saline and particulate-rich nature of these environments.

Parameters	Riverine	Estuarine	Marine
Air Temperature (°C)	28.95 ± 2.12	28.44 ± 1.99	28.62 ± 2.48
Water Temperature (°C)	25.33 ± 2.19	25.02 ± 2.57	24.6 ± 2.83
TDS (mgL^ − 1^)	144.89 ± 66.03	1094.22 ± 323.6	5798.85 ± 1428.71
TSS (mgL^ − 1^)	91.33 ± 41.54	273.7 ± 84.18	2427.59 ± 537.36
Specific conductance (µScm^ − 1^)	398.3 ± 144.95	8122.85 ± 980.28	33436.59 ± 1695.41
pH	7.56 ± 0.06	7.80 ± 0.06	7.97 ± 0.08
Salinity (ppm)	1055.82 ± 125.93	11197.33 ± 1865.95	35540.37 ± 1954.13
Alkalinity (mgL^ − 1^)	75.52 ± 14.4	82.22 ± 10.91	97.81 ± 11.26
DO (mgL^ − 1^)	7.28 ± 0.49	7.61 ± 0.5	7.64 ± 0.32
NO_3_-N (mgL^ − 1^)	0.026 ± 0.012	0.029 ± 0.009	0.029 ± 0.012
NO_2_-N (mgL^ − 1^)	0.006 ± 0.002	0.006 ± 0.003	0.008 ± 0.003
NH_3_-N (mgL^ − 1^)	0.086 ± 0.036	0.215 ± 0.084	0.361 ± 0.082
PO_4_-P (mgL^ − 1^)	0.205 ± 0.077	0.204 ± 0.095	0.398 ± 0.082
Chlorophyll-*ɑ* (µgL^ − 1^)	7.79 ± 1.33	4.38 ± 0.82	4.34 ± 0.84
BOD_5_ (mgL^ − 1^)	5.67 ± 1.52	4.65 ± 1.73	1.95 ± 0.58
COD (mgL^ − 1^)	384.88 ± 145.39	489.58 ± 93.35	327.5 ± 71.66

TDS, total dissolved solids; TSS, total suspended solids; DO, dissolved oxygen; BOD, biological oxygen demand; COD, chemical oxygen demand.

### Blood cell indices show habitat-specific physiological adaptations in hilsa shad

**Fig 1:**
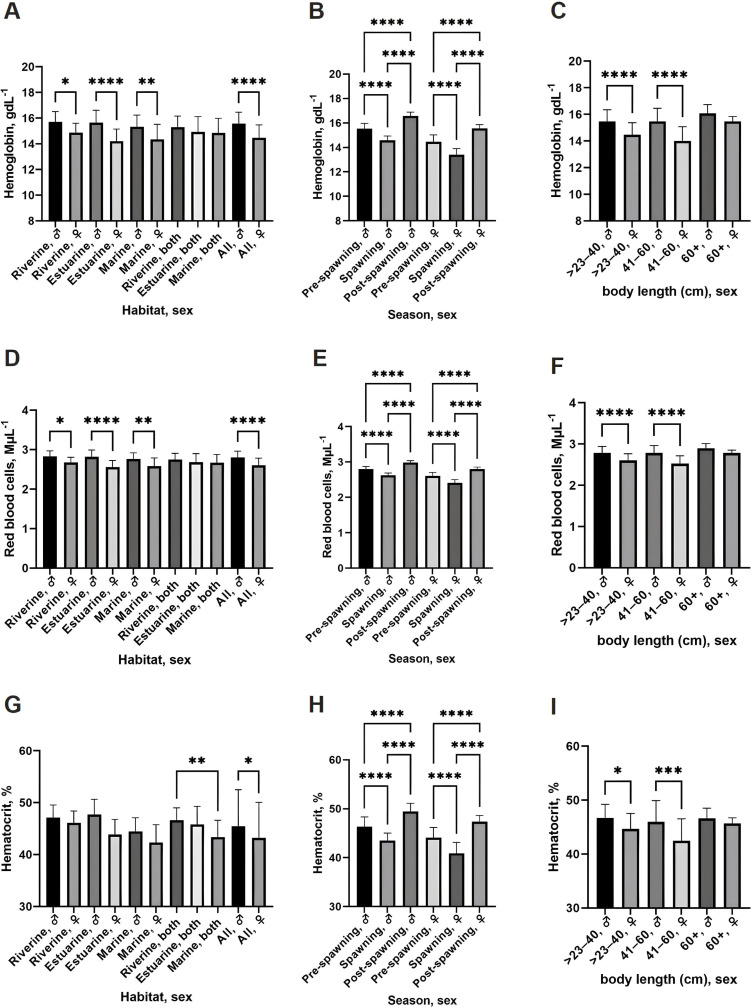
Blood cell indices of the hilsa shad collected from different ecosystems. (A–C) Hemoglobin concentrations across different habitats (A), capture season (B), and body length (C). (D–F) Red blood cell (RBC) counts across different habitats (D), capture season (E), and body length (F). (G–I) Hematocrit values across different habitats (G), capture season (H), and body length (I). Statistics, one-way ANOVA with Tukey’s multiple comparisons test; * *p* < 0.05, **p < 0.01, ***p < 0.001, ****p < 0.0001. Data are represented as mean ±  standard deviation.

The blood cell indices of hilsa shad collected from different ecosystems reveal physiological responses to varying environmental conditions (Table 2). Hemoglobin levels were slightly higher in riverine fish (15.29 ±  0.87 gdL^–1^) than in estuarine (14.92 ±  1.18 gdL^–1^) and marine fish (14.84 ±  1.14 gdL^–1^), though the differences were not statistically significant (Fig 1A). RBC counts followed a similar trend, with riverine fish exhibiting slightly higher values (2.75 ±  0.16 MµL^–1^) compared to estuarine and marine fish (Fig 1D). These data suggest that hilsa shad maintain consistent oxygen transport capacity across habitats. Post-spawning fish exhibited significantly higher hemoglobin levels (*p* <  0.0001), RBC counts (*p* <  0.0001), and hematocrit values (*p* <  0.0001) compared to pre-spawning and spawning fish, regardless of habitat (Fig 1B, 1E, 1H). Males also had significantly higher hemoglobin and RBC counts than females (*p* <  0.05; Fig 1A, 1D). No significant correlations were observed between body length and hemoglobin, RBC count, or hematocrit values (Fig 1C, 1F, 1I). Hematocrit levels were significantly higher in riverine fish (46.61 ±  2.37%) compared to marine fish (43.4 ±  3.19%) (*p* <  0.01; Fig 1G). Spawning season significantly influenced hematocrit levels, with post-spawning fish exhibiting the highest value across all habitats (*p* <  0.0001; Fig 1H). The reduction in hematocrit during the spawning season, followed by an increase post-spawning, suggests a seasonal modulation of oxygen transport efficiency.

**Table 2:** Blood cell indices in hilsa shad caught from different ecosystems. This table presents various blood cell indices, including hemoglobin levels (gdl^ − 1^), red blood cell counts (RBC, MµL^ − 1^), RBC morphometrics (length, width, surface area), nuclear morphometrics (length, width, surface area), hematocrit (%), mean corpuscular volume (MCV, fL), mean corpuscular hemoglobin concentration (MCHC, gdL^ − 1^), mean corpuscular hemoglobin (MCH, pg), white blood cell counts (WBC, KµL^ − 1^), and platelet counts (KµL^ − 1^). The data reveal habitat-specific differences, particularly in hematocrit values, which were higher in riverine fish, and in WBC counts, which were higher in females and in fish collected during the post-spawning season.

Parameters	Riverine	Estuarine	Marine	Overall
Hemoglobin (gdL^ − 1^)	15.29 ± 0.87	14.92 ± 1.18	14.84 ± 1.14	15.02 ± 1.09
RBC (MµL^ − 1^)	2.75 ± 0.16	2.68 ± 0.21	2.67 ± 0.20	2.70 ± 0.2
Length (µm)	13.83 ± 1.08	13.55 ± 1.13	13.43 ± 1.05	13.61 ± 1.1
Width (µm)	9.22 ± 0.72	9.03 ± 0.75	8.95 ± 0.7	9.06 ± 0.74
Surface area (µm^2^)	100.8 ± 15.61	96.8 ± 15.96	95.06 ± 15	97.55 ± 15.71
Length of nucleus (µm)	5.63 ± 0.52	5.52 ± 0.53	5.4 ± 0.49	5.52 ± 0.52
Width of nucleus (µm)	3.65 ± 0.51	3.52 ± 0.5	3.42 ± 0.49	3.53 ± 0.51
Surface area of nucleus (µm^2^)	16.34 ± 0.67	15.43 ± 3.55	14.67 ± 3.44	15.49 ± 3.62
Hematocrit	46.61 ± 2.37	45.80 ± 3.45	43.4 ± 3.19	45.27 ± 3.33
MCV (fL)	169.46 ± 2.78	170.57 ± 1.11	162.51 ± 1.39	167.51 ± 4.04
MCHC (gdL^ − 1^)	32.8 ± 0.54	32.57 ± 0.21	34.19 ± 0.29	33.19 ± 0.81
MCH (pg)	55.61 ± 2.27	55.67 ± 2.5	55.58 ± 2.07	55.62 ± 2.29
WBC (KµL^ − 1^)	4.75 ± 0.33	4.64 ± 0.42	4.62 ± 0.41	4.67 ± 0.39
Lymphocytes (%)	83.77 ± 3.83	82.63 ± 4.6	83.53 ± 3.51	83.31 ± 4.03
Eosinophils (%)	9.03 ± 3.78	10.18 ± 4.15	9.21 ± 4.32	9.48 ± 4.12
Basophils (%)	3.45 ± 1.67	3.73 ± 1.69	3.58 ± 1.77	3.59 ± 1.72
Neutrophils (%)	2.38 ± 1.44	2.26 ± 1.48	2.53 ± 1.47	2.37 ± 1.47
Lymphoblasts (%)	1.37 ± 1.02	1.18 ± 1.09	1.13 ± 0.99	1.23 ± 1.04
Platelets (KµL^ − 1^)	66.63 ± 5.26	56.42 ± 6.22	69.64 ± 6.4	64.23 ± 8.23

RBC, red blood cells; MCV, mean corpuscular volume; MCHC, mean corpuscular hemoglobin concentration; MCH, mean corpuscular hemoglobin; WBC, white blood cells.

Measurements of RBC length, width, and surface area, as well as nuclear dimensions, showed no significant differences across habitats, sexes, or body lengths (S4 Fig). However, males within the 23–40 cm body length group had a significantly larger nuclear surface area than those in the 41–60 cm group (*p* <  0.05; S4 Fig). These findings indicate that hilsa shad maintain a uniform RBC morphology despite varying environmental conditions.

**Fig 2:**
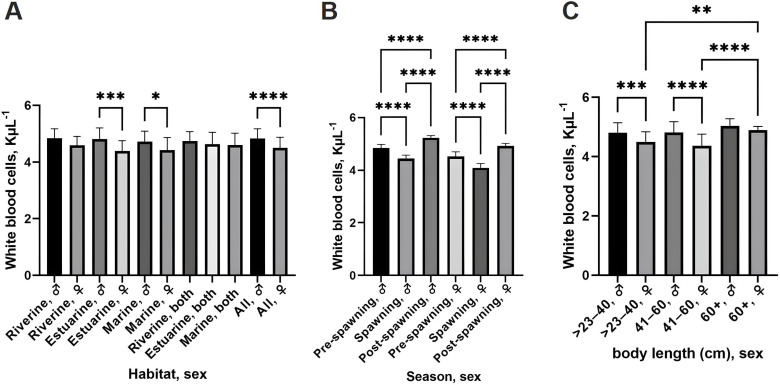
White blood cell (WBC) counts of the hilsa shad collected from different ecosystems. (A) WBC counts across different habitats and sexes. (B) WBC counts across different capture seasons. (C) WBC counts across different body lengths and sexes. Statistics, one-way ANOVA with Tukey’s multiple comparisons test; * *p* < 0.05, **p < 0.01, ***p < 0.001, ****p < 0.0001. Data are represented as mean ±  standard deviation.

WBC counts varied significantly between sexes and across habitats. Females had consistently higher WBC counts than males, particularly in estuarine and marine environments (*p* <  0.0001; Fig 2A). WBC counts were significantly elevated in post-spawning fish (*p* <  0.0001; Fig 2B), suggesting a heightened immune response during the recovery phase. Size also influenced WBC counts, with the highest values observed in females > 60 cm, particularly in estuarine habitats (*p* <  0.0001; Fig 2C). These variations indicate that WBC levels are influenced by sex, habitat, and body size.

**Fig 3:**
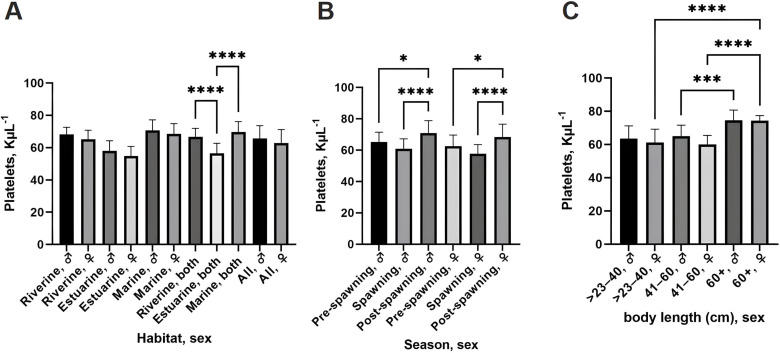
Platelet counts of the hilsa shad collected from different ecosystems. (A) Platelet counts across different habitats and sexes. (B) Platelet counts across different capture seasons. (C) Platelet counts across different body lengths and sexes. Statistics, one-way ANOVA with Tukey’s multiple comparisons test; * *p* < 0.05, **p < 0.01, ***p < 0.001, ****p < 0.0001. Data are represented as mean ±  standard deviation.

Platelet counts differed significantly across habitats, with estuarine fish exhibiting lower counts than riverine and marine fish (*p* <  0.0001; Fig 3A). Post-spawning fish had significantly higher platelet counts compared to pre-spawning and spawning fish (*p* <  0.0001; Fig 3B). Females in the 60^ +^ cm group had significantly elevated platelet counts compared to smaller individuals (*p* <  0.0001; Fig 3C). Males in the same size group also exhibited significantly higher platelet counts than those in the 41–60 cm group (*p* <  0.001).

Overall, these results reveal unique physiological adaptations in hilsa shad based on habitat and reproductive stage. Riverine fish had higher hematocrit values, indicating better oxygen transport in freshwater. Consistent RBC morphology suggests a stable oxygen transport strategy, while variations in WBC and platelet counts relate to immune function and coagulation influenced by sex, habitat, and reproductive stage.

### Serum biochemistry and electrolyte profiles reveal habitat-specific metabolic and stress responses in hilsa shad

The serum biochemistry and electrolyte profiles of hilsa shad provide insights into metabolic adjustments, stress responses, and osmoregulatory strategies across different habitats (Table 3). Alanine aminotransferase (ALT) and aspartate aminotransferase (AST) are widely used indicators of liver function and metabolic activity. ALT levels were significantly higher in females during the spawning season compared to pre-spawning (*p* <  0.05), suggesting increased liver activity associated with reproductive metabolism (S5 Fig). Additionally, females in the 41–60 cm body length group exhibited significantly higher ALT levels than males of the same size class (*p* <  0.01), indicating potential sex-specific metabolic demands during reproduction (S5 Fig). However, AST levels did not show significant variation across habitats, sexes, or body length groups (S5 Fig), suggesting a relatively stable hepatic function despite environmental fluctuations. Total protein and albumin levels remained relatively consistent across habitats (S5 Fig). However, albumin was significantly elevated in spawning females (*p* <  0.01), likely reflecting increased protein synthesis for vitellogenesis. In contrast, albumin levels were significantly lower in females of the 60^ +^ cm body length group than those in smaller size classes (*p* <  0.001), suggesting an age-related decline in protein synthesis capacity or metabolic efficiency (S5 Fig). The moderate increase in albumin levels in females of the 41–60 cm group compared to males (*p* <  0.05) suggests that mid-sized females experience heightened metabolic demands during reproduction. Globulin levels, which indicate immune function, were significantly elevated during the spawning season compared to pre-spawning (*p* <  0.05), potentially reflecting an enhanced immune response during reproductive activity (S5 Fig).

**Table 3:** Serum biochemistry and electrolyte profile in hilsa shad collected from different ecosystems. This table presents various serum biochemistry and electrolyte parameters, including alanine aminotransferase (ALT, IUL^ − 1^), aspartate aminotransferase (AST, IUL^ − 1^), total protein (gdL^ − 1^), albumin (gdL^ − 1^), globulin (gdL^ − 1^), total cholesterol (TC, mgdL^ − 1^), creatinine (mgdL^ − 1^), cortisol (µgdL^ − 1^), sodium (mEqL^ − 1^), chloride (mEqL^ − 1^), potassium (mEqL^ − 1^), calcium (mgdL^ − 1^), magnesium (mgdL^ − 1^), phosphate (mEqL^ − 1^), glucose (mgdL^ − 1^), and uric acid (mgdL^ − 1^). The data reveal significant habitat-specific differences, particularly in cortisol levels, which were markedly higher in riverine fish, and in sodium and potassium levels, reflecting the osmoregulatory challenges faced by hilsa shad in different environments.

Parameters	Riverine	Estuarine	Marine	Overall
ALT (IUL^ − 1^)	15.88 ± 4.95	14.97 ± 4.34	15.12 ± 4.01	15.32 ± 4.47
AST (IUL^ − 1^)	18.07 ± 5.87	17.23 ± 5.32	17.83 ± 5.01	17.71 ± 5.42
Total protein (gdL^ − 1^)	2.96 ± 0.32	2.97 ± 0.26	2.9 ± 0.3	2.95 ± 0.3
Albumin (gdL^ − 1^)	0.8 ± 0.03	0.81 ± 0.03	0.81 ± 0.3	0.81 ± 0.3
Globulin (gdL^ − 1^)	1.08 ± 0.15	1.1 ± 0.09	1.13 ± 0.14	1.1 ± 0.13
TC (mgdL^ − 1^)	173.6 ± 17.92	162.17 ± 15.82	165.38 ± 26.93	167.05 ± 21.34
Creatinine (mgdL^ − 1^)	0.12 ± 0.02	0.13 ± 0.02	0.12 ± 0.02	0.12 ± 0.02^δ^
Cortisol (µgdL^ − 1^)	67.52 ± 41.08^δδδδ^^,^ ^ξξξξ^	24.23 ± 11.49	23.12 ± 8.45	38.29 ± 32.52^φφφφ^^,^ ^δδ^^,^ ^ξξ^
Sodium (mEqL^ − 1^)	135.6 ± 4.14	136.82 ± 5.27	141.02 ± 6.62	137.81 ± 5.91^φ^^,^ ^ξξ^
Chloride (mEqL^ − 1^)	107.23 ± 5	108.62 ± 3.87	112.08 ± 3.31	109.31 ± 4.6^φφ^^,^ ^ξξξ^
Potassium (mEqL^ − 1^)	2.8 ± 0.23	2.8 ± 0.3	2.7 ± 0.32	2.77 ± 0.29
Calcium (mgdL^ − 1^)	9.52 ± 0.46	9.54 ± 0.45	9.57 ± 0.38	9.54 ± 0.43
Magnesium (mgdL^ − 1^)	3 ± 0.28	2.97 ± 0.24	3.01 ± 0.26	3 ± 0.26
Phosphate (mEqL^ − 1^)	6.78 ± 0.89	6.61 ± 0.94	7.32 ± 0.82	6.9 ± 0.93^ξ^
Glucose (mgdL^ − 1^)	33.03 ± 8.05	34.6 ± 7.25	34.73 ± 7.36	34.12 ± 7.6
Uric Acids (mgdL^ − 1^)	3.91 ± 0.93	3.88 ± 0.92	4.14 ± 0.86	3.98 ± 0.91

ALT, alanine aminotransferase; AST, aspartate aminotransferase; TC, total cholesterols.

δ significantly different than the fish caught from estuarine ecosystems at *p* <  0.05;

δδ significantly different than the fish caught from estuarine ecosystems at *p* <  0.01;

δδδδ significantly different than the fish caught from estuarine ecosystems at *p* <  0.0001;

ξξ significantly different than the fish caught from marine ecosystems at *p* <  0.01;

ξξξ significantly different than the fish caught from marine ecosystems at *p* <  0.001;

ξξξξ significantly different than the fish caught from marine ecosystems at *p* <  0.0001;

φ significantly different than the fish caught from riverine ecosystems at *p* <  0.05;

φφ significantly different than the fish caught from riverine ecosystems at *p* <  0.01;

φφφφ significantly different than the fish caught from riverine ecosystems at *p* <  0.0001.

Total cholesterol levels did not differ significantly among habitats, suggesting a conserved lipid metabolism in hilsa shad (S5 Fig). However, cholesterol levels were significantly higher in post-spawning fish than in pre-spawning and spawning individuals (*p* <  0.001), likely due to lipid mobilization during recovery (S5 Fig). Larger fish (41–60 cm and 60^ +^ cm) exhibited significantly higher cholesterol levels (*p* <  0.05), suggesting greater lipid reserves to meet increased energy demands associated with growth and reproduction.

Uric acid levels remained stable across habitats and body length groups, indicating consistent nitrogen metabolism in hilsa shad (S5 Fig). However, uric acid levels were significantly higher in spawning females than in pre- and post-spawning fish (*p* <  0.05), potentially reflecting increased protein catabolism or stress-related metabolic shifts during reproduction. Cortisol, a key stress hormone, exhibited the most pronounced differences among the measured biochemical parameters. Riverine fish, particularly females, had significantly higher cortisol levels compared to estuarine and marine populations (*p* <  0.0001; S5 Fig). Spawning females exhibited significantly elevated cortisol levels compared to pre-spawning individuals (*p* <  0.05), suggesting reproduction imposes a significant physiological stress burden (S5 Fig). Smaller females (23–40 cm) had significantly higher cortisol levels than larger females (*p* <  0.0001), indicating that younger or smaller individuals may experience greater stress during spawning.

Serum electrolyte profiles reflect osmoregulatory adjustments as hilsa shad migrate between freshwater, estuarine, and marine habitats (S6 Fig). Males had significantly higher sodium levels than females across all habitats (*p* <  0.01; S6 Fig). Post-spawning fish exhibited significantly elevated sodium levels compared to pre-spawning individuals (*p* <  0.0001), suggesting increased osmoregulatory demands post-reproduction (S6 Fig). Chloride levels were significantly higher in marine-caught fish than in estuarine and riverine populations (*p* <  0.0001), as expected due to higher salinity in marine environments (S6 Fig). Chloride levels were significantly lower in females post-spawning compared to during spawning (*p* <  0.05), indicating potential osmoregulatory adjustments linked to reproductive state (S6 Fig). Males in the 41–60 cm group had significantly higher chloride levels than smaller individuals (*p* <  0.05), suggesting size-dependent differences in osmoregulatory capacity (S6 Fig). Potassium levels were significantly higher in spawning females than in males (*p* <  0.05), possibly reflecting potassium’s role in reproductive physiology and stress response (S6 Fig). Females in the 23–40 cm group exhibited significantly higher potassium levels than larger females (*p* <  0.0001), suggesting differential osmoregulatory requirements across size classes (S6 Fig). These parameters did not show significant variations across habitats, sexes, seasons, or body lengths (S6 Fig). However, phosphate levels were slightly elevated in marine-caught fish (*p* <  0.05), potentially due to increased dietary availability in marine environments (S6 Fig).

The serum biochemistry and electrolyte profiles of hilsa shad show metabolic adjustments related to habitat and reproduction. Spawning females exhibit elevated liver enzymes and albumin levels, indicating increased metabolic demands. Higher cortisol levels in riverine fish suggest stress from environmental changes. Variations in sodium and potassium levels highlight the osmoregulatory challenges hilsa shad face in transitioning between freshwater, estuarine, and marine environments.

## Discussion

The upstream spawning migration is a critical event in the life cycle of anadromous fish, like hilsa shad or *T. ilisha*, playing an essential role in their reproductive success [35]. This process is highly energy-demanding, requiring fish to cease feeding and rely on stored energy reserves [36]. Many anadromous species compensate for these challenges by altering their metabolic enzyme systems, cardiovascular capacity, and energy utilization patterns to optimize swimming efficiency and reproductive outcomes [36,37]. However, despite the economic and ecological importance of hilsa shad, the physiological mechanisms underlying these adaptations remain largely unexplored [37,38]. Our study aimed to fill this gap by providing a comprehensive analysis of the physiological profiles of hilsa shad across diverse ecological habitats in Bangladesh. By incorporating hematological, biochemical, and electrolyte analyses, we demonstrate how hilsa shad navigates environmental fluctuations during migration and spawning. This study highlights critical habitat-specific physiological variations, particularly in oxygen transport efficiency, immune responses, liver function, and osmoregulatory balance, which are key determinants of reproductive success.

Environmental parameters such as temperature, dissolved oxygen, salinity, and pollution levels play a crucial role in shaping fish physiology [37–40]. Our assessment revealed that hilsa shad encounter significant physicochemical variations across riverine, estuarine, and marine habitats (Table 1, S1–S3 Figs). Previous studies have emphasized that water quality influences migration success, spawning behavior, and larval survival [41–43]. our findings suggest that moderate pollution levels in riverine and estuarine sites could impose additional physiological stress, potentially affecting migration and reproductive success (Table 1, S3 Fig) [40,44,45]. These results emphasize the need for improved water quality management to sustain hilsa populations and mitigate non-fishing-related stressors, such as siltation and pollution [40].

Oxygen transport efficiency is a critical determinant of migration endurance. Our study found significant variations in hematocrit, hemoglobin, and red blood cell counts across habitats, seasons, and body sizes (Table 2, Fig 1, S4 Fig). Hilsa shad from riverine habitats exhibited higher Hct and RBC counts, indicating an adaptation to lower dissolved oxygen levels (Fig 1). This is consistent with studies on other active migratory species, where increased hematocrit enhances oxygen transport during strenuous activities [20,46–54]. Interestingly, post-spawning hilsa exhibited significantly higher hemoglobin and RBC counts, aligning with patterns observed in *Schizothorax plagiostomus*, *Clarias batrachus*, and *Tor putitora*, where males or post-spawning individuals demonstrated greater hematological values due to higher metabolic demands [19,55]. This suggests that hilsa shad rely on enhanced oxygen-carrying capacity to meet the intense metabolic demands of migration and reproduction [48,49].

Surprisingly, despite environmental differences, RBC morphology remained stable across habitats (S7 Fig). In contrast to other species, where environmental stress induces erythrocyte deformability, hilsa shad appear to maintain cellular integrity, highlighting a robust physiological mechanism to sustain efficient gas exchange [14,20,50,56–63]. The small RBC size and nuclear dimensions further support this, as they align with high oxygen demand and sustained aerobic metabolism during migration [64].

Immune responses in fish are shaped by both intrinsic factors, e.g., sex, size, and reproductive stage, and extrinsic factors, e.g., habitat, and environmental stressors [65,66]. Our findings indicate that WBC counts were significantly higher in females, particularly in estuarine and marine populations (Fig 2). This pattern aligns with previous research on *S. labiatus* and *C. batrachus*, where higher WBC counts in females suggest enhanced immune responses to environmental stress [20,67]. Also, WBC counts peaked in post-spawning individuals, supporting the hypothesis that spawning is an immunosuppressive event, requiring subsequent immune activation during recovery (Fig 2). This is consistent with the observed hematocrit and hemoglobin trends, where oxygen transport capacity also rebounds post-spawning (Fig 3). These findings highlight the trade-off between reproduction and immune function, a phenomenon well-documented in teleost fish [68]. However, further research is needed to fully understand the mechanisms underlying these changes and their implications for the health and reproductive success of hilsa shad. Platelet, counts on the other hand, also varied by habitat and size, with lower counts in estuarine fish compared to riverine and marine counterparts (Fig 3). Post-spawning females, particularly larger ones (60^ +^ cm), had higher platelet counts, aligning with the need for coagulation and wound healing during and after spawning [69]. This trend mirrors patterns observed in other species like *H. longifilis* and R*. ventralis*, highlighting the complex interplay between sex, reproductive status, and environmental conditions in shaping hematological profiles [54,70].

Serum biochemistry provides a direct measure of metabolic function and stress levels in fish. Our study identified habitat-specific variations in liver enzyme activity, lipid metabolism, and stress hormones, reflecting distinct physiological challenges faced by hilsa shad (Table 3, S5 Fig). ALT activity was significantly elevated in spawning females, indicating increased hepatic activity for vitellogenesis (S5 Fig). Similar trends have been reported in *S. labiatus*, where reproductive females exhibited higher liver enzyme activity during egg production [71]. Cholesterol levels were higher in post-spawning fish, suggesting lipid mobilization for recovery after migration and spawning (S5 Fig) [72,73]. Elevated creatinine levels in estuarine fish suggest increased renal stress, potentially due to fluctuating salinity and osmotic challenges (S5 Fig) [74,75]. Cortisol levels were highest in riverine fish, particularly during spawning, highlighting the intense physiological stress imposed by upstream migration (S5 Fig) [74,75].

Hilsa shad encounter significant osmoregulatory challenges as they transition between freshwater, estuarine, and marine environments. Sodium and potassium levels were significantly elevated in post-spawning individuals, suggesting an increased osmoregulatory burden during recovery (S6 Fig). Elevated potassium in spawning females may indicate its role in reproductive physiology (S6 Fig) [72,74,75]. Our findings align with previous studies showing electrolyte imbalances as key indicators of stress and reproductive activity in anadromous fish (S6 Fig) [21,76,77]. The relative stability of calcium, magnesium, and glucose levels suggests that hilsa shad maintain homeostasis efficiently despite varying environmental conditions (S6 Fig).

This study presents several novel aspects. It provides a comprehensive physiological profile of hilsa shad across different habitats and seasons, with a focus on hematology, serum biochemistry, and electrolyte balance—areas previously underexplored for the case of hilsa shad [2,4,7,40,42,43,45,72,78,79]. The use of a mobile laboratory for on-site blood sampling is a unique methodological advancement, allowing for the collection of minimally stressed, high-quality data that more accurately reflects the natural physiological state of the fish. This study is also the first to directly link specific physiological changes, e.g., liver enzyme activity and electrolyte profiles, to the fish’s migratory and reproductive behaviors, offering new insights into the adaptive strategies that support the species’ survival in diverse and challenging environments.

A major strength of this study lies in its multidisciplinary approach, integrating environmental assessments with detailed physiological profiling. This holistic analysis provides a thorough understanding of the factors influencing hilsa shad’s health and reproductive success. The on-site blood sampling method minimizes stress and ensures the accuracy of the physiological data collected. Additionally, the study’s focus on seasonal and habitat-specific variations offers valuable insights into how hilsa shad cope with different environmental challenges, enhancing our understanding of their adaptive strategies. The comparison with existing literature further situates these findings within the broader context of fish physiology and migratory biology. However, the study has some limitations as well. While on-site sampling minimizes stress, it does not eliminate completely handling effects on physiological parameters. The cross-sectional design, though useful for identifying associations, limits the ability to draw causal inferences about observed physiological changes. Longitudinal studies tracking individual fish through different stages of migration and spawning would provide deeper insights into these processes. Additionally, the study’s geographic scope, though comprehensive within Bangladesh, does not cover the full migratory range of hilsa shad across the inland water bodies and the Bay of Bengal. Including additional populations across their full migratory range would offer a more complete understanding of the species’ physiological adaptations. Finally, the focus on adult fish may not fully capture the physiological challenges faced by juvenile or early life stages, which are crucial for understanding the species’ life cycle.

Building on the findings of this study, future research should focus on longitudinal studies to track hilsa shad across different life stages and migratory routes, providing insights into physiological adaptation over time. Expanding the geographic scope to include other regions within the species’ migratory range would enhance our understanding of adaptive diversity. Additionally, exploring the physiological profiles of juvenile stages is crucial for understanding the full life cycle and ensuring population sustainability. Investigating the genetic basis of physiological adaptations could shed light on the evolutionary processes that enable hilsa shad to thrive in diverse environments. Integrating physiological data with ecological modeling would help predict population responses to environmental changes, guiding conservation and management strategies for this culturally and economically vital species.

Overall, this study offers a novel and comprehensive analysis of the physiological adaptations of hilsa shad across diverse habitats and seasons, emphasizing the crucial role of hematological, biochemical, and electrolyte profiles in their migratory and reproductive success. The findings highlight how these physiological mechanisms help hilsa shad navigate the significant metabolic and stress-related challenges of migration and spawning. The use of a mobile laboratory for on-site sampling and a multidisciplinary approach enhances data accuracy and relevance, providing key insights into the species’ adaptive strategies. Future research should focus on longitudinal studies and broader geographic analysis to fully understand hilsa shad physiology across their migratory range, which is essential for effective conservation and management strategies in the face of environmental changes.

## Supporting information

S1 Fig Air and water temperatures during sample collection. (A) Air temperature across different sampling sites in riverine, estuarine, and marine ecosystems. (B) Water temperature across the same sampling sites. (C) Total dissolved solids (TDS) levels in the sampled habitats. (D) Total suspended solids (TSS) across different sites. (E) Conductivity levels in the sampled water. Statistics, one-way ANOVA with Tukey’s multiple comparisons test; **p < 0.01, **** *p*  < 0.0001. Data are represented as mean ±  standard deviation.
(TIF)

S2 Fig Water pH and other related parameters during sample collection. (A) Water pH at the sampling sites. (B) Salinity levels across riverine, estuarine, and marine sites. (C) Alkalinity measurements at the different sampling locations. (D) Dissolved oxygen (DO) content across habitats. (E–H) Concentrations of various nitrogenous compounds (NO_3_-N, NO_2_-N, NH_3_-N, and PO_4_-P) at the sampling sites. Statistics, one-way ANOVA with Tukey’s multiple comparisons test; *  *p*  < 0.05, ** *p*  < 0.01, **** *p*  < 0.0001. Data are represented as mean ±  standard deviation.
(TIF)

S3 Fig Biological oxygen demand and other biochemical parameters in the sampled water. (A) Chlorophyll-a levels across different sites. (B) Biological oxygen demand (BOD_5_) across habitats. (C) Chemical oxygen demand (COD) levels in riverine, estuarine, and marine ecosystems. Statistics, one-way ANOVA with Tukey’s multiple comparisons test; ** *p*  < 0.01, **** *p*  < 0.0001. Data are represented as mean ±  standard deviation.
(TIF)

S4 Fig Red blood cell (RBC) and nuclear morphometrics of the hilsa shad collected from different ecosystems during different seasons. (A–C) RBC lengths across different habitats (A), capture seasons (B), and body lengths (C). (D–F) RBC width across habitats (D), seasons (E), and body lengths (F). (G–I) RBC surface area across different habitats (G), seasons (H), and body lengths (I). (J–L) Nucleus length across different habitats (J), seasons (K), and body lengths (L). (M–O) Nucleus width across habitats (M), seasons (N), and body lengths (O). (P–R) Nucleus surface area across different habitats (P), seasons (Q), and body lengths (R). Statistics, one-way ANOVA with Tukey’s multiple comparisons test; *  *p*  < 0.05. Data are represented as mean ±  standard deviation.
(TIF)

S5 Fig Serum biochemistry profile of the hilsa shad collected from different ecosystems during different seasons. (A–C) ALT levels across habitats (A), capture seasons (B), and body lengths (C). (D–F) AST levels across habitats (D), seasons (E), and body lengths (F). (G–I) Total protein levels across different habitats (G), seasons (H), and body lengths (I). (J–L) Albumin levels across habitats (J), seasons (K), and body lengths (L). (M–O) Globulin levels across habitats (M), seasons (N), and body lengths (O). (P–R) Total cholesterol levels across habitats (P), seasons (Q), and body lengths (R). (S–U) Creatinine levels across habitats (S), seasons (T), and body lengths (U). (V–X) Uric acid levels across habitats (V), seasons (W), and body lengths (X). (Y–AA) Cortisol levels across different habitats (Y), body lengths (Z), and seasons (AA). Statistics, one-way ANOVA with Tukey’s multiple comparisons test; *  *p*  < 0.05, ** *p*  < 0.01, *** *p*  < 0.001, **** *p*  < 0.0001. Data are represented as mean ±  standard deviation.
(JPG)

S6 Fig Serum electrolyte profile of the hilsa shad collected from different ecosystems during different seasons. (A–C) Sodium levels across habitats (A), seasons (B), and body lengths (C). (D–F) Chloride levels across habitats (D), seasons (E), and body lengths (F). (G–I) Potassium levels across habitats (G), seasons (H), and body lengths (I). (J–L) Calcium levels across habitats (J), seasons (K), and body lengths (L). (M–O) Magnesium levels across habitats (M), seasons (N), and body lengths (O). (P–R) Phosphate levels across habitats (P), seasons (Q), and body lengths (R). (S) Glucose levels across body lengths and seasons. Statistics, one-way ANOVA with Tukey’s multiple comparisons test; *  *p*  < 0.05, ** *p*  < 0.01, *** *p*  < 0.001, **** *p*  < 0.0001. Data are represented as mean ±  standard deviation.
(JPG)

S7 Fig A comparative analysis of erythrocyte dimensions among various fish species. The length (A, D), width (B, E), and size (C, F) of both erythrocytes (A–C) and their nuclei (D–F) are presented. The species examined include *Tenualosa ilisha* (Hilsa Shad), *Acipenser sinensis* (Chinese Sturgeon), *Blennius sanguinolentus* (Black Sea Blenny), *Carassius auratus auratus* (Gold Crucian Carp, Goldfish), *Dasyatis pastinaca* (Common Stingray), *Gadus morhua* (Atlantic Cod), *Gaidropsarus mediterraneus* (Shore Rockling), *Glyptosternon maculatum* (Regan, Barkley), *Lophius piscatorius* (Angler, European Angler), *Neogobius melanostomus* (Round Goby), *Nematalosa erebi* (Australian River Gizzard Shad, Bony Bream), *Oncorhynchus keta* (Chum Salmon), *Petromyzon marinus* (Sea Lamprey), *Raja clavata* (Thornback Ray), *Salvelinus namaycush* (Lake Trout), *Schizopyge niger* (Alghad Snowtrout), *Schizopyge plagiostomus* (Snow Trout), *Scorpaena porcus* (Black Scorpionfish), *Trachurus mediterraneus ponticus* (Horse Mackerel), and *Xiphias gladius* (Swordfish, Broadbill).
(JPG)

S1 Data Source data. (PDF)
