# Supplementary figures and images for: Biophysical assessments and blood profiling reveal physiological adaptations and environmental interactions of hilsa shad (Tenualosa ilisha)
Source: PLoS One. 2025 Apr 1;20(4):e0320628. doi: 10.1371/journal.pone.0320628 (PMC11960910; doi:10.1371/journal.pone.0320628)

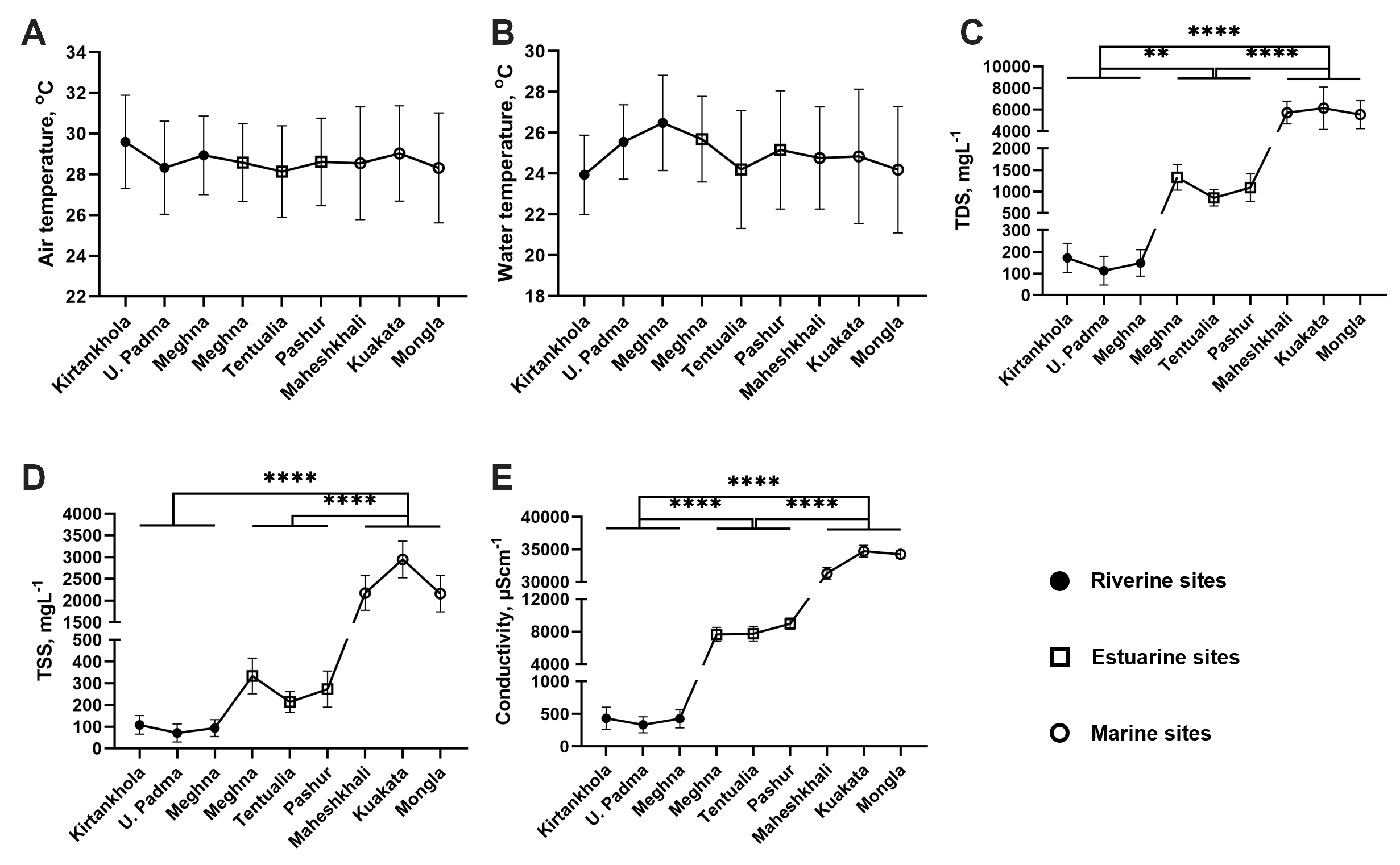

Supplement: S1 Fig — (A) Air temperature across different sampling sites in riverine, estuarine, and marine ecosystems. (B) Water temperature across the same sampling sites. (C) Total dissolved solids (TDS) levels in the sampled habitats. (D) Total suspended solids (TSS) across different sites. (E) Conductivity levels in the sampled water. Statistics, one-way ANOVA with Tukey’s multiple comparisons test; **p < 0.01, **** p < 0.0001. Data are represented as mean ± standard deviation. (TIF) [file pone.0320628.s001.tif]

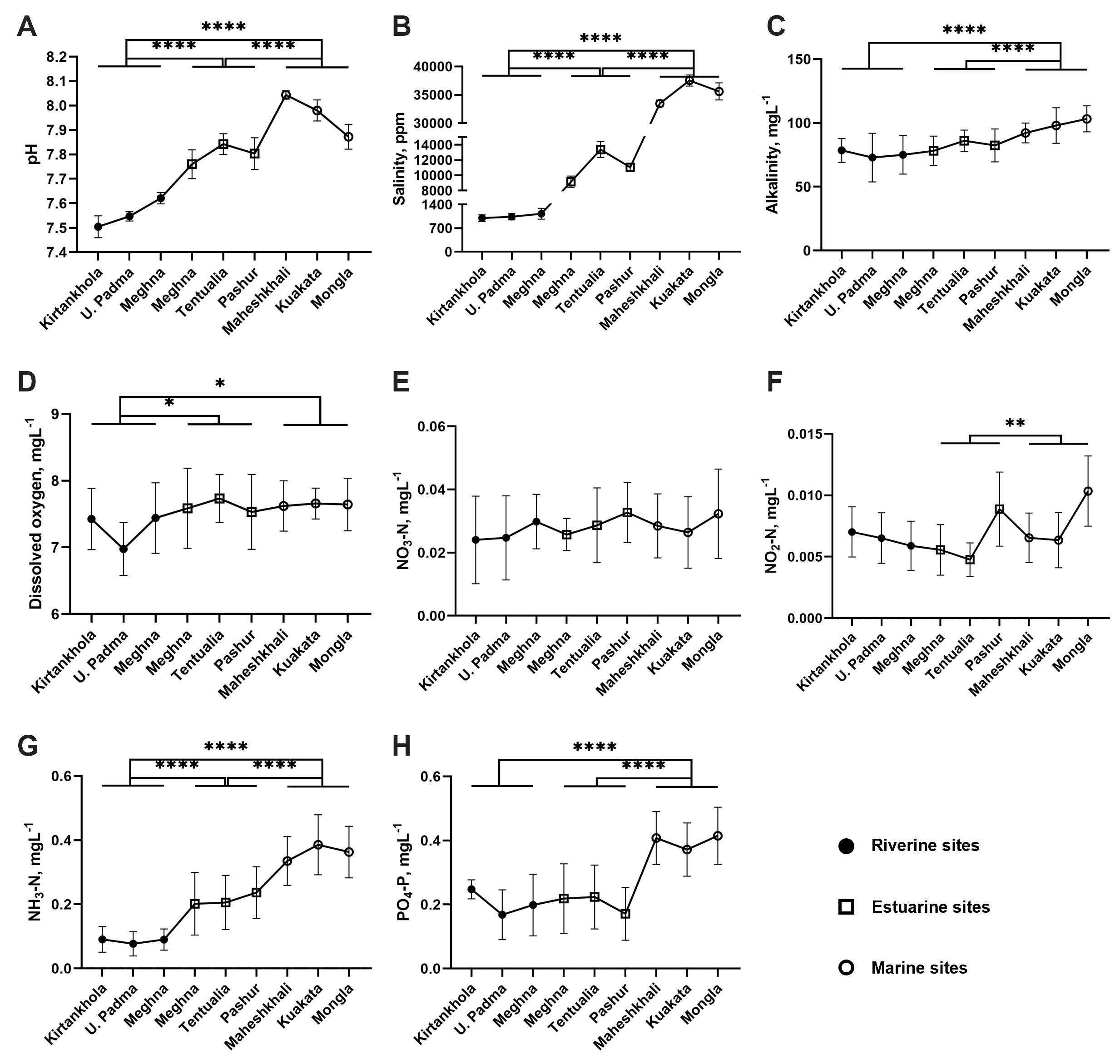

Supplement: S2 Fig — (A) Water pH at the sampling sites. (B) Salinity levels across riverine, estuarine, and marine sites. (C) Alkalinity measurements at the different sampling locations. (D) Dissolved oxygen (DO) content across habitats. (E–H) Concentrations of various nitrogenous compounds (NO3-N, NO2-N, NH3-N, and PO4-P) at the sampling sites. Statistics, one-way ANOVA with Tukey’s multiple comparisons test; * p < 0.05, ** p < 0.01, **** p < 0.0001. Data are represented as mean ± standard deviation. (TIF) [file pone.0320628.s002.tif]

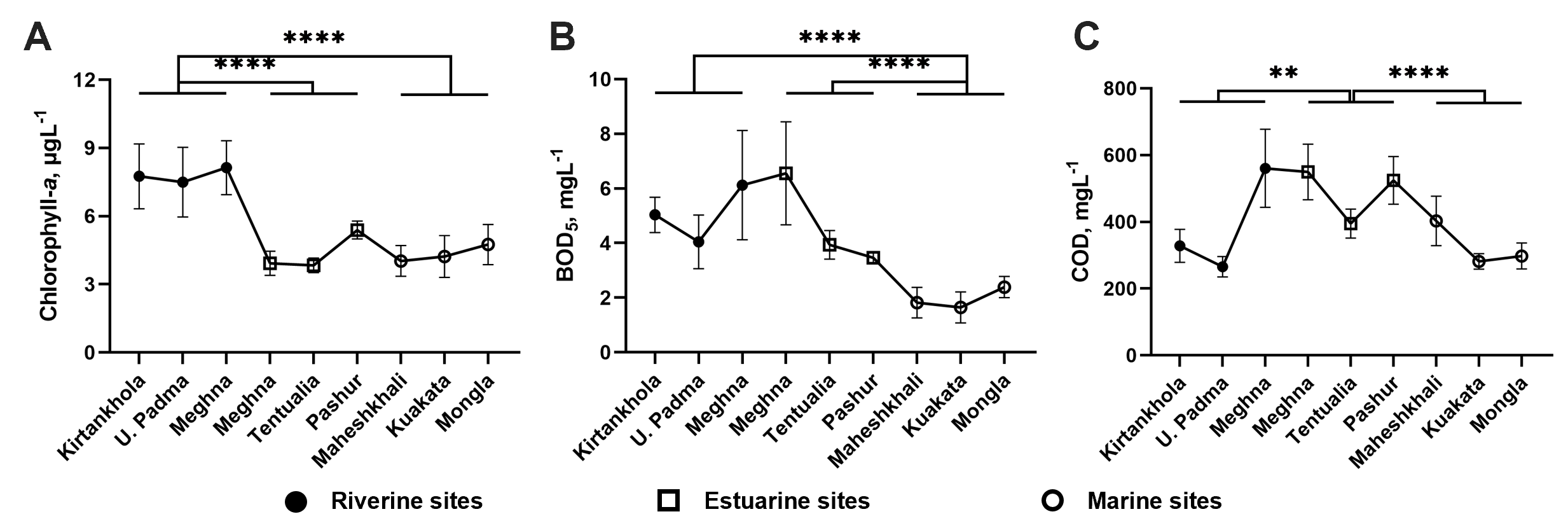

Supplement: S3 Fig — (A) Chlorophyll-a levels across different sites. (B) Biological oxygen demand (BOD5) across habitats. (C) Chemical oxygen demand (COD) levels in riverine, estuarine, and marine ecosystems. Statistics, one-way ANOVA with Tukey’s multiple comparisons test; ** p < 0.01, **** p < 0.0001. Data are represented as mean ± standard deviation. (TIF) [file pone.0320628.s003.tif]

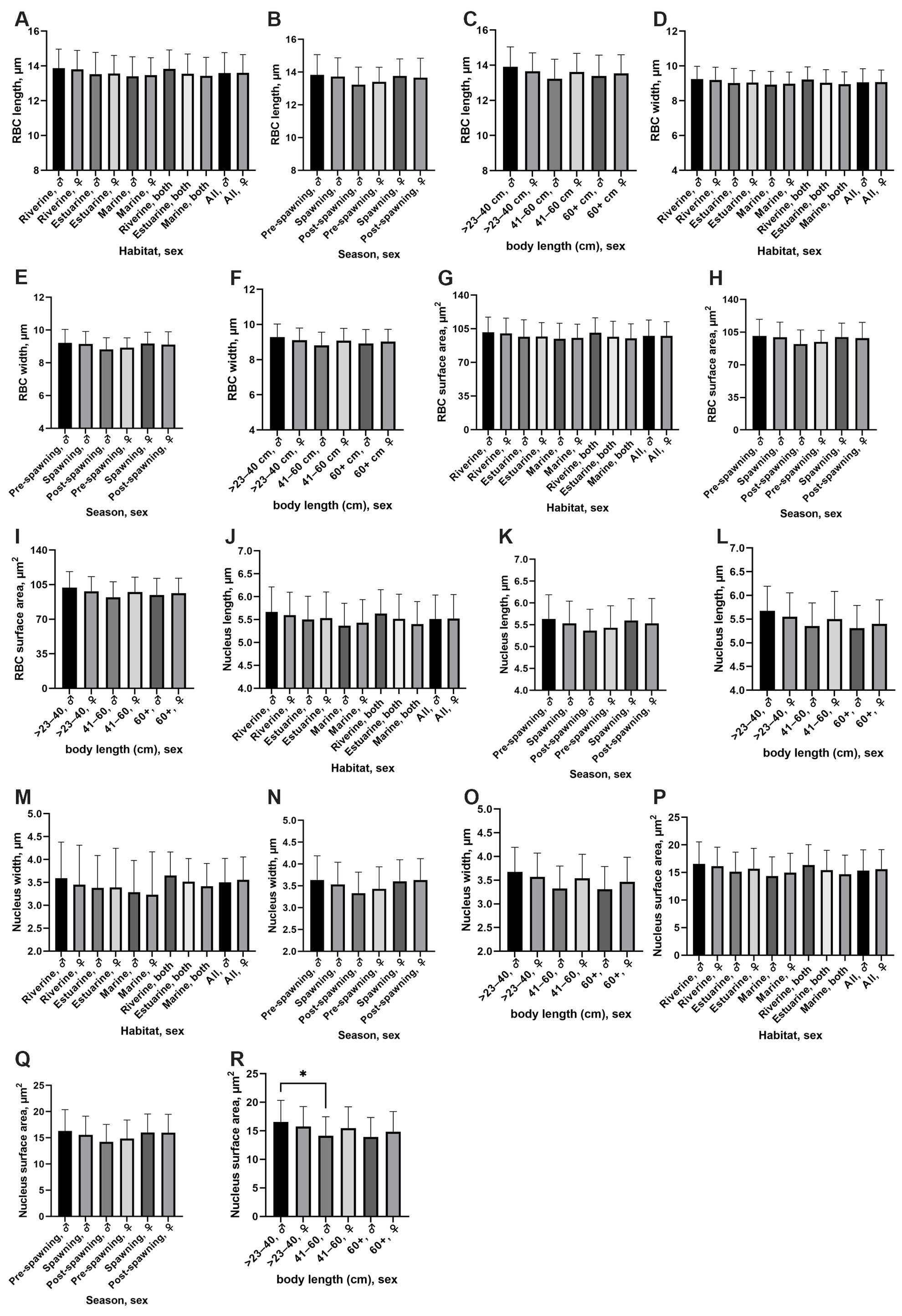

Supplement: S4 Fig — (A–C) RBC lengths across different habitats (A), capture seasons (B), and body lengths (C). (D–F) RBC width across habitats (D), seasons (E), and body lengths (F). (G–I) RBC surface area across different habitats (G), seasons (H), and body lengths (I). (J–L) Nucleus length across different habitats (J), seasons (K), and body lengths (L). (M–O) Nucleus width across habitats (M), seasons (N), and body lengths (O). (P–R) Nucleus surface area across different habitats (P), seasons (Q), and body lengths (R). Statistics, one-way ANOVA with Tukey’s multiple comparisons test; * p < 0.05. Data are represented as mean ± standard deviation. (TIF) [file pone.0320628.s004.tif]

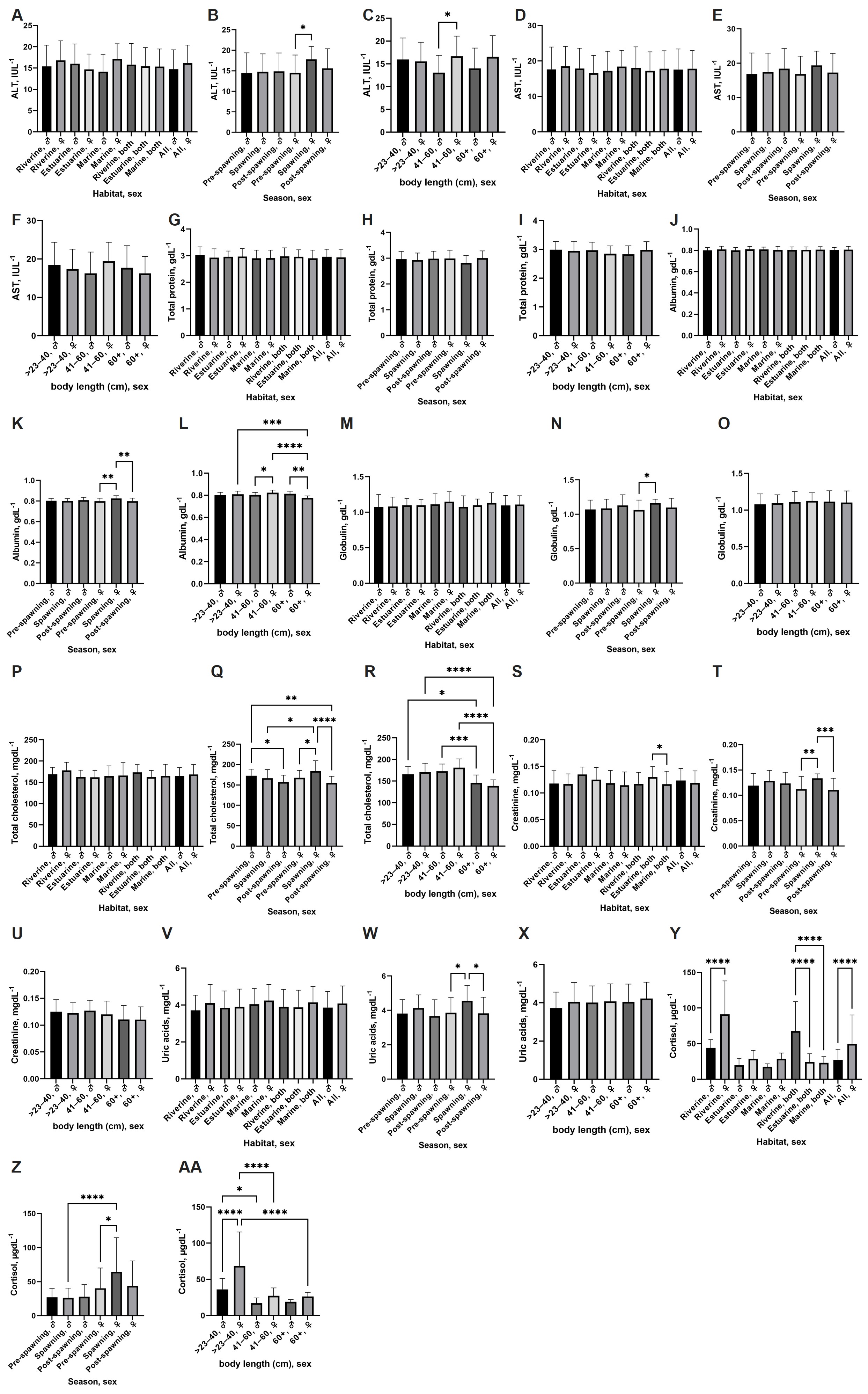

Supplement: S5 Fig — (A–C) ALT levels across habitats (A), capture seasons (B), and body lengths (C). (D–F) AST levels across habitats (D), seasons (E), and body lengths (F). (G–I) Total protein levels across different habitats (G), seasons (H), and body lengths (I). (J–L) Albumin levels across habitats (J), seasons (K), and body lengths (L). (M–O) Globulin levels across habitats (M), seasons (N), and body lengths (O). (P–R) Total cholesterol levels across habitats (P), seasons (Q), and body lengths (R). (S–U) Creatinine levels across habitats (S), seasons (T), and body lengths (U). (V–X) Uric acid levels across habitats (V), seasons (W), and body lengths (X). (Y–AA) Cortisol levels across different habitats (Y), body lengths (Z), and seasons (AA). Statistics, one-way ANOVA with Tukey’s multiple comparisons test; * p < 0.05, ** p < 0.01, *** p < 0.001, **** p < 0.0001. Data are represented as mean ± standard deviation. (JPG) [file pone.0320628.s005.jpg]

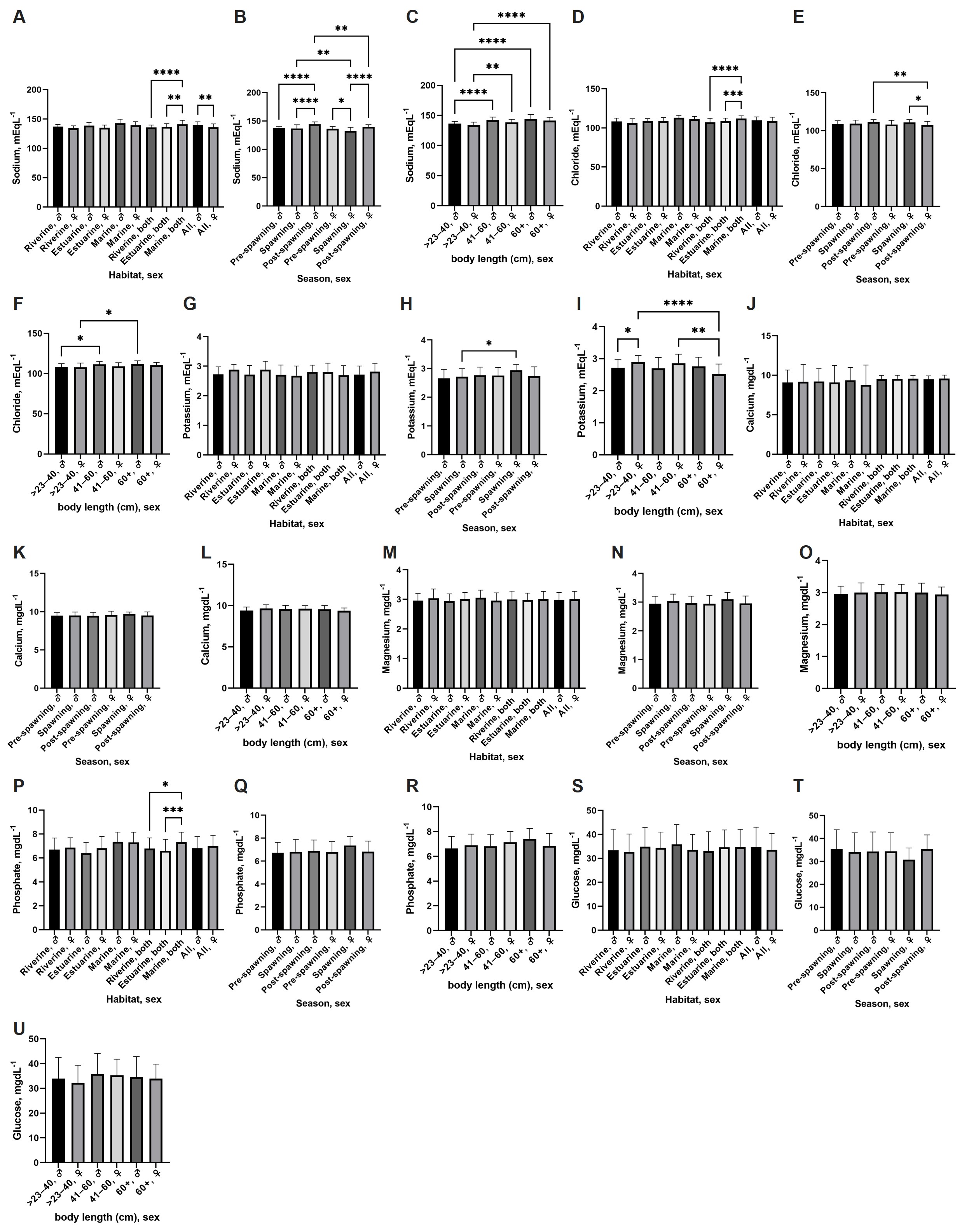

Supplement: S6 Fig — (A–C) Sodium levels across habitats (A), seasons (B), and body lengths (C). (D–F) Chloride levels across habitats (D), seasons (E), and body lengths (F). (G–I) Potassium levels across habitats (G), seasons (H), and body lengths (I). (J–L) Calcium levels across habitats (J), seasons (K), and body lengths (L). (M–O) Magnesium levels across habitats (M), seasons (N), and body lengths (O). (P–R) Phosphate levels across habitats (P), seasons (Q), and body lengths (R). (S) Glucose levels across body lengths and seasons. Statistics, one-way ANOVA with Tukey’s multiple comparisons test; * p < 0.05, ** p < 0.01, *** p < 0.001, **** p < 0.0001. Data are represented as mean ± standard deviation. (JPG) [file pone.0320628.s006.jpg]

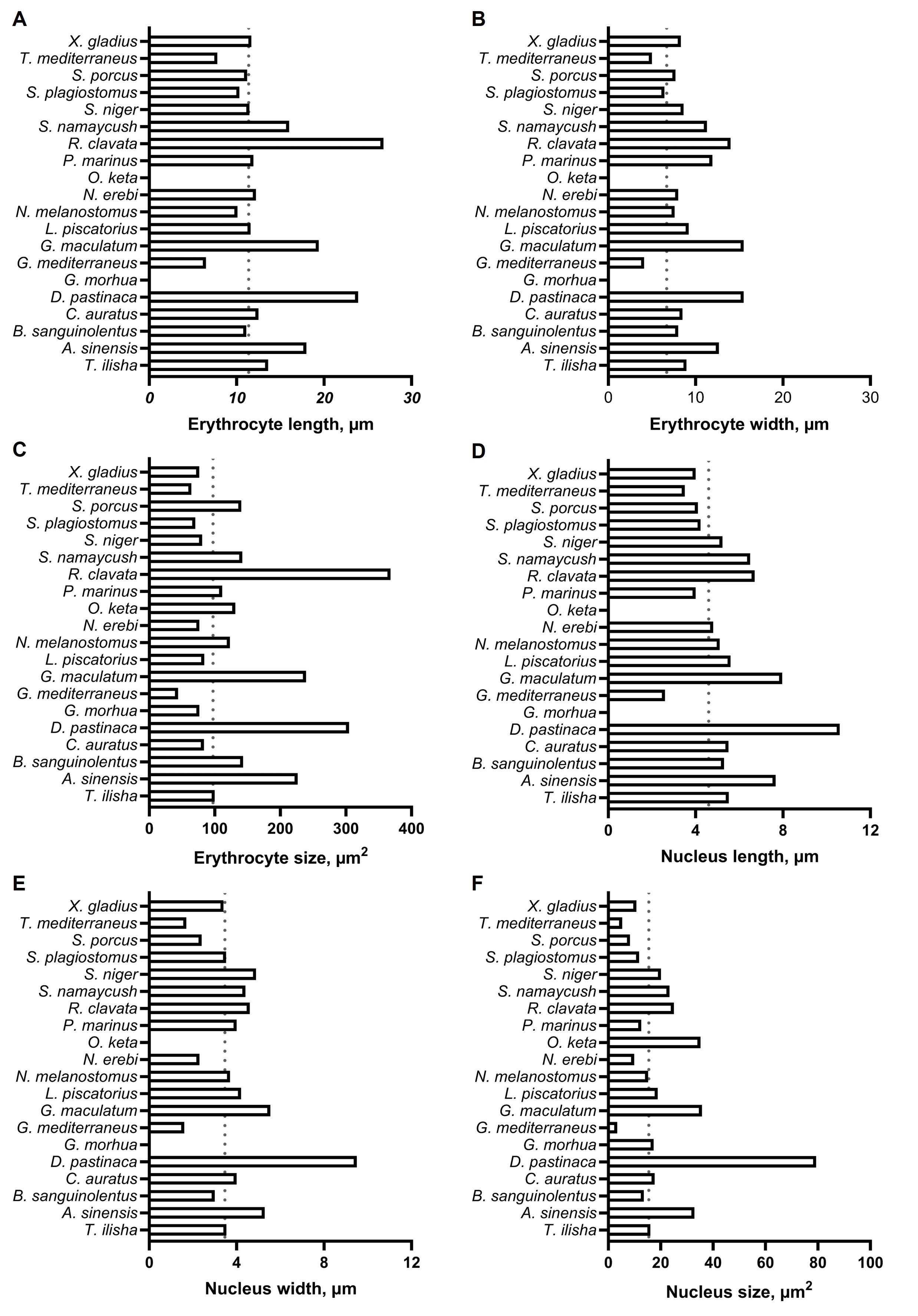

Supplement: S7 Fig — The length (A, D), width (B, E), and size (C, F) of both erythrocytes (A–C) and their nuclei (D–F) are presented. The species examined include Tenualosa ilisha (Hilsa Shad), Acipenser sinensis (Chinese Sturgeon), Blennius sanguinolentus (Black Sea Blenny), Carassius auratus auratus (Gold Crucian Carp, Goldfish), Dasyatis pastinaca (Common Stingray), Gadus morhua (Atlantic Cod), Gaidropsarus mediterraneus (Shore Rockling), Glyptosternon maculatum (Regan, Barkley), Lophius piscatorius (Angler, European Angler), Neogobius melanostomus (Round Goby), Nematalosa erebi (Australian River Gizzard Shad, Bony Bream), Oncorhynchus keta (Chum Salmon), Petromyzon marinus (Sea Lamprey), Raja clavata (Thornback Ray), Salvelinus namaycush (Lake Trout), Schizopyge niger (Alghad Snowtrout), Schizopyge plagiostomus (Snow Trout), Scorpaena porcus (Black Scorpionfish), Trachurus mediterraneus ponticus (Horse Mackerel), and Xiphias gladius (Swordfish, Broadbill). (JPG) [file pone.0320628.s007.jpg]
