# Supplementary material for: Biophysical assessments and blood profiling reveal physiological adaptations and environmental interactions of hilsa shad (Tenualosa ilisha)
Source: PLoS One. 2025 Apr 1;20(4):e0320628. doi: 10.1371/journal.pone.0320628 (PMC11960910; doi:10.1371/journal.pone.0320628)
Supplement: S1 Data — (PDF) [file pone.0320628.s008.pdf]

Figure 1

Panel A

Riverine, ♂	Riverine, ♀	Estuarine, ♂	Estuarine, ♀	Marine, ♂	Marine, ♀
14.51	13.59	13.79	12.85	13.65	12.64
14.60	13.72	14.20	12.99	13.80	12.70
14.71	13.73	14.27	13.13	14.08	12.81
14.73	13.93	14.50	13.17	14.39	12.88
14.75	14.07	14.50	13.23	14.53	12.89
14.90	14.10	14.54	13.28	14.55	13.04
14.90	14.24	14.69	13.29	14.64	13.06
14.90	14.26	14.75	13.34	14.71	13.07
15.06	14.31	14.83	13.36	14.83	13.36
15.08	14.34	14.94	13.41	14.84	13.40
15.11	14.49	15.06	13.49	14.87	13.60
15.19	14.63	15.17	13.57	15.00	13.85
15.35	14.64	15.50	13.58	15.01	13.87
15.42	14.67	15.67	13.62	15.13	14.18
15.45	14.74	15.71	14.06	15.18	14.26
15.62	14.94	15.75	14.34	15.20	14.52
16.02	15.02	15.79	14.46	15.29	14.57
16.14	15.12	16.00	14.66	15.32	14.74
16.16	15.14	16.23	14.73	15.38	15.06
16.25	15.50	16.31	14.85	15.75	15.17
16.26	15.52	16.38	14.86	15.77	15.27
16.27	15.55	16.38	14.94	16.10	15.35
16.42	15.61	16.43	14.98	16.19	15.50
16.59	15.61	16.66	15.12	16.26	15.60
16.60	15.68	16.78	15.15	16.27	15.68
16.73	15.74	16.80	15.24	16.46	15.71
16.79	15.75	16.87	15.36	16.46	15.86
16.82	15.76	16.90	15.58	16.64	15.87
16.88	15.85	16.91	15.64	16.83	15.89
16.97	15.95	16.94	15.91	16.98	15.98

Panel B

Pre-spawning, ♂	Spawning, ♂	Post-spawning, ♂	Pre-spawning, ♀	Spawning, ♀	Post-spawning, ♀
15.11	14.51	16.26	14.49	13.59	15.52
15.19	14.60	16.27	14.63	13.72	15.55
15.35	14.71	16.42	14.64	13.73	15.61
15.42	14.73	16.59	14.67	13.93	15.61
15.45	14.75	16.60	14.74	14.07	15.68
15.62	14.90	16.73	14.94	14.10	15.74
16.02	14.90	16.79	15.02	14.24	15.75
16.14	14.90	16.82	15.12	14.26	15.76
16.16	15.06	16.88	15.14	14.31	15.85
16.25	15.08	16.97	15.50	14.34	15.95
15.06	13.79	16.38	13.49	12.85	14.86
15.17	14.20	16.38	13.57	12.99	14.94
15.50	14.27	16.43	13.58	13.13	14.98
15.67	14.50	16.66	13.62	13.17	15.12
15.71	14.50	16.78	14.06	13.23	15.15
15.75	14.54	16.80	14.34	13.28	15.24
15.79	14.69	16.87	14.46	13.29	15.36
16.00	14.75	16.90	14.66	13.34	15.58
16.23	14.83	16.91	14.73	13.36	15.64
16.31	14.94	16.94	14.85	13.41	15.91
14.87	13.65	15.77	13.60	12.64	15.27
15.00	13.80	16.10	13.85	12.70	15.35
15.01	14.08	16.19	13.87	12.81	15.50
15.13	14.39	16.26	14.18	12.88	15.60
15.18	14.53	16.27	14.26	12.89	15.68
15.20	14.55	16.46	14.52	13.04	15.71
15.29	14.64	16.46	14.57	13.06	15.86
15.32	14.71	16.64	14.74	13.07	15.87
15.38	14.83	16.83	15.06	13.36	15.89
15.75	14.84	16.98	15.17	13.40	15.98

Panel C

>23–40, ♂	>23–40, ♀	41–60, ♂	41–60, ♀	60+, ♂	60+, ♀
14.51	13.59	15.17	13.57	15.13	14.74
14.60	13.72	15.50	13.58	15.18	15.06
14.71	13.73	15.67	13.62	15.20	15.17
14.73	13.93	15.71	14.06	15.29	15.27
14.75	14.07	15.75	14.34	16.10	15.35
14.90	14.10	16.38	14.94	16.19	15.50
14.90	14.24	16.43	14.98	16.26	15.60
14.90	14.26	16.66	15.12	16.27	15.68
15.06	14.31	16.78	15.15	16.46	15.71
15.08	14.34	16.80	15.24	16.46	15.86
15.11	14.49	16.87	15.36	16.64	15.87
15.19	14.63	16.90	15.58	16.83	15.89

15.35	14.64	16.91	15.64	16.98	15.98
15.42	14.67	16.94	15.91		15.17
15.45	14.74	13.65	12.64		15.27
15.62	14.94	13.80	12.70		
16.02	15.02	14.08	12.81		
16.14	15.12	14.39	12.88		
16.16	15.14	14.53	12.89		
16.25	15.50	14.55	13.04		
16.26	15.52	14.64	13.06		
16.27	15.55	14.71	13.07		
16.42	15.61	14.83	13.36		
16.59	15.61	14.84	13.40		
16.60	15.68	14.87	13.60		
16.73	15.74	15.00	13.85		
16.79	15.75	15.01			
16.82	15.76	15.32			
16.88	15.85	15.38			
16.97	15.95	15.75			
13.79	12.85	15.77			
14.20	12.99				
14.27	13.13				
14.50	13.17				
14.50	13.23				
14.54	13.28				
14.69	13.29				
14.75	13.34				
14.83	13.36				
14.94	13.41				
15.06	13.49				
15.79	14.46				
16.00	14.66				
16.23	14.73				
16.31	14.85				
16.38	14.86				
	13.87				
	14.74				
	15.06				

Panel D

Riverine, ♂	Riverine, ♀	Estuarine, ♂	Estuarine, ♀	Marine, ♂	Marine, ♀
2.61	2.45	2.48	2.31	2.46	2.28
2.63	2.47	2.56	2.34	2.48	2.29
2.65	2.47	2.57	2.36	2.53	2.31
2.65	2.51	2.61	2.37	2.59	2.32
2.65	2.53	2.61	2.38	2.62	2.32
2.68	2.54	2.62	2.39	2.62	2.35
2.68	2.56	2.64	2.39	2.63	2.35

2.68	2.57	2.65	2.40	2.65	2.35
2.71	2.58	2.67	2.40	2.67	2.40
2.71	2.58	2.69	2.41	2.67	2.41
2.72	2.61	2.71	2.43	2.68	2.45
2.73	2.63	2.73	2.44	2.70	2.49
2.76	2.63	2.79	2.44	2.70	2.50
2.78	2.64	2.82	2.45	2.72	2.55
2.78	2.65	2.83	2.53	2.73	2.57
2.81	2.69	2.83	2.58	2.74	2.61
2.88	2.70	2.84	2.60	2.75	2.62
2.90	2.72	2.88	2.64	2.76	2.65
2.91	2.72	2.92	2.65	2.77	2.71
2.92	2.79	2.94	2.67	2.83	2.73
2.93	2.79	2.95	2.67	2.84	2.75
2.93	2.80	2.95	2.69	2.90	2.76
2.96	2.81	2.96	2.70	2.91	2.79
2.99	2.81	3.00	2.72	2.93	2.81
2.99	2.82	3.02	2.73	2.93	2.82
3.01	2.83	3.02	2.74	2.96	2.83
3.02	2.83	3.04	2.76	2.96	2.85
3.03	2.84	3.04	2.80	2.99	2.86
3.04	2.85	3.04	2.81	3.03	2.86
3.05	2.87	3.05	2.86	3.06	2.88

Panel E

Pre-spawning, ♂	Spawning, ♂	Post-spawning, ♂	Pre-spawning, ♀	Spawning, ♀	Post-spawning, ♀
2.72	2.61	2.93	2.61	2.45	2.79
2.73	2.63	2.93	2.63	2.47	2.80
2.76	2.65	2.96	2.63	2.47	2.81
2.78	2.65	2.99	2.64	2.51	2.81
2.78	2.65	2.99	2.65	2.53	2.82
2.81	2.68	3.01	2.69	2.54	2.83
2.88	2.68	3.02	2.70	2.56	2.83
2.90	2.68	3.03	2.72	2.57	2.84

2.91	2.71	3.04	2.72	2.58	2.85
2.92	2.71	3.05	2.79	2.58	2.87
2.71	2.48	2.95	2.43	2.31	2.67
2.73	2.56	2.95	2.44	2.34	2.69
2.79	2.57	2.96	2.44	2.36	2.70
2.82	2.61	3.00	2.45	2.37	2.72
2.83	2.61	3.02	2.53	2.38	2.73
2.83	2.62	3.02	2.58	2.39	2.74
2.84	2.64	3.04	2.60	2.39	2.76
2.88	2.65	3.04	2.64	2.40	2.80
2.92	2.67	3.04	2.65	2.40	2.81
2.94	2.69	3.05	2.67	2.41	2.86
2.68	2.46	2.84	2.45	2.28	2.75
2.70	2.48	2.90	2.49	2.29	2.76
2.70	2.53	2.91	2.50	2.31	2.79
2.72	2.59	2.93	2.55	2.32	2.81
2.73	2.62	2.93	2.57	2.32	2.82
2.74	2.62	2.96	2.61	2.35	2.83
2.75	2.63	2.96	2.62	2.35	2.85
2.76	2.65	2.99	2.65	2.35	2.86
2.77	2.67	3.03	2.71	2.40	2.86
2.83	2.67	3.06	2.73	2.41	2.88

Panel F

>23–40, ♂	>23–40, ♀	41–60, ♂	41–60, ♀	60+, ♂	60+, ♀
2.61	2.45	2.73	2.44	2.72	2.65
2.63	2.47	2.79	2.44	2.73	2.71
2.65	2.47	2.82	2.45	2.74	2.73
2.65	2.51	2.83	2.53	2.75	2.75
2.65	2.53	2.83	2.58	2.90	2.76
2.68	2.54	2.95	2.69	2.91	2.79
2.68	2.56	2.96	2.70	2.93	2.81
2.68	2.57	3.00	2.72	2.93	2.82
2.71	2.58	3.02	2.73	2.96	2.83
2.71	2.58	3.02	2.74	2.96	2.85
2.72	2.61	3.04	2.76	2.99	2.86
2.73	2.63	3.04	2.80	3.03	2.86
2.76	2.63	3.04	2.81	3.06	2.88
2.78	2.64	3.05	2.86		2.73
2.78	2.65	2.46	2.28		2.75
2.81	2.69	2.48	2.29		
2.88	2.70	2.53	2.31		
2.90	2.72	2.59	2.32		
2.91	2.72	2.62	2.32		
2.92	2.79	2.62	2.35		
2.93	2.79	2.63	2.35		
2.93	2.80	2.65	2.35		

2.96	2.81	2.67	2.40		
2.99	2.81	2.67	2.41		
2.99	2.82	2.68	2.45		
3.01	2.83	2.70	2.49		
3.02	2.83	2.70			
3.03	2.84	2.76			
3.04	2.85	2.77			
3.05	2.87	2.83			
2.48	2.31	2.84			
2.56	2.34				
2.57	2.36				
2.61	2.37				
2.61	2.38				
2.62	2.39				
2.64	2.39				
2.65	2.40				
2.67	2.40				
2.69	2.41				
2.71	2.43				
2.84	2.60				
2.88	2.64				
2.92	2.65				
2.94	2.67				
2.95	2.67				
	2.50				
	2.65				
	2.71				

Panel G

Riverine, ♂	Riverine, ♀	Estuarine, ♂	Estuarine, ♀	Marine, ♂	Marine, ♀
43.53	42.13	42.06	39.71	39.59	37.29
43.80	42.53	43.31	40.14	40.02	37.47
44.13	42.56	43.52	40.57	40.83	37.79
44.19	43.18	44.23	40.70	41.73	38.00
44.25	43.62	44.23	40.88	42.14	38.03
44.70	43.71	44.35	41.04	42.20	38.47
44.70	44.14	44.80	41.07	42.46	38.53
44.70	44.21	44.99	41.22	42.66	38.56
45.18	44.36	45.23	41.28	43.01	39.41
45.24	44.45	45.57	41.44	43.04	39.53
45.33	44.92	45.93	41.68	43.12	40.12
45.57	45.35	46.27	41.93	43.50	40.86
46.05	45.38	47.28	41.96	43.53	40.92
46.26	45.48	47.79	42.09	43.88	41.83
46.35	45.69	47.92	43.45	44.02	42.07
46.86	46.31	48.04	44.31	44.08	42.83
48.06	46.56	48.16	44.68	44.34	42.98

48.42	46.87	48.80	45.30	44.43	43.48
48.48	46.93	49.50	45.52	44.60	44.43
48.75	48.05	49.75	45.89	45.68	44.75
48.78	48.11	49.96	45.92	45.73	45.05
48.81	48.21	49.96	46.16	46.69	45.28
49.26	48.39	50.11	46.29	46.95	45.73
49.77	48.39	50.81	46.72	47.15	46.02
49.80	48.61	51.18	46.81	47.18	46.26
50.19	48.79	51.24	47.09	47.73	46.34
50.37	48.83	51.45	47.46	47.73	46.79
50.46	48.86	51.55	48.14	48.26	46.82
50.64	49.14	51.58	48.33	48.81	46.88
50.91	49.45	51.67	49.16	49.24	47.14

Panel H

Pre-spawning, ♂	Spawning, ♂	Post-spawning, ♂	Pre-spawning, ♀	Spawning, ♀	Post-spawning, ♀
45.33	43.53	48.78	44.92	42.13	48.11
45.57	43.80	48.81	45.35	42.53	48.21
46.05	44.13	49.26	45.38	42.56	48.39
46.26	44.19	49.77	45.48	43.18	48.39
46.35	44.25	49.80	45.69	43.62	48.61
46.86	44.70	50.19	46.31	43.71	48.79
48.06	44.70	50.37	46.56	44.14	48.83
48.42	44.70	50.46	46.87	44.21	48.86
48.48	45.18	50.64	46.93	44.36	49.14
48.75	45.24	50.91	48.05	44.45	49.45
45.93	42.06	49.96	41.68	39.71	45.92
46.27	43.31	49.96	41.93	40.14	46.16
47.28	43.52	50.11	41.96	40.57	46.29
47.79	44.23	50.81	42.09	40.70	46.72
47.92	44.23	51.18	43.45	40.88	46.81
48.04	44.35	51.24	44.31	41.04	47.09

48.16	44.80	51.45	44.68	41.07	47.46
48.80	44.99	51.55	45.30	41.22	48.14
49.50	45.23	51.58	45.52	41.28	48.33
49.75	45.57	51.67	45.89	41.44	49.16
43.12	39.59	45.73	40.12	37.29	45.05
43.50	40.02	46.69	40.86	37.47	45.28
43.53	40.83	46.95	40.92	37.79	45.73
43.88	41.73	47.15	41.83	38.00	46.02
44.02	42.14	47.18	42.07	38.03	46.26
44.08	42.20	47.73	42.83	38.47	46.34
44.34	42.46	47.73	42.98	38.53	46.79
44.43	42.66	48.26	43.48	38.56	46.82
44.60	43.01	48.81	44.43	39.41	46.88
45.68	43.04	49.24	44.75	39.53	47.14

Panel I

>23–40, ♂	>23–40, ♀	41–60, ♂	41–60, ♀	60+, ♂	60+, ♀
43.53	42.13	46.27	41.93	43.88	43.48
43.80	42.53	47.28	41.96	44.02	44.43
44.13	42.56	47.79	42.09	44.08	44.75
44.19	43.18	47.92	43.45	44.34	45.05
44.25	43.62	48.04	44.31	46.69	45.28
44.70	43.71	49.96	46.16	46.95	45.73
44.70	44.14	50.11	46.29	47.15	46.02
44.70	44.21	50.81	46.72	47.18	46.26
45.18	44.36	51.18	46.81	47.73	46.34
45.24	44.45	51.24	47.09	47.73	46.79
45.33	44.92	51.45	47.46	48.26	46.82
45.57	45.35	51.55	48.14	48.81	46.88
46.05	45.38	51.58	48.33	49.24	47.14
46.26	45.48	51.67	49.16		44.75
46.35	45.69	39.59	37.29		45.05
46.86	46.31	40.02	37.47		
48.06	46.56	40.83	37.79		
48.42	46.87	41.73	38.00		
48.48	46.93	42.14	38.03		
48.75	48.05	42.20	38.47		
48.78	48.11	42.46	38.53		
48.81	48.21	42.66	38.56		
49.26	48.39	43.01	39.41		
49.77	48.39	43.04	39.53		
49.80	48.61	43.12	40.12		
50.19	48.79	43.50	40.86		
50.37	48.83	43.53			
50.46	48.86	44.43			
50.64	49.14	44.60			
50.91	49.45	45.68			

42.06	39.71	45.73			
43.31	40.14				
43.52	40.57				
44.23	40.70				
44.23	40.88				
44.35	41.04				
44.80	41.07				
44.99	41.22				
45.23	41.28				
45.57	41.44				
45.93	41.68				
48.16	44.68				
48.80	45.30				
49.50	45.52				
49.75	45.89				
49.96	45.92				
	40.92				
	43.48				
	44.43				

Riverine, both	Estuarine, both	Marine, both	All, ♂	All, ♀
14.51	13.79	13.65	14.51	13.59
14.60	14.20	13.80	14.60	13.72
14.71	14.27	14.08	14.71	13.73
14.73	14.50	14.39	14.73	13.93
14.75	14.50	14.53	14.75	14.07
14.90	14.54	14.55	14.90	14.10
14.90	14.69	14.64	14.90	14.24
14.90	14.75	14.71	14.90	14.26
15.06	14.83	14.83	15.06	14.31
15.08	14.94	14.84	15.08	14.34
15.11	15.06	14.87	15.11	14.49
15.19	15.17	15.00	15.19	14.63
15.35	15.50	15.01	15.35	14.64
15.42	15.67	15.13	15.42	14.67
15.45	15.71	15.18	15.45	14.74
15.62	15.75	15.20	15.62	14.94
16.02	15.79	15.29	16.02	15.02
16.14	16.00	15.32	16.14	15.12
16.16	16.23	15.38	16.16	15.14
16.25	16.31	15.75	16.25	15.50
16.26	16.38	15.77	16.26	15.52
16.27	16.38	16.10	16.27	15.55
16.42	16.43	16.19	16.42	15.61
16.59	16.66	16.26	16.59	15.61
16.60	16.78	16.27	16.60	15.68
16.73	16.80	16.46	16.73	15.74
16.79	16.87	16.46	16.79	15.75
16.82	16.90	16.64	16.82	15.76
16.88	16.91	16.83	16.88	15.85
16.97	16.94	16.98	16.97	15.95
13.59	12.85	12.64	13.79	12.85
13.72	12.99	12.70	14.20	12.99
13.73	13.13	12.81	14.27	13.13
13.93	13.17	12.88	14.50	13.17
14.07	13.23	12.89	14.50	13.23
14.10	13.28	13.04	14.54	13.28
14.24	13.29	13.06	14.69	13.29
14.26	13.34	13.07	14.75	13.34
14.31	13.36	13.36	14.83	13.36
14.34	13.41	13.40	14.94	13.41
14.49	13.49	13.60	15.06	13.49
14.63	13.57	13.85	15.17	13.57
14.64	13.58	13.87	15.50	13.58
14.67	13.62	14.18	15.67	13.62

14.74	14.06	14.26	15.71	14.06
14.94	14.34	14.52	15.75	14.34
15.02	14.46	14.57	15.79	14.46
15.12	14.66	14.74	16.00	14.66
15.14	14.73	15.06	16.23	14.73
15.50	14.85	15.17	16.31	14.85
15.52	14.86	15.27	16.38	14.86
15.55	14.94	15.35	16.38	14.94
15.61	14.98	15.50	16.43	14.98
15.61	15.12	15.60	16.66	15.12
15.68	15.15	15.68	16.78	15.15
15.74	15.24	15.71	16.80	15.24
15.75	15.36	15.86	16.87	15.36
15.76	15.58	15.87	16.90	15.58
15.85	15.64	15.89	16.91	15.64
15.95	15.91	15.98	16.94	15.91
			13.65	12.64
			13.80	12.70
			14.08	12.81
			14.39	12.88
			14.53	12.89
			14.55	13.04
			14.64	13.06
			14.71	13.07
			14.83	13.36
			14.84	13.40
			14.87	13.60
			15.00	13.85
			15.01	13.87
			15.13	14.18
			15.18	14.26
			15.20	14.52
			15.29	14.57
			15.32	14.74
			15.38	15.06
			15.75	15.17
			15.77	15.27
			16.10	15.35
			16.19	15.50
			16.26	15.60
			16.27	15.68
			16.46	15.71
			16.46	15.86
			16.64	15.87
			16.83	15.89
			16.98	15.98

Riverine, both	Estuarine, both	Marine, both	All, ♂	All, ♀
2.61	2.48	2.46	2.61	2.45
2.63	2.56	2.48	2.63	2.47
2.65	2.57	2.53	2.65	2.47
2.65	2.61	2.59	2.65	2.51
2.65	2.61	2.62	2.65	2.53
2.68	2.62	2.62	2.68	2.54
2.68	2.64	2.63	2.68	2.56

2.68	2.65	2.65	2.68	2.57
2.71	2.67	2.67	2.71	2.58
2.71	2.69	2.67	2.71	2.58
2.72	2.71	2.68	2.72	2.61
2.73	2.73	2.70	2.73	2.63
2.76	2.79	2.70	2.76	2.63
2.78	2.82	2.72	2.78	2.64
2.78	2.83	2.73	2.78	2.65
2.81	2.83	2.74	2.81	2.69
2.88	2.84	2.75	2.88	2.70
2.90	2.88	2.76	2.90	2.72
2.91	2.92	2.77	2.91	2.72
2.92	2.94	2.83	2.92	2.79
2.93	2.95	2.84	2.93	2.79
2.93	2.95	2.90	2.93	2.80
2.96	2.96	2.91	2.96	2.81
2.99	3.00	2.93	2.99	2.81
2.99	3.02	2.93	2.99	2.82
3.01	3.02	2.96	3.01	2.83
3.02	3.04	2.96	3.02	2.83
3.03	3.04	2.99	3.03	2.84
3.04	3.04	3.03	3.04	2.85
3.05	3.05	3.06	3.05	2.87
2.45	2.31	2.28	2.48	2.31
2.47	2.34	2.29	2.56	2.34
2.47	2.36	2.31	2.57	2.36
2.51	2.37	2.32	2.61	2.37
2.53	2.38	2.32	2.61	2.38
2.54	2.39	2.35	2.62	2.39
2.56	2.39	2.35	2.64	2.39
2.57	2.40	2.35	2.65	2.40
2.58	2.40	2.40	2.67	2.40
2.58	2.41	2.41	2.69	2.41
2.61	2.43	2.45	2.71	2.43
2.63	2.44	2.49	2.73	2.44
2.63	2.44	2.50	2.79	2.44
2.64	2.45	2.55	2.82	2.45
2.65	2.53	2.57	2.83	2.53
2.69	2.58	2.61	2.83	2.58
2.70	2.60	2.62	2.84	2.60
2.72	2.64	2.65	2.88	2.64
2.72	2.65	2.71	2.92	2.65
2.79	2.67	2.73	2.94	2.67
2.79	2.67	2.75	2.95	2.67
2.80	2.69	2.76	2.95	2.69
2.81	2.70	2.79	2.96	2.70
2.81	2.72	2.81	3.00	2.72

2.82	2.73	2.82	3.02	2.73
2.83	2.74	2.83	3.02	2.74
2.83	2.76	2.85	3.04	2.76
2.84	2.80	2.86	3.04	2.80
2.85	2.81	2.86	3.04	2.81
2.87	2.86	2.88	3.05	2.86
			2.46	2.28
			2.48	2.29
			2.53	2.31
			2.59	2.32
			2.62	2.32
			2.62	2.35
			2.63	2.35
			2.65	2.35
			2.67	2.40
			2.67	2.41
			2.68	2.45
			2.70	2.49
			2.70	2.50
			2.72	2.55
			2.73	2.57
			2.74	2.61
			2.75	2.62
			2.76	2.65
			2.77	2.71
			2.83	2.73
			2.84	2.75
			2.90	2.76
			2.91	2.79
			2.93	2.81
			2.93	2.82
			2.96	2.83
			2.96	2.85
			2.99	2.86
			3.03	2.86
			3.06	2.88

Riverine, both	Estuarine, both	Marine, both	All, ♂	All, ♀
43.53	42.06	39.59	43.53	42.13
43.80	43.31	40.02	43.80	42.53
44.13	43.52	40.83	44.13	42.56
44.19	44.23	41.73	44.19	43.18
44.25	44.23	42.14	44.25	43.62
44.70	44.35	42.20	44.70	43.71
44.70	44.80	42.46	44.70	44.14
44.70	44.99	42.66	44.70	44.21
45.18	45.23	43.01	45.18	44.36
45.24	45.57	43.04	45.24	44.45
45.33	45.93	43.12	45.33	44.92
45.57	46.27	43.50	45.57	45.35
46.05	47.28	43.53	46.05	45.38
46.26	47.79	43.88	46.26	45.48
46.35	47.92	44.02	46.35	45.69
46.86	48.04	44.08	46.86	46.31
48.06	48.16	44.34	48.06	46.56

48.42	48.80	44.43	48.42	46.87
48.48	49.50	44.60	48.48	46.93
48.75	49.75	45.68	48.75	48.05
48.78	49.96	45.73	48.78	48.11
48.81	49.96	46.69	48.81	48.21
49.26	50.11	46.95	49.26	48.39
49.77	50.81	47.15	49.77	48.39
49.80	51.18	47.18	49.80	48.61
50.19	51.24	47.73	50.19	48.79
50.37	51.45	47.73	50.37	48.83
50.46	51.55	48.26	50.46	48.86
50.64	51.58	48.81	50.64	49.14
50.91	51.67	49.24	50.91	49.45
42.13	39.71	37.29	42.06	39.71
42.53	40.14	37.47	43.31	40.14
42.56	40.57	37.79	43.52	40.57
43.18	40.70	38.00	44.23	40.70
43.62	40.88	38.03	44.23	40.88
43.71	41.04	38.47	44.35	41.04
44.14	41.07	38.53	44.80	41.07
44.21	41.22	38.56	44.99	41.22
44.36	41.28	39.41	45.23	41.28
44.45	41.44	39.53	45.57	41.44
44.92	41.68	40.12	45.93	41.68
45.35	41.93	40.86	46.27	41.93
45.38	41.96	40.92	47.28	41.96
45.48	42.09	41.83	47.79	42.09
45.69	43.45	42.07	47.92	43.45
46.31	44.31	42.83	48.04	44.31
46.56	44.68	42.98	48.16	44.68
46.87	45.30	43.48	48.80	45.30
46.93	45.52	44.43	49.50	45.52
48.05	45.89	44.75	49.75	45.89
48.11	45.92	45.05	49.96	45.92
48.21	46.16	45.28	49.96	46.16
48.39	46.29	45.73	50.11	46.29
48.39	46.72	46.02	50.81	46.72
48.61	46.81	46.26	51.18	46.81
48.79	47.09	46.34	51.24	47.09
48.83	47.46	46.79	51.45	47.46
48.86	48.14	46.82	51.55	48.14
49.14	48.33	46.88	51.58	48.33
49.45	49.16	47.14	51.67	49.16
			39.59	37.29
			40.02	37.47
			40.83	37.79
			41.73	38.00

			42.14	38.03
			42.20	38.47
			42.46	38.53
			42.66	38.56
			43.01	39.41
			43.04	39.53
			43.12	40.12
			43.50	40.86
			43.53	40.92
			43.88	41.83
			44.02	42.07
			44.08	42.83
			44.34	42.98
			44.43	43.48
			44.60	44.43
			45.68	44.75
			45.73	45.05
			46.69	45.28
			46.95	45.73
			47.15	46.02
			47.18	46.26
			47.73	46.34
			47.73	46.79
			48.26	46.82
			48.81	46.88
			49.24	47.14
			3.03	2.86
			3.06	2.88

Figure 2

Panel A

Riverine, ♂	Riverine, ♀	Estuarine, ♂	Estuarine, ♀	Marine, ♂	Marine, ♀
4.36	4.08	4.14	3.86	4.10	3.80
4.47	4.20	4.34	3.97	4.22	3.89
4.55	4.25	4.42	4.06	4.36	3.97
4.36	4.08	4.14	3.86	4.10	3.80
4.47	4.20	4.34	3.97	4.22	3.89
4.55	4.25	4.42	4.06	4.36	3.97
4.43	4.19	4.36	3.96	4.33	3.87
4.51	4.31	4.44	4.05	4.45	3.94
4.61	4.37	4.50	4.11	4.50	4.04
4.48	4.28	4.42	3.99	4.40	3.93
4.56	4.36	4.51	4.08	4.50	4.00
4.66	4.43	4.59	4.14	4.59	4.14
4.53	4.31	4.49	4.03	4.46	4.03
4.75	4.53	4.73	4.23	4.67	4.26
4.80	4.65	4.80	4.32	4.74	4.41
4.74	4.50	4.78	4.18	4.63	4.27
4.73	4.53	4.81	4.22	4.65	4.39
4.82	4.58	4.90	4.38	4.74	4.44
4.79	4.61	4.83	4.43	4.67	4.49
5.03	4.70	4.95	4.52	4.80	4.56
5.10	4.80	5.05	4.66	4.84	4.69
4.98	4.65	5.00	4.53	4.75	4.63
5.07	4.87	5.09	4.67	4.92	4.76
5.29	5.00	5.33	4.80	5.14	4.93
5.09	4.93	5.12	4.75	5.04	4.87
5.19	4.89	5.20	4.70	5.12	4.86
5.25	5.01	5.27	4.86	5.15	5.00
5.25	4.91	5.30	4.75	5.15	4.91
5.23	4.99	5.25	4.84	5.15	4.98
5.30	4.93	5.33	4.82	5.21	4.97
5.26	5.00	5.28	4.94	5.20	5.03
5.33	4.96	5.34	4.90	5.32	4.98
5.30	5.05	5.29	5.04	5.30	5.06

Panel B

Pre-spawning, ♂	Spawning, ♂	Post-spawning, ♂	Pre-spawning, ♀	Spawning, ♀	Post-spawning, ♀
4.75	4.36	5.29	4.53	4.08	5.00
4.80	4.47	5.09	4.65	4.20	4.93
4.74	4.55	5.19	4.50	4.25	4.89
4.73	4.43	5.25	4.53	4.19	5.01
4.82	4.51	5.25	4.58	4.31	4.91
4.79	4.61	5.23	4.61	4.37	4.99
5.03	4.48	5.30	4.70	4.28	4.93
5.10	4.56	5.26	4.80	4.36	5.00
4.98	4.66	5.33	4.65	4.43	4.96
5.07	4.53	5.30	4.87	4.31	5.05
4.73	4.14	5.33	4.23	3.86	4.80
4.80	4.34	5.12	4.32	3.97	4.75
4.78	4.42	5.20	4.18	4.06	4.70
4.81	4.36	5.27	4.22	3.96	4.86
4.90	4.44	5.30	4.38	4.05	4.75
4.83	4.50	5.25	4.43	4.11	4.84
4.95	4.42	5.33	4.52	3.99	4.82
5.05	4.51	5.28	4.66	4.08	4.94
5.00	4.59	5.34	4.53	4.14	4.90
5.09	4.49	5.29	4.67	4.03	5.04
4.67	4.10	5.14	4.26	3.80	4.93
4.74	4.22	5.04	4.41	3.89	4.87
4.63	4.36	5.12	4.27	3.97	4.86
4.65	4.33	5.15	4.39	3.87	5.00
4.74	4.45	5.15	4.44	3.94	4.91
4.67	4.50	5.15	4.49	4.04	4.98
4.80	4.40	5.21	4.56	3.93	4.97
4.84	4.50	5.20	4.69	4.00	5.03
4.75	4.59	5.32	4.63	4.14	4.98
4.92	4.46	5.30	4.76	4.03	5.06

Panel C

>23–40, ♂	>23–40, ♀	41–60, ♂	41–60, ♀	60+, ♂	60+, ♀
4.36	4.08	4.80	4.32	4.65	4.69
4.47	4.20	4.78	4.18	4.74	4.63
4.55	4.25	4.81	4.22	4.67	4.76
4.43	4.19	4.90	4.38	4.80	4.93
4.51	4.31	4.83	4.43	5.04	4.87
4.61	4.37	5.12	4.75	5.12	4.86
4.48	4.28	5.20	4.70	5.15	5.00
4.56	4.36	5.27	4.86	5.15	4.91
4.66	4.43	5.30	4.75	5.15	4.98

4.53	4.31	5.25	4.84	5.21	4.97
4.75	4.53	5.33	4.82	5.20	5.03
4.80	4.65	5.28	4.94	5.32	4.98
4.74	4.50	5.34	4.90	5.30	5.06
4.73	4.53	5.29	5.04		4.76
4.82	4.58	4.10	3.80		4.93
4.79	4.61	4.22	3.89		
5.03	4.70	4.36	3.97		
5.10	4.80	4.33	3.87		
4.98	4.65	4.45	3.94		
5.07	4.87	4.50	4.04		
5.29	5.00	4.40	3.93		
5.09	4.93	4.50	4.00		
5.19	4.89	4.59	4.14		
5.25	5.01	4.46	4.03		
5.25	4.91	4.67	4.26		
5.23	4.99	4.74	4.41		
5.30	4.93	4.63			
5.26	5.00	4.84			
5.33	4.96	4.75			
5.30	5.05	4.92			
4.14	3.86	5.14			
4.34	3.97				
4.42	4.06				
4.36	3.96				
4.44	4.05				
4.50	4.11				
4.42	3.99				
4.51	4.08				
4.59	4.14				
4.49	4.03				
4.73	4.23				
4.95	4.52				
5.05	4.66				
5.00	4.53				
5.09	4.67				
5.33	4.80				
	4.27				
	4.69				
	4.63				

Riverine, both	Estuarine, both	Marine, both	All, ♂	All, ♀
4.36	4.14	4.10	4.36	4.08
4.47	4.34	4.22	4.47	4.20
4.55	4.42	4.36	4.55	4.25
4.36	4.14	4.10	4.36	4.08
4.47	4.34	4.22	4.47	4.20
4.55	4.42	4.36	4.55	4.25
4.43	4.36	4.33	4.43	4.19
4.51	4.44	4.45	4.51	4.31
4.61	4.50	4.50	4.61	4.37
4.48	4.42	4.40	4.48	4.28
4.56	4.51	4.50	4.56	4.36
4.66	4.59	4.59	4.66	4.43
4.53	4.49	4.46	4.53	4.31
4.75	4.73	4.67	4.75	4.53
4.80	4.80	4.74	4.80	4.65
4.74	4.78	4.63	4.74	4.50
4.73	4.81	4.65	4.73	4.53
4.82	4.90	4.74	4.82	4.58
4.79	4.83	4.67	4.79	4.61
5.03	4.95	4.80	5.03	4.70
5.10	5.05	4.84	5.10	4.80
4.98	5.00	4.75	4.98	4.65
5.07	5.09	4.92	5.07	4.87
5.29	5.33	5.14	5.29	5.00
5.09	5.12	5.04	5.09	4.93
5.19	5.20	5.12	5.19	4.89
5.25	5.27	5.15	5.25	5.01
5.25	5.30	5.15	5.25	4.91
5.23	5.25	5.15	5.23	4.99
5.30	5.33	5.21	5.30	4.93
5.26	5.28	5.20	5.26	5.00
5.33	5.34	5.32	5.33	4.96
5.30	5.29	5.30	5.30	5.05
4.08	3.86	3.80	4.14	3.86
4.20	3.97	3.89	4.34	3.97
4.25	4.06	3.97	4.42	4.06
4.19	3.96	3.87	4.36	3.96
4.31	4.05	3.94	4.44	4.05
4.37	4.11	4.04	4.50	4.11
4.28	3.99	3.93	4.42	3.99
4.36	4.08	4.00	4.51	4.08
4.43	4.14	4.14	4.59	4.14
4.31	4.03	4.03	4.49	4.03
4.53	4.23	4.26	4.73	4.23

4.65	4.32	4.41	4.80	4.32
4.50	4.18	4.27	4.78	4.18
4.53	4.22	4.39	4.81	4.22
4.58	4.38	4.44	4.90	4.38
4.61	4.43	4.49	4.83	4.43
4.70	4.52	4.56	4.95	4.52
4.80	4.66	4.69	5.05	4.66
4.65	4.53	4.63	5.00	4.53
4.87	4.67	4.76	5.09	4.67
5.00	4.80	4.93	5.33	4.80
4.93	4.75	4.87	5.12	4.75
4.89	4.70	4.86	5.20	4.70
5.01	4.86	5.00	5.27	4.86
4.91	4.75	4.91	5.30	4.75
4.99	4.84	4.98	5.25	4.84
4.93	4.82	4.97	5.33	4.82
5.00	4.94	5.03	5.28	4.94
4.96	4.90	4.98	5.34	4.90
5.05	5.04	5.06	5.29	5.04
			4.10	3.80
			4.22	3.89
			4.36	3.97
			4.33	3.87
			4.45	3.94
			4.50	4.04
			4.40	3.93
			4.50	4.00
			4.59	4.14
			4.46	4.03
			4.67	4.26
			4.74	4.41
			4.63	4.27
			4.65	4.39
			4.74	4.44
			4.67	4.49
			4.80	4.56
			4.84	4.69
			4.75	4.63
			4.92	4.76
			5.14	4.93
			5.04	4.87
			5.12	4.86
			5.15	5.00
			5.15	4.91
			5.15	4.98
			5.21	4.97
			5.20	5.03

			5.32	4.98
			5.30	5.06

Figure 3

Panel A

Riverine, ♂	Riverine, ♀	Estuarine, ♂	Estuarine, ♀	Marine, ♂	Marine, ♀
63.37	56.23	48.40	47.60	66.75	60.00
62.55	63.12	54.48	51.18	59.31	60.86
64.24	56.81	50.08	48.63	68.85	60.81
63.11	64.08	55.63	51.89	61.85	61.72
59.84	54.60	49.59	47.51	66.64	56.84
63.84	64.87	55.78	52.32	62.54	62.49
65.07	58.92	51.56	49.23	71.59	62.00
63.84	65.60	56.59	52.56	63.22	62.63
65.77	59.21	52.05	49.49	72.52	63.42
64.61	65.97	57.32	52.83	63.78	64.21
65.99	59.96	52.86	49.97	72.72	64.56
65.08	67.30	58.20	53.46	64.47	66.37
67.04	60.58	54.40	50.30	73.40	65.84
66.07	67.49	60.12	53.66	65.03	67.95
67.48	60.99	55.14	52.08	74.23	67.69
66.92	68.73	60.42	56.50	65.33	69.58
69.96	62.15	55.42	53.56	74.77	69.17
69.15	69.56	61.38	57.76	65.85	70.64
70.58	62.65	56.96	54.56	75.21	71.49
69.62	71.31	62.57	58.51	67.69	72.70
71.01	64.22	57.49	55.04	77.12	72.49
69.71	71.54	62.84	58.86	69.20	73.56
71.71	64.59	57.66	55.49	79.17	73.58
71.08	71.81	63.91	59.57	69.89	74.76
72.50	64.88	58.89	56.12	79.56	74.43
81.72	80.23	81.91	77.85	83.90	83.41
73.33	65.17	59.21	56.90	80.49	75.29
72.07	72.50	64.84	61.38	71.52	76.05
73.72	65.59	59.35	57.93	82.30	75.43
72.71	73.38	64.99	62.68	72.98	76.58

Panel B

Pre-spawning, ♂	Spawning, ♂	Post-spawning, ♂	Pre-spawning, ♀	Spawning, ♀	Post-spawning, ♀
65.99	63.37	71.01	59.96	56.23	64.22
65.08	62.55	69.71	67.30	63.12	71.54
67.04	64.24	71.71	60.58	56.81	64.59
66.07	63.11	71.08	67.49	64.08	71.81
67.48	59.84	72.50	60.99	54.60	64.88
66.92	63.84	81.72	68.73	64.87	80.23
69.96	65.07	73.33	62.15	58.92	65.17
69.15	63.84	72.07	69.56	65.60	72.50
70.58	65.77	73.72	62.65	59.21	65.59
69.62	64.61	72.71	71.31	65.97	73.38
52.86	48.40	57.49	49.97	47.60	55.04
58.20	54.48	62.84	53.46	51.18	58.86
54.40	50.08	57.66	50.30	48.63	55.49
60.12	55.63	63.91	53.66	51.89	59.57
55.14	49.59	58.89	52.08	47.51	56.12
60.42	55.78	81.91	56.50	52.32	77.85
55.42	51.56	59.21	53.56	49.23	56.90
61.38	56.59	64.84	57.76	52.56	61.38
56.96	52.05	59.35	54.56	49.49	57.93
62.57	57.32	64.99	58.51	52.83	62.68
72.72	66.75	77.12	64.56	60.00	72.49
64.47	59.31	69.20	66.37	60.86	73.56
73.40	68.85	79.17	65.84	60.81	73.58
65.03	61.85	69.89	67.95	61.72	74.76
74.23	66.64	79.56	67.69	56.84	74.43
65.33	62.54	83.90	69.58	62.49	83.41
74.77	71.59	80.49	69.17	62.00	75.29
65.85	63.22	71.52	70.64	62.63	76.05
75.21	72.52	82.30	71.49	63.42	75.43
67.69	63.78	72.98	72.70	64.21	76.58

Panel C

>23–40, ♂	>23–40, ♀	41–60, ♂	41–60, ♀	60+, ♂	60+, ♀
63.37	56.23	58.20	53.46	65.03	70.64
62.55	63.12	54.40	50.30	74.23	71.49
64.24	56.81	60.12	53.66	65.33	72.70
63.11	64.08	55.14	52.08	74.77	72.49
59.84	54.60	60.42	56.50	69.20	73.56
63.84	64.87	62.84	58.86	79.17	73.58
65.07	58.92	57.66	55.49	69.89	74.76
63.84	65.60	63.91	59.57	79.56	74.43
65.77	59.21	58.89	56.12	83.90	83.41
64.61	65.97	81.91	77.85	80.49	75.29
65.99	59.96	59.21	56.90	71.52	76.05
65.08	67.30	64.84	61.38	82.30	75.43

67.04	60.58	59.35	57.93	72.98	76.58
66.07	67.49	64.99	62.68		72.70
67.48	60.99	66.75	60.00		72.49
66.92	68.73	59.31	60.86		
69.96	62.15	68.85	60.81		
69.15	69.56	61.85	61.72		
70.58	62.65	66.64	56.84		
69.62	71.31	62.54	62.49		
71.01	64.22	71.59	62.00		
69.71	71.54	63.22	62.63		
71.71	64.59	72.52	63.42		
71.08	71.81	63.78	64.21		
72.50	64.88	72.72	64.56		
81.72	80.23	64.47	66.37		
73.33	65.17	73.40			
72.07	72.50	65.85			
73.72	65.59	75.21			
72.71	73.38	67.69			
48.40	47.60	77.12			
54.48	51.18				
50.08	48.63				
55.63	51.89				
49.59	47.51				
55.78	52.32				
51.56	49.23				
56.59	52.56				
52.05	49.49				
57.32	52.83				
52.86	49.97				
55.42	53.56				
61.38	57.76				
56.96	54.56				
62.57	58.51				
57.49	55.04				
	65.84				
	70.64				
	71.49				

Riverine, both	Estuarine, both	Marine, both	All, ♂	All, ♀
63.37	48.40	66.75	63.37	56.23
62.55	54.48	59.31	62.55	63.12
64.24	50.08	68.85	64.24	56.81
63.11	55.63	61.85	63.11	64.08
59.84	49.59	66.64	59.84	54.60
63.84	55.78	62.54	63.84	64.87
65.07	51.56	71.59	65.07	58.92
63.84	56.59	63.22	63.84	65.60
65.77	52.05	72.52	65.77	59.21
64.61	57.32	63.78	64.61	65.97
65.99	52.86	72.72	65.99	59.96
65.08	58.20	64.47	65.08	67.30
67.04	54.40	73.40	67.04	60.58
66.07	60.12	65.03	66.07	67.49
67.48	55.14	74.23	67.48	60.99
66.92	60.42	65.33	66.92	68.73
69.96	55.42	74.77	69.96	62.15
69.15	61.38	65.85	69.15	69.56
70.58	56.96	75.21	70.58	62.65
69.62	62.57	67.69	69.62	71.31
71.01	57.49	77.12	71.01	64.22
69.71	62.84	69.20	69.71	71.54
71.71	57.66	79.17	71.71	64.59
71.08	63.91	69.89	71.08	71.81
72.50	58.89	79.56	72.50	64.88
81.72	81.91	83.90	81.72	80.23
73.33	59.21	80.49	73.33	65.17
72.07	64.84	71.52	72.07	72.50
73.72	59.35	82.30	73.72	65.59
72.71	64.99	72.98	72.71	73.38
56.23	47.60	60.00	48.40	47.60
63.12	51.18	60.86	54.48	51.18
56.81	48.63	60.81	50.08	48.63
64.08	51.89	61.72	55.63	51.89
54.60	47.51	56.84	49.59	47.51
64.87	52.32	62.49	55.78	52.32
58.92	49.23	62.00	51.56	49.23
65.60	52.56	62.63	56.59	52.56
59.21	49.49	63.42	52.05	49.49
65.97	52.83	64.21	57.32	52.83
59.96	49.97	64.56	52.86	49.97
67.30	53.46	66.37	58.20	53.46
60.58	50.30	65.84	54.40	50.30
67.49	53.66	67.95	60.12	53.66

60.99	52.08	67.69	55.14	52.08
68.73	56.50	69.58	60.42	56.50
62.15	53.56	69.17	55.42	53.56
69.56	57.76	70.64	61.38	57.76
62.65	54.56	71.49	56.96	54.56
71.31	58.51	72.70	62.57	58.51
64.22	55.04	72.49	57.49	55.04
71.54	58.86	73.56	62.84	58.86
64.59	55.49	73.58	57.66	55.49
71.81	59.57	74.76	63.91	59.57
64.88	56.12	74.43	58.89	56.12
80.23	77.85	83.41	81.91	77.85
65.17	56.90	75.29	59.21	56.90
72.50	61.38	76.05	64.84	61.38
65.59	57.93	75.43	59.35	57.93
73.38	62.68	76.58	64.99	62.68
			66.75	60.00
			59.31	60.86
			68.85	60.81
			61.85	61.72
			66.64	56.84
			62.54	62.49
			71.59	62.00
			63.22	62.63
			72.52	63.42
			63.78	64.21
			72.72	64.56
			64.47	66.37
			73.40	65.84
			65.03	67.95
			74.23	67.69
			65.33	69.58
			74.77	69.17
			65.85	70.64
			75.21	71.49
			67.69	72.70
			77.12	72.49
			69.20	73.56
			79.17	73.58
			69.89	74.76
			79.56	74.43
			83.90	83.41
			80.49	75.29
			71.52	76.05
			82.30	75.43
			72.98	76.58

S1 Figure

Panel A

Kirtankhola	U. Padma	Meghna	Meghna	Tentualia	Pashur	Maheshkhali	Kuakata	Mongla
26.73	25.48	28.99	29.09	25.72	27.89	25.38	27.33	31.26
27.24	29.79	27.83	26.94	28.57	31.44	32.87	32.67	27.81
31.72	31.94	27.49	26.75	30.98	29.56	28.25	25.36	28.84
26.34	26.66	31.29	28.82	27.49	29.56	29.44	30.12	26.29
29.72	30.02	32.28	29.45	28.41	25.88	30.81	28.24	29.66
32.30	27.73	26.02	32.73	27.96	28.05	25.20	29.08	25.46
31.69	25.42	29.49	28.94	32.12	26.61	31.14	28.97	28.53
29.92	27.66	27.93	27.93	26.53	31.88	28.22	32.12	24.32
30.73	30.26	29.08	26.55	25.44	26.63	25.61	27.34	32.67

Panel B

Kirtankhola	U. Padma	Meghna	Meghna	Tentualia	Pashur	Maheshkhali	Kuakata	Khulna
21.67	25.94	30.90	23.60	20.35	23.84	27.29	23.58	28.37
21.03	27.64	26.87	24.38	21.62	28.07	22.16	28.56	21.24
26.26	25.04	28.97	23.22	22.11	21.59	26.91	28.87	22.52
23.75	22.77	23.92	27.37	25.83	25.84	22.33	20.57	28.05
24.17	27.77	24.82	27.82	26.50	20.49	26.02	21.80	25.10
26.42	26.72	25.31	23.22	28.43	27.21	26.81	21.06	20.59
25.78	24.38	23.98	25.92	27.35	28.06	25.79	28.20	21.24
23.71	26.52	27.19	28.20	23.57	23.61	25.24	26.19	23.30
22.68	23.22	26.41	27.46	22.05	27.74	20.35	24.77	27.30

Panel C

Kirtankhola	U. Padma	Meghna	Meghna	Tentualia	Pashur	Maheshkhali	Kuakata	Mongla
61.00	41.00	60.00	1245.00	592.00	1792.00	4081.00	6472.00	5709.00
232.00	216.00	117.00	1379.00	635.00	1009.00	4555.00	3677.00	6063.00
122.00	54.00	80.00	1450.00	769.00	923.00	6956.00	3392.00	4552.00
143.00	173.00	129.00	957.00	863.00	812.00	6602.00	3782.00	6848.00
193.00	39.00	232.00	1644.00	982.00	736.00	6017.00	7886.00	7845.00
235.00	164.00	168.00	915.00	1012.00	1130.00	6136.00	7000.00	4579.00
97.00	63.00	205.00	1107.00	1162.00	1338.00	4975.00	7678.00	3738.00
233.00	151.00	131.00	1583.00	964.00	1122.00	5255.00	8341.00	4578.00
235.00	118.00	220.00	1723.00	720.00	980.00	6923.00	7008.00	5921.00

Panel D

Kirtankhola	U. Padma	Meghna	Meghna	Tentualia	Pashur	Maheshkhali	Kuakata	Mongla
39.00	26.00	38.00	351.00	148.00	448.00	1551.00	2783.00	2112.00
146.00	136.00	74.00	345.00	159.00	252.00	1731.00	2366.00	2243.00
77.00	34.00	50.00	363.00	192.00	261.00	2643.00	2459.00	1684.00
90.00	109.00	81.00	239.00	216.00	203.00	2509.00	2626.00	2534.00
122.00	25.00	146.00	411.00	246.00	154.00	2286.00	3391.00	2903.00
148.00	103.00	106.00	189.00	253.00	283.00	2332.00	3010.00	1694.00
61.00	40.00	129.00	277.00	291.00	335.00	1891.00	3301.00	2383.00
147.00	95.00	83.00	396.00	241.00	281.00	1997.00	3587.00	1694.00

148.00	74.00	139.00	431.00	180.00	245.00	2631.00	3013.00	2191.00

Panel E

Kirtankhola	U. Padma	Meghna	Meghna	Tentualia	Pashur	Maheshkhali	Kuakata	Mongla
222.00	308.00	499.00	6777.00	8629.00	8593.00	32256.00	35518.00	34936.00
634.00	460.00	420.00	7015.00	7946.00	9115.00	32395.00	35684.00	34989.00
276.00	362.00	338.00	8679.00	7241.00	9411.00	30085.00	35029.00	33163.00
631.00	304.00	337.00	7599.00	7046.00	7552.00	32205.00	33516.00	34913.00
256.00	179.00	637.00	8609.00	7139.00	9714.00	30290.00	35239.00	33645.00
518.00	184.00	189.00	6874.00	7036.00	9061.00	30663.00	35782.00	34014.00
286.00	574.00	568.00	8972.00	6784.00	9518.00	30920.00	34049.00	34049.00
553.00	299.00	503.00	7405.00	8878.00	9493.00	31968.00	33466.00	34320.00
534.00	334.00	349.00	6966.00	9000.00	8265.00	31122.00	34273.00	34299.00

S2 Figure

Panel A

Kirtankhola	U. Padma	Meghna	Meghna	Tentualia	Pashur	Maheshkhali	Kuakata	Mongla
7.62	7.51	7.58	7.71	7.80	7.77	8.02	7.90	7.82
7.47	7.54	7.59	7.88	7.92	7.87	8.06	8.01	7.90
7.48	7.54	7.62	7.72	7.82	7.77	8.03	7.96	7.83
7.49	7.54	7.62	7.73	7.83	7.77	8.03	7.96	7.83
7.49	7.54	7.62	7.73	7.84	7.72	8.04	7.96	7.84
7.49	7.55	7.63	7.73	7.84	7.79	8.05	7.98	7.87
7.49	7.56	7.64	7.74	7.87	7.80	8.06	7.98	7.88
7.50	7.57	7.64	7.84	7.78	7.94	8.03	8.05	7.98
7.51	7.57	7.65	7.76	7.88	7.80	8.07	8.02	7.90

Panel B

Kirtankhola	U. Padma	Meghna	Meghna	Tentualia	Pashur	Maheshkhali	Kuakata	Mongla
1164.00	1021.00	1075.00	8246.00	13111.00	11040.00	32353.00	38002.00	34085.00
939.00	1000.00	964.00	9033.00	12768.00	11079.00	34396.00	38136.00	34252.00
1129.00	1023.00	1190.00	8321.00	13873.00	11098.00	33077.00	38555.00	34480.00
908.00	932.00	956.00	9351.00	12082.00	11239.00	33311.00	37995.00	34668.00
913.00	965.00	1003.00	8450.00	14112.00	11422.00	33360.00	36241.00	34938.00
944.00	974.00	1013.00	9795.00	12344.00	11793.00	33439.00	36297.00	35401.00
963.00	1223.00	1254.00	9331.00	14528.00	10345.00	33479.00	36391.00	37035.00
970.00	1099.00	1298.00	9512.00	12567.00	10625.00	33881.00	37488.00	37775.00
1069.00	1119.00	1399.00	10487.00	14951.00	10825.00	34015.00	38734.00	37806.00

Panel C

Kirtankhola	U. Padma	Meghna	Meghna	Tentualia	Pashur	Maheshkhali	Kuakata	Mongla
74.00	52.00	94.00	70.00	96.00	95.00	100.00	112.00	91.00
84.00	61.00	86.00	66.00	76.00	93.00	93.00	88.00	115.00
67.00	95.00	72.00	76.00	92.00	91.00	86.00	102.00	116.00
92.00	94.00	65.00	88.00	94.00	86.00	101.00	85.00	90.00
65.00	52.00	55.00	88.00	76.00	64.00	86.00	112.00	116.00
72.00	53.00	97.00	65.00	84.00	94.00	89.00	115.00	100.00
83.00	78.00	80.00	91.00	76.00	70.00	104.00	105.00	103.00
85.00	75.00	70.00	92.00	86.00	85.00	82.00	83.00	99.00
85.00	96.00	57.00	68.00	94.00	64.00	89.00	80.00	99.00

Panel D

Kirtankhola	U. Padma	Meghna	Meghna	Tentualia	Pashur	Maheshkhali	Kuakata	Mongla
6.96	7.38	7.62	6.72	7.91	7.47	8.26	7.92	7.35
6.96	7.37	6.67	7.65	7.17	8.07	7.50	7.54	8.24
8.01	6.74	6.92	8.31	7.68	8.07	7.29	7.76	7.45
7.57	6.78	7.03	6.92	8.25	7.41	7.35	7.51	8.19
6.79	6.95	7.79	6.89	7.66	6.81	7.81	7.73	7.21
7.37	7.33	7.97	7.74	8.10	6.91	7.21	7.19	7.35
7.95	7.35	7.40	7.79	7.97	8.32	8.13	7.93	7.35
7.35	6.55	7.27	8.04	7.56	6.95	7.68	7.76	7.69

7.88	6.34	8.30	8.22	7.31	7.78	7.36	7.58	7.96

Panel E

Kirtankhola	U. Padma	Meghna	Meghna	Tentualia	Pashur	Maheshkhali	Kuakata	Mongla
0.02	0.01	0.03	0.02	0.03	0.03	0.03	0.02	0.03
0.01	0.03	0.04	0.03	0.04	0.03	0.02	0.01	0.05
0.02	0.04	0.04	0.02	0.01	0.03	0.01	0.04	0.01
0.02	0.02	0.02	0.02	0.02	0.03	0.02	0.03	0.02
0.05	0.01	0.03	0.04	0.03	0.02	0.04	0.03	0.05
0.04	0.05	0.04	0.03	0.04	0.04	0.04	0.04	0.03
0.03	0.03	0.02	0.03	0.03	0.05	0.04	0.02	0.03
0.02	0.01	0.02	0.02	0.02	0.04	0.03	0.01	0.02
0.01	0.02	0.03	0.03	0.05	0.03	0.04	0.03	0.05

Panel F

Kirtankhola	U. Padma	Meghna	Meghna	Tentualia	Pashur	Maheshkhali	Kuakata	Mongla
0.01	0.01	0.00	0.00	0.01	0.01	0.01	0.00	0.01
0.01	0.00	0.01	0.00	0.00	0.01	0.00	0.01	0.01
0.01	0.01	0.00	0.00	0.01	0.01	0.00	0.00	0.01
0.00	0.01	0.00	0.01	0.01	0.01	0.01	0.01	0.01
0.01	0.01	0.00	0.01	0.00	0.01	0.01	0.01	0.01
0.00	0.00	0.01	0.00	0.00	0.01	0.01	0.00	0.01
0.01	0.01	0.01	0.00	0.00	0.00	0.01	0.01	0.01
0.01	0.01	0.01	0.01	0.00	0.01	0.01	0.01	0.01
0.01	0.01	0.01	0.01	0.01	0.01	0.00	0.01	0.01

Panel G

Kirtankhola	U. Padma	Meghna	Meghna	Tentualia	Pashur	Maheshkhali	Kuakata	Mongla
0.09	0.03	0.14	0.25	0.33	0.11	0.24	0.48	0.25
0.15	0.07	0.08	0.09	0.27	0.35	0.31	0.44	0.40
0.05	0.07	0.07	0.18	0.23	0.33	0.45	0.25	0.37
0.06	0.09	0.06	0.32	0.30	0.21	0.33	0.48	0.31
0.16	0.08	0.09	0.22	0.19	0.22	0.44	0.33	0.27
0.06	0.12	0.15	0.33	0.12	0.27	0.26	0.42	0.47
0.12	0.05	0.06	0.24	0.07	0.27	0.37	0.48	0.37
0.07	0.15	0.06	0.10	0.17	0.25	0.27	0.34	0.47
0.07	0.05	0.10	0.08	0.18	0.12	0.33	0.26	0.37

Panel H

Kirtankhola	U. Padma	Meghna	Meghna	Tentualia	Pashur	Maheshkhali	Kuakata	Mongla
0.27	0.21	0.22	0.08	0.13	0.08	0.28	0.27	0.27
0.28	0.06	0.30	0.15	0.28	0.35	0.44	0.37	0.51
0.24	0.20	0.24	0.11	0.34	0.15	0.39	0.33	0.51
0.23	0.23	0.04	0.32	0.12	0.12	0.34	0.37	0.49
0.26	0.15	0.04	0.38	0.29	0.12	0.50	0.52	0.37
0.27	0.30	0.28	0.13	0.33	0.22	0.52	0.32	0.47
0.22	0.11	0.26	0.23	0.10	0.19	0.39	0.37	0.38

0.19	0.07	0.23	0.34	0.13	0.10	0.33	0.31	0.30
0.27	0.18	0.17	0.22	0.29	0.22	0.48	0.49	0.45

S3 Figure

Panel A

Kirtankhola	U. Padma	Meghna	Meghna	Tentualia	Pashur	Maheshkhali	Kuakata	Mongla
8.80	7.18	5.74	4.89	3.61	5.91	4.04	3.22	5.13
8.99	5.29	8.47	3.91	3.68	4.90	4.40	4.41	5.14
5.51	8.70	8.21	3.70	3.48	5.32	2.88	5.32	4.61
8.57	8.88	8.92	4.27	3.79	5.89	3.34	4.69	5.63
6.13	8.07	8.98	3.32	4.11	5.46	3.78	3.10	3.56
8.48	8.90	6.52	4.00	3.71	5.18	4.19	4.83	3.48
8.68	5.75	8.96	3.38	3.79	5.23	5.26	3.39	5.66
5.99	5.78	8.45	4.40	4.56	5.75	4.05	5.49	5.60
8.61	8.94	8.93	3.43	3.81	4.86	4.32	3.61	3.97

Panel B

Kirtankhola	U. Padma	Meghna	Meghna	Tentualia	Pashur	Maheshkhali	Kuakata	Mongla
5.36	5.13	3.54	7.78	4.21	3.59	2.74	1.50	2.02
4.65	3.24	6.97	2.79	4.20	3.60	2.21	0.90	1.93
3.84	2.63	7.85	7.97	4.31	3.36	1.78	1.31	3.00
5.24	4.30	7.69	7.54	3.63	3.14	0.89	1.85	2.59
5.13	4.51	6.85	7.79	4.99	3.42	2.23	2.10	2.69
5.36	5.22	4.50	4.12	3.35	3.35	1.83	2.06	2.35
4.33	2.70	7.63	5.92	3.55	3.45	1.57	1.44	2.12
5.49	4.74	7.41	7.57	3.66	3.91	1.92	1.02	2.03
5.91	3.94	2.64	7.48	3.54	3.34	1.20	2.66	2.77

Panel C

Kirtankhola	U. Padma	Meghna	Meghna	Tentualia	Pashur	Maheshkhali	Kuakata	Mongla
361.81	310.22	578.71	609.72	361.81	530.53	425.69	291.96	251.92
346.15	237.07	586.28	635.16	346.15	491.40	372.96	260.40	262.92
381.47	234.92	563.69	644.40	381.47	417.66	260.78	285.63	345.17
335.42	293.32	688.99	462.12	335.42	586.04	500.50	277.55	344.89
383.97	275.75	674.45	476.83	383.97	472.01	424.27	238.70	284.13
326.87	248.20	696.59	416.93	426.87	617.55	461.21	306.89	334.19
315.97	233.17	419.89	524.24	415.97	444.61	336.70	286.95	260.26
247.44	256.41	423.24	613.29	447.44	561.96	380.77	270.48	271.32
254.31	303.44	414.11	564.58	454.31	596.29	465.04	315.83	325.33

S4 Figure

Panel A

Riverine, ♂	Riverine, ♀	Estuarine, ♂	Estuarine, ♀	Marine, ♂	Marine, ♀
13.00	13.00	14.00	14.00	15.00	13.00
12.00	13.00	15.00	14.00	12.00	14.00
15.00	15.00	15.00	15.00	13.00	13.00
13.00	14.00	12.00	12.00	14.00	15.00
14.00	13.00	12.00	14.00	13.00	13.00
15.00	15.00	15.00	14.00	13.00	15.00
12.00	14.00	15.00	15.00	15.00	13.00
15.00	15.00	13.00	12.00	14.00	15.00
15.00	15.00	14.00	13.00	13.00	12.00
13.00	14.00	15.00	14.00	13.00	12.00
14.00	15.00	15.00	14.00	12.00	13.00
15.00	15.00	12.00	14.00	15.00	14.00
13.00	14.00	15.00	12.00	12.00	13.00
12.00	15.00	12.00	12.00	15.00	14.00
14.00	13.00	13.00	13.00	15.00	12.00
14.00	13.00	14.00	14.00	13.00	14.00
15.00	14.00	13.00	13.00	13.00	14.00
15.00	13.00	15.00	13.00	12.00	13.00
14.00	13.00	15.00	12.00	13.00	14.00
16.00	12.00	14.00	14.00	15.00	13.00
13.00	12.00	12.00	12.00	14.00	15.00
14.00	14.00	14.00	14.00	12.00	13.00
15.00	14.00	13.00	14.00	13.00	15.00
13.00	16.00	12.00	15.00	15.00	13.00
14.00	12.00	13.00	15.00	12.00	12.00
15.00	14.00	13.00	15.00	13.00	12.00
12.00	12.00	12.00	14.00	12.00	13.00
13.00	15.00	12.00	14.00	13.00	14.00
14.00	13.00	15.00	12.00	13.00	13.00
14.00	14.00	12.00	14.00	15.00	15.00

Panel B

Pre-spawning, ♂	Spawning, ♂	Post-spawning, ♂	Pre-spawning, ♀	Spawning, ♀	Post-spawning, ♀
14.00	13.00	13.00	15.00	13.00	12.00
15.00	12.00	14.00	15.00	13.00	14.00
13.00	15.00	15.00	14.00	15.00	14.00
12.00	13.00	13.00	15.00	14.00	16.00
14.00	14.00	14.00	13.00	13.00	12.00
14.00	15.00	15.00	13.00	15.00	14.00
15.00	12.00	12.00	14.00	14.00	12.00
15.00	15.00	13.00	13.00	15.00	15.00
14.00	15.00	14.00	13.00	15.00	13.00
16.00	13.00	14.00	12.00	14.00	14.00
15.00	14.00	12.00	14.00	14.00	12.00
12.00	15.00	14.00	14.00	14.00	14.00
15.00	15.00	13.00	12.00	15.00	14.00
12.00	12.00	12.00	12.00	12.00	15.00
13.00	12.00	13.00	13.00	14.00	15.00
14.00	15.00	13.00	14.00	14.00	15.00
13.00	15.00	12.00	13.00	15.00	14.00
15.00	13.00	12.00	13.00	12.00	14.00
15.00	14.00	15.00	12.00	13.00	12.00
14.00	15.00	12.00	14.00	14.00	14.00
12.00	15.00	14.00	13.00	13.00	15.00
15.00	12.00	12.00	14.00	14.00	13.00
12.00	13.00	13.00	13.00	13.00	15.00
15.00	14.00	15.00	14.00	15.00	13.00
15.00	13.00	12.00	12.00	13.00	12.00
13.00	13.00	13.00	14.00	15.00	12.00
13.00	15.00	12.00	14.00	13.00	13.00
12.00	14.00	13.00	13.00	15.00	14.00
13.00	13.00	13.00	14.00	12.00	13.00
15.00	13.00	15.00	13.00	12.00	15.00

Panel C

>23–40, ♂	>23–40, ♀	41–60, ♂	41–60, ♀	60+, ♂	60+, ♀
13.00	13.00	12.00	14.00	15.00	13.00
12.00	13.00	15.00	12.00	15.00	14.00
15.00	15.00	12.00	12.00	13.00	13.00
13.00	14.00	13.00	13.00	13.00	15.00
14.00	13.00	14.00	14.00	12.00	13.00
15.00	15.00	14.00	14.00	13.00	15.00
12.00	14.00	13.00	14.00	15.00	13.00
15.00	15.00	12.00	15.00	12.00	12.00
15.00	15.00	13.00	15.00	13.00	12.00
13.00	14.00	13.00	15.00	12.00	13.00
14.00	15.00	12.00	14.00	13.00	14.00
15.00	15.00	12.00	14.00	13.00	13.00

13.00	14.00	15.00	12.00	15.00	15.00
12.00	15.00	12.00	14.00		13.00
14.00	13.00	15.00	13.00		15.00
14.00	13.00	12.00	14.00		
15.00	14.00	13.00	13.00		
15.00	13.00	14.00	15.00		
14.00	13.00	13.00	13.00		
16.00	12.00	13.00	15.00		
13.00	12.00	15.00	13.00		
14.00	14.00	14.00	15.00		
15.00	14.00	13.00	12.00		
13.00	16.00	13.00	12.00		
14.00	12.00	12.00	13.00		
15.00	14.00	15.00	14.00		
12.00	12.00	12.00			
13.00	15.00	12.00			
14.00	13.00	13.00			
14.00	14.00	15.00			
14.00	14.00	14.00			
15.00	14.00				
15.00	15.00				
12.00	12.00				
12.00	14.00				
15.00	14.00				
15.00	15.00				
13.00	12.00				
14.00	13.00				
15.00	14.00				
15.00	14.00				
13.00	13.00				
15.00	13.00				
15.00	12.00				
14.00	14.00				
12.00	12.00				
	13.00				
	13.00				
	14.00				

Panel D

Riverine, ♂	Riverine, ♀	Estuarine, ♂	Estuarine, ♀	Marine, ♂	Marine, ♀
8.67	8.67	9.33	9.33	10.00	8.67
8.00	8.67	10.00	9.33	8.00	9.33
10.00	10.00	10.00	10.00	8.67	8.67
8.67	9.33	8.00	8.00	9.33	10.00
9.33	8.67	8.00	9.33	8.67	8.67
10.00	10.00	10.00	9.33	8.67	10.00
8.00	9.33	10.00	10.00	10.00	8.67

10.00	10.00	8.67	8.00	9.33	10.00
10.00	10.00	9.33	8.67	8.67	8.00
8.67	9.33	10.00	9.33	8.67	8.00
9.33	10.00	10.00	9.33	8.00	8.67
10.00	10.00	8.00	9.33	10.00	9.33
8.67	9.33	10.00	8.00	8.00	8.67
8.00	10.00	8.00	8.00	10.00	9.33
9.33	8.67	8.67	8.67	10.00	8.00
9.33	8.67	9.33	9.33	8.67	9.33
10.00	9.33	8.67	8.67	8.67	9.33
10.00	8.67	10.00	8.67	8.00	8.67
9.33	8.67	10.00	8.00	8.67	9.33
10.67	8.00	9.33	9.33	10.00	8.67
8.67	8.00	8.00	8.00	9.33	10.00
9.33	9.33	9.33	9.33	8.00	8.67
10.00	9.33	8.67	9.33	8.67	10.00
8.67	10.67	8.00	10.00	10.00	8.67
9.33	8.00	8.67	10.00	8.00	8.00
10.00	9.33	8.67	10.00	8.67	8.00
8.00	8.00	8.00	9.33	8.00	8.67
8.67	10.00	8.00	9.33	8.67	9.33
9.33	8.67	10.00	8.00	8.67	8.67
9.33	9.33	8.00	9.33	10.00	10.00

Panel E

Pre-spawning, ♂	Spawning, ♂	Post-spawning, ♂	Pre-spawning, ♀	Spawning, ♀	Post-spawning, ♀
9.33	8.67	8.67	10.00	8.67	8.00
10.00	8.00	9.33	10.00	8.67	9.33
8.67	10.00	10.00	9.33	10.00	9.33
8.00	8.67	8.67	10.00	9.33	10.67
9.33	9.33	9.33	8.67	8.67	8.00
9.33	10.00	10.00	8.67	10.00	9.33
10.00	8.00	8.00	9.33	9.33	8.00
10.00	10.00	8.67	8.67	10.00	10.00

9.33	10.00	9.33	8.67	10.00	8.67
10.67	8.67	9.33	8.00	9.33	9.33
10.00	9.33	8.00	9.33	9.33	8.00
8.00	10.00	9.33	9.33	9.33	9.33
10.00	10.00	8.67	8.00	10.00	9.33
8.00	8.00	8.00	8.00	8.00	10.00
8.67	8.00	8.67	8.67	9.33	10.00
9.33	10.00	8.67	9.33	9.33	10.00
8.67	10.00	8.00	8.67	10.00	9.33
10.00	8.67	8.00	8.67	8.00	9.33
10.00	9.33	10.00	8.00	8.67	8.00
9.33	10.00	8.00	9.33	9.33	9.33
8.00	10.00	9.33	8.67	8.67	10.00
10.00	8.00	8.00	9.33	9.33	8.67
8.00	8.67	8.67	8.67	8.67	10.00
10.00	9.33	10.00	9.33	10.00	8.67
10.00	8.67	8.00	8.00	8.67	8.00
8.67	8.67	8.67	9.33	10.00	8.00
8.67	10.00	8.00	9.33	8.67	8.67
8.00	9.33	8.67	8.67	10.00	9.33
8.67	8.67	8.67	9.33	8.00	8.67
10.00	8.67	10.00	8.67	8.00	10.00

Panel F

>23–40, ♂	>23–40, ♀	41–60, ♂	41–60, ♀	60+, ♂	60+, ♀
8.67	8.67	8.00	9.33	10.00	8.67
8.00	8.67	10.00	8.00	10.00	9.33
10.00	10.00	8.00	8.00	8.67	8.67
8.67	9.33	8.67	8.67	8.67	10.00
9.33	8.67	9.33	9.33	8.00	8.67
10.00	10.00	9.33	9.33	8.67	10.00
8.00	9.33	8.67	9.33	10.00	8.67
10.00	10.00	8.00	10.00	8.00	8.00
10.00	10.00	8.67	10.00	8.67	8.00
8.67	9.33	8.67	10.00	8.00	8.67
9.33	10.00	8.00	9.33	8.67	9.33
10.00	10.00	8.00	9.33	8.67	8.67
8.67	9.33	10.00	8.00	10.00	10.00
8.00	10.00	8.00	9.33		8.67
9.33	8.67	10.00	8.67		10.00
9.33	8.67	8.00	9.33		
10.00	9.33	8.67	8.67		
10.00	8.67	9.33	10.00		
9.33	8.67	8.67	8.67		
10.67	8.00	8.67	10.00		
8.67	8.00	10.00	8.67		
9.33	9.33	9.33	10.00		

10.00	9.33	8.67	8.00		
8.67	10.67	8.67	8.00		
9.33	8.00	8.00	8.67		
10.00	9.33	10.00	9.33		
8.00	8.00	8.00			
8.67	10.00	8.00			
9.33	8.67	8.67			
9.33	9.33	10.00			
9.33	9.33	9.33			
10.00	9.33				
10.00	10.00				
8.00	8.00				
8.00	9.33				
10.00	9.33				
10.00	10.00				
8.67	8.00				
9.33	8.67				
10.00	9.33				
10.00	9.33				
8.67	8.67				
10.00	8.67				
10.00	8.00				
9.33	9.33				
8.00	8.00				
	8.67				
	8.67				
	9.33				

Panel G

Riverine, ♂	Riverine, ♀	Estuarine, ♂	Estuarine, ♀	Marine, ♂	Marine, ♀
88.48	88.48	102.62	102.62	117.80	88.48
75.39	88.48	117.80	102.62	75.39	102.62
117.80	117.80	117.80	117.80	88.48	88.48
88.48	102.62	75.39	75.39	102.62	117.80
102.62	88.48	75.39	102.62	88.48	88.48
117.80	117.80	117.80	102.62	88.48	117.80
75.39	102.62	117.80	117.80	117.80	88.48
117.80	117.80	88.48	75.39	102.62	117.80
117.80	117.80	102.62	88.48	88.48	75.39
88.48	102.62	117.80	102.62	88.48	75.39
102.62	117.80	117.80	102.62	75.39	88.48
117.80	117.80	75.39	102.62	117.80	102.62
88.48	102.62	117.80	75.39	75.39	88.48
75.39	117.80	75.39	75.39	117.80	102.62
102.62	88.48	88.48	88.48	117.80	75.39
102.62	88.48	102.62	102.62	88.48	102.62
117.80	102.62	88.48	88.48	88.48	102.62

117.80	88.48	117.80	88.48	75.39	88.48
102.62	88.48	117.80	75.39	88.48	102.62
134.03	75.39	102.62	102.62	117.80	88.48
88.48	75.39	75.39	75.39	102.62	117.80
102.62	102.62	102.62	102.62	75.39	88.48
117.80	102.62	88.48	102.62	88.48	117.80
88.48	134.03	75.39	117.80	117.80	88.48
102.62	75.39	88.48	117.80	75.39	75.39
117.80	102.62	88.48	117.80	88.48	75.39
75.39	75.39	75.39	102.62	75.39	88.48
88.48	117.80	75.39	102.62	88.48	102.62
102.62	88.48	117.80	75.39	88.48	88.48
102.62	102.62	75.39	102.62	117.80	117.80

Panel H

Pre-spawning, ♂	Spawning, ♂	Post-spawning, ♂	Pre-spawning, ♀	Spawning, ♀	Post-spawning, ♀
102.62	88.48	88.48	117.80	88.48	75.39
117.80	75.39	102.62	117.80	88.48	102.62
88.48	117.80	117.80	102.62	117.80	102.62
75.39	88.48	88.48	117.80	102.62	134.03
102.62	102.62	102.62	88.48	88.48	75.39
102.62	117.80	117.80	88.48	117.80	102.62
117.80	75.39	75.39	102.62	102.62	75.39
117.80	117.80	88.48	88.48	117.80	117.80
102.62	117.80	102.62	88.48	117.80	88.48
134.03	88.48	102.62	75.39	102.62	102.62
117.80	102.62	75.39	102.62	102.62	75.39
75.39	117.80	102.62	102.62	102.62	102.62
117.80	117.80	88.48	75.39	117.80	102.62
75.39	75.39	75.39	75.39	75.39	117.80
88.48	75.39	88.48	88.48	102.62	117.80
102.62	117.80	88.48	102.62	102.62	117.80
88.48	117.80	75.39	88.48	117.80	102.62
117.80	88.48	75.39	88.48	75.39	102.62

117.80	102.62	117.80	75.39	88.48	75.39
102.62	117.80	75.39	102.62	102.62	102.62
75.39	117.80	102.62	88.48	88.48	117.80
117.80	75.39	75.39	102.62	102.62	88.48
75.39	88.48	88.48	88.48	88.48	117.80
117.80	102.62	117.80	102.62	117.80	88.48
117.80	88.48	75.39	75.39	88.48	75.39
88.48	88.48	88.48	102.62	117.80	75.39
88.48	117.80	75.39	102.62	88.48	88.48
75.39	102.62	88.48	88.48	117.80	102.62
88.48	88.48	88.48	102.62	75.39	88.48
117.80	88.48	117.80	88.48	75.39	117.80

Panel I

>23–40, ♂	>23–40, ♀	41–60, ♂	41–60, ♀	60+, ♂	60+, ♀
88.48	88.48	75.39	102.62	117.80	88.48
75.39	88.48	117.80	75.39	117.80	102.62
117.80	117.80	75.39	75.39	88.48	88.48
88.48	102.62	88.48	88.48	88.48	117.80
102.62	88.48	102.62	102.62	75.39	88.48
117.80	117.80	102.62	102.62	88.48	117.80
75.39	102.62	88.48	102.62	117.80	88.48
117.80	117.80	75.39	117.80	75.39	75.39
117.80	117.80	88.48	117.80	88.48	75.39
88.48	102.62	88.48	117.80	75.39	88.48
102.62	117.80	75.39	102.62	88.48	102.62
117.80	117.80	75.39	102.62	88.48	88.48
88.48	102.62	117.80	75.39	117.80	117.80
75.39	117.80	75.39	102.62		88.48
102.62	88.48	117.80	88.48		117.80
102.62	88.48	75.39	102.62		
117.80	102.62	88.48	88.48		
117.80	88.48	102.62	117.80		
102.62	88.48	88.48	88.48		
134.03	75.39	88.48	117.80		
88.48	75.39	117.80	88.48		
102.62	102.62	102.62	117.80		
117.80	102.62	88.48	75.39		
88.48	134.03	88.48	75.39		
102.62	75.39	75.39	88.48		
117.80	102.62	117.80	102.62		
75.39	75.39	75.39			
88.48	117.80	75.39			
102.62	88.48	88.48			
102.62	102.62	117.80			
102.62	102.62	102.62			
117.80	102.62				

117.80	117.80				
75.39	75.39				
75.39	102.62				
117.80	102.62				
117.80	117.80				
88.48	75.39				
102.62	88.48				
117.80	102.62				
117.80	102.62				
88.48	88.48				
117.80	88.48				
117.80	75.39				
102.62	102.62				
75.39	75.39				
	88.48				
	88.48				
	102.62				

Panel J

Riverine, ♂	Riverine, ♀	Estuarine, ♂	Estuarine, ♀	Marine, ♂	Marine, ♀
5.00	5.00	6.00	6.00	6.00	5.00
5.00	5.00	6.00	6.00	5.00	6.00
6.00	6.00	6.00	6.00	5.00	5.00
5.00	6.00	5.00	5.00	6.00	6.00
6.00	5.00	5.00	6.00	5.00	5.00
6.00	6.00	6.00	6.00	5.00	6.00
5.00	6.00	6.00	6.00	6.00	5.00
6.00	6.00	5.00	5.00	6.00	6.00
6.00	6.00	6.00	5.00	5.00	5.00
5.00	6.00	6.00	6.00	5.00	5.00
6.00	6.00	6.00	5.00	5.00	5.00
6.00	6.00	5.00	6.00	6.00	6.00
5.00	6.00	6.00	5.00	5.00	5.00
5.00	6.00	5.00	5.00	6.00	6.00
6.00	5.00	5.00	5.00	6.00	5.00
6.00	5.00	6.00	6.00	5.00	6.00
6.00	6.00	5.00	5.00	5.00	6.00
6.00	5.00	6.00	5.00	5.00	5.00
6.00	5.00	6.00	5.00	5.00	6.00
7.00	5.00	6.00	6.00	6.00	5.00
5.00	5.00	5.00	5.00	6.00	6.00
6.00	6.00	6.00	6.00	5.00	5.00
6.00	6.00	5.00	6.00	5.00	6.00
5.00	6.00	5.00	6.00	6.00	5.00
6.00	5.00	5.00	6.00	5.00	5.00
6.00	6.00	5.00	5.00	5.00	5.00
5.00	5.00	5.00	6.00	5.00	5.00

5.00	6.00	5.00	6.00	5.00	6.00
6.00	5.00	6.00	4.00	5.00	5.00
6.00	6.00	5.00	6.00	6.00	6.00

Panel K

Pre-spawning, ♂	Spawning, ♂	Post-spawning, ♂	Pre-spawning, ♀	Spawning, ♀	Post-spawning, ♀
6.00	5.00	5.00	6.00	5.00	5.00
6.00	5.00	6.00	6.00	5.00	6.00
5.00	6.00	6.00	6.00	6.00	6.00
5.00	5.00	5.00	6.00	6.00	6.00
6.00	6.00	6.00	5.00	5.00	5.00
6.00	6.00	6.00	5.00	6.00	6.00
6.00	5.00	5.00	6.00	6.00	5.00
6.00	6.00	5.00	5.00	6.00	6.00
6.00	6.00	6.00	5.00	6.00	5.00
7.00	5.00	6.00	5.00	6.00	6.00
6.00	6.00	5.00	5.00	6.00	5.00
5.00	6.00	6.00	6.00	6.00	6.00
6.00	6.00	5.00	5.00	6.00	6.00
5.00	5.00	5.00	5.00	5.00	6.00
5.00	5.00	5.00	5.00	6.00	6.00
6.00	6.00	5.00	6.00	6.00	5.00
5.00	6.00	5.00	5.00	6.00	6.00
6.00	5.00	5.00	5.00	5.00	6.00
6.00	6.00	6.00	5.00	5.00	4.00
6.00	6.00	5.00	6.00	6.00	6.00
5.00	6.00	6.00	5.00	5.00	6.00
6.00	5.00	5.00	6.00	6.00	5.00
5.00	5.00	5.00	5.00	5.00	6.00
6.00	6.00	6.00	6.00	6.00	5.00
6.00	5.00	5.00	5.00	5.00	5.00
5.00	5.00	5.00	6.00	6.00	5.00
5.00	6.00	5.00	6.00	5.00	5.00
5.00	6.00	5.00	5.00	6.00	6.00

5.00	5.00	5.00	6.00	5.00	5.00
6.00	5.00	6.00	5.00	5.00	6.00

Panel L

>23–40, ♂	>23–40, ♀	41–60, ♂	41–60, ♀	60+, ♂	60+, ♀
5.00	5.00	5.00	6.00	6.00	5.00
5.00	5.00	6.00	5.00	6.00	6.00
6.00	6.00	5.00	5.00	5.00	5.00
5.00	6.00	5.00	5.00	5.00	6.00
6.00	5.00	6.00	6.00	5.00	5.00
6.00	6.00	6.00	6.00	5.00	6.00
5.00	6.00	5.00	6.00	6.00	5.00
6.00	6.00	5.00	6.00	5.00	5.00
6.00	6.00	5.00	6.00	5.00	5.00
5.00	6.00	5.00	5.00	5.00	5.00
6.00	6.00	5.00	6.00	5.00	6.00
6.00	6.00	5.00	6.00	5.00	5.00
5.00	6.00	6.00	4.00	6.00	6.00
5.00	6.00	5.00	6.00		5.00
6.00	5.00	6.00	5.00		6.00
6.00	5.00	5.00	6.00		
6.00	6.00	5.00	5.00		
6.00	5.00	6.00	6.00		
6.00	5.00	5.00	5.00		
7.00	5.00	5.00	6.00		
5.00	5.00	6.00	5.00		
6.00	6.00	6.00	6.00		
6.00	6.00	5.00	5.00		
5.00	6.00	5.00	5.00		
6.00	5.00	5.00	5.00		
6.00	6.00	6.00	6.00		
5.00	5.00	5.00			
5.00	6.00	5.00			
6.00	5.00	5.00			
6.00	6.00	6.00			
6.00	6.00	6.00			
6.00	6.00				
6.00	6.00				
5.00	5.00				
5.00	6.00				
6.00	6.00				
6.00	6.00				
5.00	5.00				
6.00	5.00				
6.00	6.00				
6.00	5.00				
5.00	5.00				

6.00	5.00				
6.00	5.00				
6.00	6.00				
5.00	5.00				
	5.00				
	5.00				
	6.00				

Panel M

Riverine, ♂	Riverine, ♀	Estuarine, ♂	Estuarine, ♀	Marine, ♂	Marine, ♀
3.00	3.00	4.00	4.00	4.00	3.00
3.00	3.00	4.00	4.00	3.00	4.00
4.00	4.00	4.00	4.00	3.00	3.00
3.00	4.00	3.00	3.00	4.00	4.00
4.00	3.00	3.00	4.00	3.00	3.00
4.00	4.00	4.00	4.00	3.00	4.00
3.00	4.00	4.00	4.00	4.00	3.00
4.00	4.00	3.00	3.00	4.00	4.00
4.00	4.00	4.00	3.00	3.00	3.00
3.00	4.00	4.00	4.00	3.00	3.00
4.00	4.00	4.00	3.00	3.00	3.00
4.00	4.00	3.00	4.00	4.00	4.00
3.00	4.00	4.00	3.00	3.00	3.00
3.00	4.00	3.00	3.00	4.00	4.00
4.00	3.00	3.00	3.00	4.00	3.00
4.00	3.00	4.00	4.00	3.00	4.00
4.00	4.00	3.00	3.00	3.00	4.00
4.00	3.00	4.00	3.00	3.00	3.00
4.00	3.00	4.00	3.00	3.00	4.00
5.00	3.00	4.00	4.00	4.00	3.00
3.00	4.00	3.00	3.00	4.00	4.00
4.00	4.00	3.00	4.00	3.00	3.00
4.00	4.00	3.00	4.00	3.00	4.00
3.00	4.00	3.00	4.00	4.00	3.00
4.00	3.00	3.00	4.00	3.00	4.00
4.00	4.00	3.00	3.00	3.00	3.00
3.00	3.00	3.00	4.00	3.00	3.00
3.00	4.00	3.00	4.00	3.00	4.00
4.00	3.00	4.00	3.00	3.00	3.00
4.00	4.00	3.00	4.00	4.00	4.00
3.67	3.63	3.47	3.57	3.37	3.47
0.54	0.48	0.50	0.50	0.48	0.50
3.00	3.00	3.00	3.00	3.00	3.00
5.00	4.00	4.00	4.00	4.00	4.00

	3.65		3.52		3.42
	0.51		0.50		0.49
					3.53
					0.51

Panel N

Pre-spawning, ♂	Spawning, ♂	Post-spawning, ♂	Pre-spawning, ♀	Spawning, ♀	Post-spawning, ♀
4.00	3.00	3.00	4.00	3.00	4.00
4.00	3.00	4.00	4.00	3.00	4.00
3.00	4.00	4.00	4.00	4.00	4.00
3.00	3.00	3.00	4.00	4.00	4.00
4.00	4.00	4.00	3.00	3.00	3.00
4.00	4.00	4.00	3.00	4.00	4.00
4.00	3.00	3.00	4.00	4.00	3.00
4.00	4.00	3.00	3.00	4.00	4.00
4.00	4.00	4.00	3.00	4.00	3.00
5.00	3.00	4.00	3.00	4.00	4.00
4.00	4.00	3.00	3.00	4.00	3.00
3.00	4.00	3.00	4.00	4.00	4.00
4.00	4.00	3.00	3.00	4.00	4.00
3.00	3.00	3.00	3.00	3.00	4.00
3.00	3.00	3.00	3.00	4.00	4.00
4.00	4.00	3.00	4.00	4.00	3.00
3.00	4.00	3.00	3.00	4.00	4.00
4.00	3.00	3.00	3.00	3.00	4.00
4.00	4.00	4.00	3.00	3.00	3.00
4.00	4.00	3.00	4.00	4.00	4.00
3.00	4.00	4.00	3.00	3.00	4.00
4.00	3.00	3.00	4.00	4.00	3.00
3.00	3.00	3.00	3.00	3.00	4.00
4.00	4.00	4.00	4.00	4.00	3.00
4.00	3.00	3.00	3.00	3.00	4.00
3.00	3.00	3.00	4.00	4.00	3.00
3.00	4.00	3.00	4.00	3.00	3.00
3.00	4.00	3.00	3.00	4.00	4.00
3.00	3.00	3.00	4.00	3.00	3.00
4.00	3.00	4.00	3.00	3.00	4.00

Panel O

>23–40, ♂	>23–40, ♀	41–60, ♂	41–60, ♀	60+, ♂	60+, ♀
3.00	3.00	3.00	4.00	4.00	3.00
3.00	3.00	4.00	3.00	4.00	4.00
4.00	4.00	3.00	3.00	3.00	3.00
3.00	4.00	3.00	3.00	3.00	4.00
4.00	3.00	4.00	4.00	3.00	3.00

4.00	4.00	3.00	4.00	3.00	4.00
3.00	4.00	3.00	4.00	4.00	3.00
4.00	4.00	3.00	4.00	3.00	4.00
4.00	4.00	3.00	4.00	3.00	3.00
3.00	4.00	3.00	3.00	3.00	3.00
4.00	4.00	3.00	4.00	3.00	4.00
4.00	4.00	3.00	4.00	3.00	3.00
3.00	4.00	4.00	3.00	4.00	4.00
3.00	4.00	3.00	4.00		3.00
4.00	3.00	4.00	3.00		4.00
4.00	3.00	3.00	4.00		
4.00	4.00	3.00	3.00		
4.00	3.00	4.00	4.00		
4.00	3.00	3.00	3.00		
5.00	3.00	3.00	4.00		
3.00	4.00	4.00	3.00		
4.00	4.00	4.00	4.00		
4.00	4.00	3.00	3.00		
3.00	4.00	3.00	3.00		
4.00	3.00	3.00	3.00		
4.00	4.00	4.00	4.00		
3.00	3.00	3.00			
3.00	4.00	3.00			
4.00	3.00	3.00			
4.00	4.00	4.00			
4.00	4.00	4.00			
4.00	4.00				
4.00	4.00				
3.00	3.00				
3.00	4.00				
4.00	4.00				
4.00	4.00				
3.00	3.00				
4.00	3.00				
4.00	4.00				
4.00	3.00				
3.00	3.00				
4.00	3.00				
4.00	3.00				
4.00	4.00				
3.00	3.00				
	3.00				
	3.00				
	4.00				

Panel P

Riverine, ♂	Riverine, ♀	Estuarine, ♂	Estuarine, ♀	Marine, ♂	Marine, ♀

11.78	11.78	18.85	18.85	18.85	11.78
11.78	11.78	18.85	18.85	11.78	18.85
18.85	18.85	18.85	18.85	11.78	11.78
11.78	18.85	11.78	11.78	18.85	18.85
18.85	11.78	11.78	18.85	11.78	11.78
18.85	18.85	18.85	18.85	11.78	18.85
11.78	18.85	18.85	18.85	18.85	11.78
18.85	18.85	11.78	11.78	18.85	18.85
18.85	18.85	18.85	11.78	11.78	11.78
11.78	18.85	18.85	18.85	11.78	11.78
18.85	18.85	18.85	11.78	11.78	11.78
18.85	18.85	11.78	18.85	18.85	18.85
11.78	18.85	18.85	11.78	11.78	11.78
11.78	18.85	11.78	11.78	18.85	18.85
18.85	11.78	11.78	11.78	18.85	11.78
18.85	11.78	18.85	18.85	11.78	18.85
18.85	18.85	11.78	11.78	11.78	18.85
18.85	11.78	18.85	11.78	11.78	11.78
18.85	11.78	18.85	11.78	11.78	18.85
27.49	11.78	18.85	18.85	18.85	11.78
11.78	15.71	11.78	11.78	18.85	18.85
18.85	18.85	14.14	18.85	11.78	11.78
18.85	18.85	11.78	18.85	11.78	18.85
11.78	18.85	11.78	18.85	18.85	11.78
18.85	11.78	11.78	18.85	11.78	15.71
18.85	18.85	11.78	11.78	11.78	11.78
11.78	11.78	11.78	18.85	11.78	11.78
11.78	18.85	11.78	18.85	11.78	18.85
18.85	11.78	18.85	9.42	11.78	11.78
18.85	18.85	11.78	18.85	18.85	18.85

Panel Q

Pre-spawning, ♂	Spawning, ♂	Post-spawning, ♂	Pre-spawning, ♀	Spawning, ♀	Post-spawning, ♀
18.85	11.78	11.78	18.85	11.78	15.71

18.85	11.78	18.85	18.85	11.78	18.85
11.78	18.85	18.85	18.85	18.85	18.85
11.78	11.78	11.78	18.85	18.85	18.85
18.85	18.85	18.85	11.78	11.78	11.78
18.85	18.85	18.85	11.78	18.85	18.85
18.85	11.78	11.78	18.85	18.85	11.78
18.85	18.85	11.78	11.78	18.85	18.85
18.85	18.85	18.85	11.78	18.85	11.78
27.49	11.78	18.85	11.78	18.85	18.85
18.85	18.85	11.78	11.78	18.85	11.78
11.78	18.85	14.14	18.85	18.85	18.85
18.85	18.85	11.78	11.78	18.85	18.85
11.78	11.78	11.78	11.78	11.78	18.85
11.78	11.78	11.78	11.78	18.85	18.85
18.85	18.85	11.78	18.85	18.85	11.78
11.78	18.85	11.78	11.78	18.85	18.85
18.85	11.78	11.78	11.78	11.78	18.85
18.85	18.85	18.85	11.78	11.78	9.42
18.85	18.85	11.78	18.85	18.85	18.85
11.78	18.85	18.85	11.78	11.78	18.85
18.85	11.78	11.78	18.85	18.85	11.78
11.78	11.78	11.78	11.78	11.78	18.85
18.85	18.85	18.85	18.85	18.85	11.78
18.85	11.78	11.78	11.78	11.78	15.71
11.78	11.78	11.78	18.85	18.85	11.78
11.78	18.85	11.78	18.85	11.78	11.78
11.78	18.85	11.78	11.78	18.85	18.85
11.78	11.78	11.78	18.85	11.78	11.78
18.85	11.78	18.85	11.78	11.78	18.85

Panel R

>23–40, ♂	>23–40, ♀	41–60, ♂	41–60, ♀	60+, ♂	60+, ♀
11.78	11.78	11.78	18.85	18.85	11.78
11.78	11.78	18.85	11.78	18.85	18.85
18.85	18.85	11.78	11.78	11.78	11.78
11.78	18.85	11.78	11.78	11.78	18.85
18.85	11.78	18.85	18.85	11.78	11.78
18.85	18.85	14.14	18.85	11.78	18.85
11.78	18.85	11.78	18.85	18.85	11.78
18.85	18.85	11.78	18.85	11.78	15.71
18.85	18.85	11.78	18.85	11.78	11.78
11.78	18.85	11.78	11.78	11.78	11.78
18.85	18.85	11.78	18.85	11.78	18.85
18.85	18.85	11.78	18.85	11.78	11.78
11.78	18.85	18.85	9.42	18.85	18.85
11.78	18.85	11.78	18.85		11.78
18.85	11.78	18.85	11.78		18.85

18.85	11.78	11.78	18.85		
18.85	18.85	11.78	11.78		
18.85	11.78	18.85	18.85		
18.85	11.78	11.78	11.78		
27.49	11.78	11.78	18.85		
11.78	15.71	18.85	11.78		
18.85	18.85	18.85	18.85		
18.85	18.85	11.78	11.78		
11.78	18.85	11.78	11.78		
18.85	11.78	11.78	11.78		
18.85	18.85	18.85	18.85		
11.78	11.78	11.78			
11.78	18.85	11.78			
18.85	11.78	11.78			
18.85	18.85	18.85			
18.85	18.85	18.85			
18.85	18.85				
18.85	18.85				
11.78	11.78				
11.78	18.85				
18.85	18.85				
18.85	18.85				
11.78	11.78				
18.85	11.78				
18.85	18.85				
18.85	11.78				
11.78	11.78				
18.85	11.78				
18.85	11.78				
18.85	18.85				
11.78	11.78				
	11.78				
	11.78				
	18.85				

Riverine, both	Estuarine, both	Marine, both	All, ♂	All, ♀
13.00	14.00	15.00	13.00	13.00
12.00	15.00	12.00	12.00	13.00
15.00	15.00	13.00	15.00	15.00
13.00	12.00	14.00	13.00	14.00
14.00	12.00	13.00	14.00	13.00
15.00	15.00	13.00	15.00	15.00
12.00	15.00	15.00	12.00	14.00
15.00	13.00	14.00	15.00	15.00
15.00	14.00	13.00	15.00	15.00
13.00	15.00	13.00	13.00	14.00
14.00	15.00	12.00	14.00	15.00
15.00	12.00	15.00	15.00	15.00
13.00	15.00	12.00	13.00	14.00
12.00	12.00	15.00	12.00	15.00
14.00	13.00	15.00	14.00	13.00
14.00	14.00	13.00	14.00	13.00
15.00	13.00	13.00	15.00	14.00
15.00	15.00	12.00	15.00	13.00
14.00	15.00	13.00	14.00	13.00
16.00	14.00	15.00	16.00	12.00
13.00	12.00	14.00	13.00	12.00
14.00	14.00	12.00	14.00	14.00
15.00	13.00	13.00	15.00	14.00
13.00	12.00	15.00	13.00	16.00
14.00	13.00	12.00	14.00	12.00
15.00	13.00	13.00	15.00	14.00
12.00	12.00	12.00	12.00	12.00
13.00	12.00	13.00	13.00	15.00
14.00	15.00	13.00	14.00	13.00
14.00	12.00	15.00	14.00	14.00
13.00	14.00	13.00	14.00	14.00
13.00	14.00	14.00	15.00	14.00
15.00	15.00	13.00	15.00	15.00
14.00	12.00	15.00	12.00	12.00
13.00	14.00	13.00	12.00	14.00
15.00	14.00	15.00	15.00	14.00
14.00	15.00	13.00	15.00	15.00
15.00	12.00	15.00	13.00	12.00
15.00	13.00	12.00	14.00	13.00
14.00	14.00	12.00	15.00	14.00
15.00	14.00	13.00	15.00	14.00
15.00	14.00	14.00	12.00	14.00
14.00	12.00	13.00	15.00	12.00
15.00	12.00	14.00	12.00	12.00

13.00	13.00	12.00	13.00	13.00
13.00	14.00	14.00	14.00	14.00
14.00	13.00	14.00	13.00	13.00
13.00	13.00	13.00	15.00	13.00
13.00	12.00	14.00	15.00	12.00
12.00	14.00	13.00	14.00	14.00
12.00	12.00	15.00	12.00	12.00
14.00	14.00	13.00	14.00	14.00
14.00	14.00	15.00	13.00	14.00
16.00	15.00	13.00	12.00	15.00
12.00	15.00	12.00	13.00	15.00
14.00	15.00	12.00	13.00	15.00
12.00	14.00	13.00	12.00	14.00
15.00	14.00	14.00	12.00	14.00
13.00	12.00	13.00	15.00	12.00
14.00	14.00	15.00	12.00	14.00
			15.00	13.00
			12.00	14.00
			13.00	13.00
			14.00	15.00
			13.00	13.00
			13.00	15.00
			15.00	13.00
			14.00	15.00
			13.00	12.00
			13.00	12.00
			12.00	13.00
			15.00	14.00
			12.00	13.00
			15.00	14.00
			15.00	12.00
			13.00	14.00
			13.00	14.00
			12.00	13.00
			13.00	14.00
			15.00	13.00
			14.00	15.00
			12.00	13.00
			13.00	15.00
			15.00	13.00
			12.00	12.00
			13.00	12.00
			12.00	13.00
			13.00	14.00
			13.00	13.00
			15.00	15.00

Riverine, both	Estuarine, both	Marine, both	All, ♂	All, ♀
8.67	9.33	10.00	8.67	8.67
8.00	10.00	8.00	8.00	8.67
10.00	10.00	8.67	10.00	10.00
8.67	8.00	9.33	8.67	9.33
9.33	8.00	8.67	9.33	8.67
10.00	10.00	8.67	10.00	10.00
8.00	10.00	10.00	8.00	9.33

10.00	8.67	9.33	10.00	10.00
10.00	9.33	8.67	10.00	10.00
8.67	10.00	8.67	8.67	9.33
9.33	10.00	8.00	9.33	10.00
10.00	8.00	10.00	10.00	10.00
8.67	10.00	8.00	8.67	9.33
8.00	8.00	10.00	8.00	10.00
9.33	8.67	10.00	9.33	8.67
9.33	9.33	8.67	9.33	8.67
10.00	8.67	8.67	10.00	9.33
10.00	10.00	8.00	10.00	8.67
9.33	10.00	8.67	9.33	8.67
10.67	9.33	10.00	10.67	8.00
8.67	8.00	9.33	8.67	8.00
9.33	9.33	8.00	9.33	9.33
10.00	8.67	8.67	10.00	9.33
8.67	8.00	10.00	8.67	10.67
9.33	8.67	8.00	9.33	8.00
10.00	8.67	8.67	10.00	9.33
8.00	8.00	8.00	8.00	8.00
8.67	8.00	8.67	8.67	10.00
9.33	10.00	8.67	9.33	8.67
9.33	8.00	10.00	9.33	9.33
8.67	9.33	8.67	9.33	9.33
8.67	9.33	9.33	10.00	9.33
10.00	10.00	8.67	10.00	10.00
9.33	8.00	10.00	8.00	8.00
8.67	9.33	8.67	8.00	9.33
10.00	9.33	10.00	10.00	9.33
9.33	10.00	8.67	10.00	10.00
10.00	8.00	10.00	8.67	8.00
10.00	8.67	8.00	9.33	8.67
9.33	9.33	8.00	10.00	9.33
10.00	9.33	8.67	10.00	9.33
10.00	9.33	9.33	8.00	9.33
9.33	8.00	8.67	10.00	8.00
10.00	8.00	9.33	8.00	8.00
8.67	8.67	8.00	8.67	8.67
8.67	9.33	9.33	9.33	9.33
9.33	8.67	9.33	8.67	8.67
8.67	8.67	8.67	10.00	8.67
8.67	8.00	9.33	10.00	8.00
8.00	9.33	8.67	9.33	9.33
8.00	8.00	10.00	8.00	8.00
9.33	9.33	8.67	9.33	9.33
9.33	9.33	10.00	8.67	9.33
10.67	10.00	8.67	8.00	10.00

8.00	10.00	8.00	8.67	10.00
9.33	10.00	8.00	8.67	10.00
8.00	9.33	8.67	8.00	9.33
10.00	9.33	9.33	8.00	9.33
8.67	8.00	8.67	10.00	8.00
9.33	9.33	10.00	8.00	9.33
			10.00	8.67
			8.00	9.33
			8.67	8.67
			9.33	10.00
			8.67	8.67
			8.67	10.00
			10.00	8.67
			9.33	10.00
			8.67	8.00
			8.67	8.00
			8.00	8.67
			10.00	9.33
			8.00	8.67
			10.00	9.33
			10.00	8.00
			8.67	9.33
			8.67	9.33
			8.00	8.67
			8.67	9.33
			10.00	8.67
			9.33	10.00
			8.00	8.67
			8.67	10.00
			10.00	8.67
			8.00	8.00
			8.67	8.00
			8.00	8.67
			8.67	9.33
			8.67	8.67
			10.00	10.00

Riverine, both	Estuarine, both	Marine, both	All, ♂	All, ♀
88.48	102.62	117.80	88.48	88.48
75.39	117.80	75.39	75.39	88.48
117.80	117.80	88.48	117.80	117.80
88.48	75.39	102.62	88.48	102.62
102.62	75.39	88.48	102.62	88.48
117.80	117.80	88.48	117.80	117.80
75.39	117.80	117.80	75.39	102.62
117.80	88.48	102.62	117.80	117.80
117.80	102.62	88.48	117.80	117.80
88.48	117.80	88.48	88.48	102.62
102.62	117.80	75.39	102.62	117.80
117.80	75.39	117.80	117.80	117.80
88.48	117.80	75.39	88.48	102.62
75.39	75.39	117.80	75.39	117.80
102.62	88.48	117.80	102.62	88.48
102.62	102.62	88.48	102.62	88.48
117.80	88.48	88.48	117.80	102.62

117.80	117.80	75.39	117.80	88.48
102.62	117.80	88.48	102.62	88.48
134.03	102.62	117.80	134.03	75.39
88.48	75.39	102.62	88.48	75.39
102.62	102.62	75.39	102.62	102.62
117.80	88.48	88.48	117.80	102.62
88.48	75.39	117.80	88.48	134.03
102.62	88.48	75.39	102.62	75.39
117.80	88.48	88.48	117.80	102.62
75.39	75.39	75.39	75.39	75.39
88.48	75.39	88.48	88.48	117.80
102.62	117.80	88.48	102.62	88.48
102.62	75.39	117.80	102.62	102.62
88.48	102.62	88.48	102.62	102.62
88.48	102.62	102.62	117.80	102.62
117.80	117.80	88.48	117.80	117.80
102.62	75.39	117.80	75.39	75.39
88.48	102.62	88.48	75.39	102.62
117.80	102.62	117.80	117.80	102.62
102.62	117.80	88.48	117.80	117.80
117.80	75.39	117.80	88.48	75.39
117.80	88.48	75.39	102.62	88.48
102.62	102.62	75.39	117.80	102.62
117.80	102.62	88.48	117.80	102.62
117.80	102.62	102.62	75.39	102.62
102.62	75.39	88.48	117.80	75.39
117.80	75.39	102.62	75.39	75.39
88.48	88.48	75.39	88.48	88.48
88.48	102.62	102.62	102.62	102.62
102.62	88.48	102.62	88.48	88.48
88.48	88.48	88.48	117.80	88.48
88.48	75.39	102.62	117.80	75.39
75.39	102.62	88.48	102.62	102.62
75.39	75.39	117.80	75.39	75.39
102.62	102.62	88.48	102.62	102.62
102.62	102.62	117.80	88.48	102.62
134.03	117.80	88.48	75.39	117.80
75.39	117.80	75.39	88.48	117.80
102.62	117.80	75.39	88.48	117.80
75.39	102.62	88.48	75.39	102.62
117.80	102.62	102.62	75.39	102.62
88.48	75.39	88.48	117.80	75.39
102.62	102.62	117.80	75.39	102.62
			117.80	88.48
			75.39	102.62
			88.48	88.48
			102.62	117.80

			88.48	88.48
			88.48	117.80
			117.80	88.48
			102.62	117.80
			88.48	75.39
			88.48	75.39
			75.39	88.48
			117.80	102.62
			75.39	88.48
			117.80	102.62
			117.80	75.39
			88.48	102.62
			88.48	102.62
			75.39	88.48
			88.48	102.62
			117.80	88.48
			102.62	117.80
			75.39	88.48
			88.48	117.80
			117.80	88.48
			75.39	75.39
			88.48	75.39
			75.39	88.48
			88.48	102.62
			88.48	88.48
			117.80	117.80

Riverine, both	Estuarine, both	Marine, both	All, ♂	All, ♀
5.00	6.00	6.00	5.00	5.00
5.00	6.00	5.00	5.00	5.00
6.00	6.00	5.00	6.00	6.00
5.00	5.00	6.00	5.00	6.00
6.00	5.00	5.00	6.00	5.00
6.00	6.00	5.00	6.00	6.00
5.00	6.00	6.00	5.00	6.00
6.00	5.00	6.00	6.00	6.00
6.00	6.00	5.00	6.00	6.00
5.00	6.00	5.00	5.00	6.00
6.00	6.00	5.00	6.00	6.00
6.00	5.00	6.00	6.00	6.00
5.00	6.00	5.00	5.00	6.00
5.00	5.00	6.00	5.00	6.00
6.00	5.00	6.00	6.00	5.00
6.00	6.00	5.00	6.00	5.00
6.00	5.00	5.00	6.00	6.00
6.00	6.00	5.00	6.00	5.00
6.00	6.00	5.00	6.00	5.00
7.00	6.00	6.00	7.00	5.00
5.00	5.00	6.00	5.00	5.00
6.00	6.00	5.00	6.00	6.00
6.00	5.00	5.00	6.00	6.00
5.00	5.00	6.00	5.00	6.00
6.00	5.00	5.00	6.00	5.00
6.00	5.00	5.00	6.00	6.00
5.00	5.00	5.00	5.00	5.00

5.00	5.00	5.00	5.00	6.00
6.00	6.00	5.00	6.00	5.00
6.00	5.00	6.00	6.00	6.00
5.00	6.00	5.00	6.00	6.00
5.00	6.00	6.00	6.00	6.00
6.00	6.00	5.00	6.00	6.00
6.00	5.00	6.00	5.00	5.00
5.00	6.00	5.00	5.00	6.00
6.00	6.00	6.00	6.00	6.00
6.00	6.00	5.00	6.00	6.00
6.00	5.00	6.00	5.00	5.00
6.00	5.00	5.00	6.00	5.00
6.00	6.00	5.00	6.00	6.00
6.00	5.00	5.00	6.00	5.00
6.00	6.00	6.00	5.00	6.00
6.00	5.00	5.00	6.00	5.00
6.00	5.00	6.00	5.00	5.00
5.00	5.00	5.00	5.00	5.00
5.00	6.00	6.00	6.00	6.00
6.00	5.00	6.00	5.00	5.00
5.00	5.00	5.00	6.00	5.00
5.00	5.00	6.00	6.00	5.00
5.00	6.00	5.00	6.00	6.00
5.00	5.00	6.00	5.00	5.00
6.00	6.00	5.00	6.00	6.00
6.00	6.00	6.00	5.00	6.00
6.00	6.00	5.00	5.00	6.00
5.00	6.00	5.00	5.00	6.00
6.00	5.00	5.00	5.00	5.00
5.00	6.00	5.00	5.00	6.00
6.00	6.00	6.00	5.00	6.00
5.00	4.00	5.00	6.00	4.00
6.00	6.00	6.00	5.00	6.00
			6.00	5.00
			5.00	6.00
			5.00	5.00
			6.00	6.00
			5.00	5.00
			5.00	6.00
			6.00	5.00
			6.00	6.00
			5.00	5.00
			5.00	5.00
			5.00	5.00
			6.00	6.00
			5.00	5.00
			6.00	6.00

			6.00	5.00
			5.00	6.00
			5.00	6.00
			5.00	5.00
			5.00	6.00
			6.00	5.00
			6.00	6.00
			5.00	5.00
			5.00	6.00
			6.00	5.00
			5.00	5.00
			5.00	5.00
			5.00	5.00
			5.00	6.00
			5.00	5.00
			6.00	6.00

Riverine, both	Estuarine, both	Marine, both	All, ♂	All, ♀
3.00	4.00	4.00	3.00	3.00
3.00	4.00	3.00	3.00	3.00
4.00	4.00	3.00	4.00	4.00
3.00	3.00	4.00	3.00	4.00
4.00	3.00	3.00	4.00	3.00
4.00	4.00	3.00	4.00	4.00
3.00	4.00	4.00	3.00	4.00
4.00	3.00	4.00	4.00	4.00
4.00	4.00	3.00	4.00	4.00
3.00	4.00	3.00	3.00	4.00
4.00	4.00	3.00	4.00	4.00
4.00	3.00	4.00	4.00	4.00
3.00	4.00	3.00	3.00	4.00
3.00	3.00	4.00	3.00	4.00
4.00	3.00	4.00	4.00	3.00
4.00	4.00	3.00	4.00	3.00
4.00	3.00	3.00	4.00	4.00
4.00	4.00	3.00	4.00	3.00
4.00	4.00	3.00	4.00	3.00
5.00	4.00	4.00	5.00	3.00
3.00	3.00	4.00	3.00	4.00
4.00	3.00	3.00	4.00	4.00
4.00	3.00	3.00	4.00	4.00
3.00	3.00	4.00	3.00	4.00
4.00	3.00	3.00	4.00	3.00
4.00	3.00	3.00	4.00	4.00
3.00	3.00	3.00	3.00	3.00
3.00	3.00	3.00	3.00	4.00
4.00	4.00	3.00	4.00	3.00
4.00	3.00	4.00	4.00	4.00
3.00	4.00	3.00	4.00	4.00
3.00	4.00	4.00	4.00	4.00
4.00	4.00	3.00	4.00	4.00
4.00	3.00	4.00	3.00	3.00
3.00	4.00	3.00	3.00	4.00
4.00	4.00	4.00	4.00	4.00
4.00	4.00	3.00	4.00	4.00

4.00	3.00	4.00	3.00	3.00
4.00	3.00	3.00	4.00	3.00
4.00	4.00	3.00	4.00	4.00
4.00	3.00	3.00	4.00	3.00
4.00	4.00	4.00	3.00	4.00
4.00	3.00	3.00	4.00	3.00
4.00	3.00	4.00	3.00	3.00
3.00	3.00	3.00	3.00	3.00
3.00	4.00	4.00	4.00	4.00
4.00	3.00	4.00	3.00	3.00
3.00	3.00	3.00	4.00	3.00
3.00	3.00	4.00	4.00	3.00
3.00	4.00	3.00	4.00	4.00
4.00	3.00	4.00	3.00	3.00
4.00	4.00	3.00	3.00	4.00
4.00	4.00	4.00	3.00	4.00
4.00	4.00	3.00	3.00	4.00
3.00	4.00	4.00	3.00	4.00
4.00	3.00	3.00	3.00	3.00
3.00	4.00	3.00	3.00	4.00
4.00	4.00	4.00	3.00	4.00
3.00	3.00	3.00	4.00	3.00
4.00	4.00	4.00	3.00	4.00
			4.00	3.00
			3.00	4.00
			3.00	3.00
			4.00	4.00
			3.00	3.00
			3.00	4.00
			4.00	3.00
			4.00	4.00
			3.00	3.00
			3.00	3.00
			3.00	3.00
			4.00	4.00
			3.00	3.00
			4.00	4.00
			4.00	3.00
			3.00	4.00
			3.00	4.00
			3.00	3.00
			3.00	4.00
			4.00	3.00
			4.00	4.00
			3.00	3.00
			3.00	4.00
			4.00	3.00

			3.00	4.00
			3.00	3.00
			3.00	3.00
			3.00	4.00
			3.00	3.00
			4.00	4.00

Riverine, both	Estuarine, both	Marine, both	All, ♂	All, ♀

11.78	18.85	18.85	11.78	11.78
11.78	18.85	11.78	11.78	11.78
18.85	18.85	11.78	18.85	18.85
11.78	11.78	18.85	11.78	18.85
18.85	11.78	11.78	18.85	11.78
18.85	18.85	11.78	18.85	18.85
11.78	18.85	18.85	11.78	18.85
18.85	11.78	18.85	18.85	18.85
18.85	18.85	11.78	18.85	18.85
11.78	18.85	11.78	11.78	18.85
18.85	18.85	11.78	18.85	18.85
18.85	11.78	18.85	18.85	18.85
11.78	18.85	11.78	11.78	18.85
11.78	11.78	18.85	11.78	18.85
18.85	11.78	18.85	18.85	11.78
18.85	18.85	11.78	18.85	11.78
18.85	11.78	11.78	18.85	18.85
18.85	18.85	11.78	18.85	11.78
18.85	18.85	11.78	18.85	11.78
27.49	18.85	18.85	27.49	11.78
11.78	11.78	18.85	11.78	15.71
18.85	14.14	11.78	18.85	18.85
18.85	11.78	11.78	18.85	18.85
11.78	11.78	18.85	11.78	18.85
18.85	11.78	11.78	18.85	11.78
18.85	11.78	11.78	18.85	18.85
11.78	11.78	11.78	11.78	11.78
11.78	11.78	11.78	11.78	18.85
18.85	18.85	11.78	18.85	11.78
18.85	11.78	18.85	18.85	18.85
11.78	18.85	11.78	18.85	18.85
11.78	18.85	18.85	18.85	18.85
18.85	18.85	11.78	18.85	18.85
18.85	11.78	18.85	11.78	11.78
11.78	18.85	11.78	11.78	18.85
18.85	18.85	18.85	18.85	18.85
18.85	18.85	11.78	18.85	18.85
18.85	11.78	18.85	11.78	11.78
18.85	11.78	11.78	18.85	11.78
18.85	18.85	11.78	18.85	18.85
18.85	11.78	11.78	18.85	11.78
18.85	18.85	18.85	11.78	18.85
18.85	11.78	11.78	18.85	11.78
18.85	11.78	18.85	11.78	11.78
11.78	11.78	11.78	11.78	11.78
11.78	18.85	18.85	18.85	18.85
18.85	11.78	18.85	11.78	11.78

11.78	11.78	11.78	18.85	11.78
11.78	11.78	18.85	18.85	11.78
11.78	18.85	11.78	18.85	18.85
15.71	11.78	18.85	11.78	11.78
18.85	18.85	11.78	14.14	18.85
18.85	18.85	18.85	11.78	18.85
18.85	18.85	11.78	11.78	18.85
11.78	18.85	15.71	11.78	18.85
18.85	11.78	11.78	11.78	11.78
11.78	18.85	11.78	11.78	18.85
18.85	18.85	18.85	11.78	18.85
11.78	9.42	11.78	18.85	9.42
18.85	18.85	18.85	11.78	18.85
			18.85	11.78
			11.78	18.85
			11.78	11.78
			18.85	18.85
			11.78	11.78
			11.78	18.85
			18.85	11.78
			18.85	18.85
			11.78	11.78
			11.78	11.78
			11.78	11.78
			18.85	18.85
			11.78	11.78
			18.85	18.85
			18.85	11.78
			11.78	18.85
			11.78	18.85
			11.78	11.78
			11.78	18.85
			18.85	11.78
			18.85	18.85
			11.78	11.78
			11.78	18.85
			18.85	11.78
			11.78	15.71
			11.78	11.78
			11.78	11.78
			11.78	18.85
			11.78	11.78
			18.85	18.85

S5 Figure

Panel A

Riverine, ♂	Riverine, ♀	Estuarine, ♂	Estuarine, ♀	Marine, ♂	Marine, ♀	Riverine, both	Estuarine, both
16.00	18.00	20.00	14.00	23.00	18.00	16.00	20.00
10.00	17.00	19.00	16.00	19.00	18.00	10.00	19.00
22.00	20.00	18.00	15.00	12.00	20.00	22.00	18.00
16.00	18.00	20.00	14.00	23.00	18.00	16.00	20.00
10.00	17.00	19.00	16.00	19.00	18.00	10.00	19.00
22.00	20.00	18.00	15.00	12.00	20.00	22.00	18.00
11.00	21.00	22.00	13.00	13.00	19.00	11.00	22.00
16.00	18.00	20.00	14.00	13.00	18.00	16.00	20.00
10.00	17.00	19.00	16.00	19.00	18.00	10.00	19.00
22.00	20.00	18.00	15.00	12.00	20.00	22.00	18.00
11.00	21.00	22.00	13.00	13.00	19.00	11.00	22.00
8.00	21.00	15.00	13.00	15.00	19.00	8.00	15.00
19.00	24.00	12.00	13.00	10.00	17.00	19.00	12.00
19.00	17.00	13.00	18.00	14.00	20.00	19.00	13.00
20.00	19.00	13.00	15.00	9.00	23.00	20.00	13.00
20.00	22.00	11.00	15.00	9.00	17.00	20.00	11.00
9.00	23.00	20.00	14.00	11.00	15.00	9.00	20.00
25.00	10.00	19.00	11.00	14.00	15.00	25.00	19.00
19.00	19.00	12.00	8.00	15.00	19.00	19.00	12.00
9.00	24.00	10.00	16.00	11.00	12.00	9.00	10.00
11.00	11.00	11.00	23.00	9.00	9.00	11.00	11.00
10.00	14.00	11.00	18.00	13.00	18.00	10.00	11.00
18.00	20.00	8.00	8.00	10.00	11.00	18.00	8.00
12.00	11.00	24.00	10.00	14.00	19.00	12.00	24.00
17.00	15.00	21.00	19.00	13.00	17.00	17.00	21.00
25.00	12.00	19.00	15.00	17.00	13.00	25.00	19.00
9.00	14.00	13.00	14.00	15.00	10.00	9.00	13.00
14.00	10.00	13.00	13.00	11.00	23.00	14.00	13.00
19.00	10.00	14.00	18.00	21.00	15.00	19.00	14.00
12.00	25.00	8.00	8.00	24.00	18.00	12.00	8.00
16.00	10.00	20.00	19.00	15.00	22.00	16.00	20.00
16.00	20.00	21.00	12.00	15.00	9.00	16.00	21.00
14.00	12.00	8.00	13.00	13.00	18.00	14.00	8.00
15.00	13.00	23.00	23.00	10.00	15.00	15.00	23.00
12.00	17.00	12.00	17.00	16.00	20.00	12.00	12.00
11.00	9.00	14.00	10.00	13.00	15.00	11.00	14.00
24.00	13.00	13.00	20.00	9.00	20.00	24.00	13.00
						18.00	14.00
						17.00	16.00
						20.00	15.00
						21.00	13.00
						21.00	13.00
						24.00	13.00
						17.00	18.00

						19.00	15.00
						22.00	15.00
						23.00	14.00
						10.00	11.00
						19.00	8.00
						24.00	16.00
						11.00	23.00
						14.00	18.00
						20.00	8.00
						11.00	10.00
						15.00	19.00
						12.00	15.00
						14.00	14.00
						10.00	13.00
						10.00	18.00
						25.00	8.00
						10.00	19.00
						20.00	12.00
						12.00	13.00
						13.00	23.00
						17.00	17.00
						9.00	10.00
						13.00	20.00

Panel D

Riverine, ♂	Riverine, ♀	Estuarine, ♂	Estuarine, ♀	Marine, ♂	Marine, ♀	Riverine, both	Estuarine, both
17.00	20.00	25.00	15.00	26.00	22.00	17.00	25.00
10.00	19.00	22.00	16.00	21.00	21.00	10.00	22.00
24.00	21.00	21.00	10.00	13.00	24.00	24.00	21.00
12.00	22.00	25.00	14.00	14.00	22.00	12.00	25.00
9.00	22.00	17.00	14.00	16.00	21.00	9.00	17.00
21.00	26.00	13.00	14.00	22.00	19.00	21.00	13.00
20.00	18.00	16.00	19.00	14.00	24.00	20.00	16.00
21.00	19.00	16.00	16.00	9.00	27.00	21.00	16.00
22.00	23.00	13.00	17.00	9.00	19.00	22.00	13.00
9.00	26.00	25.00	14.00	21.00	18.00	9.00	25.00
26.00	11.00	22.00	12.00	15.00	18.00	26.00	22.00
20.00	20.00	14.00	9.00	27.00	21.00	20.00	14.00
10.00	25.00	11.00	18.00	11.00	14.00	10.00	11.00
11.00	12.00	14.00	23.00	9.00	11.00	11.00	14.00
10.00	15.00	13.00	18.00	14.00	21.00	10.00	13.00
20.00	27.00	9.00	9.00	21.00	12.00	20.00	9.00
12.00	22.00	27.00	10.00	25.00	22.00	12.00	27.00
19.00	15.00	26.00	21.00	13.00	21.00	19.00	26.00
26.00	13.00	22.00	26.00	18.00	15.00	26.00	22.00
10.00	16.00	15.00	16.00	17.00	12.00	10.00	15.00
15.00	11.00	15.00	14.00	21.00	15.00	15.00	15.00
21.00	10.00	17.00	19.00	22.00	18.00	21.00	17.00
13.00	27.00	10.00	9.00	27.00	22.00	13.00	10.00
18.00	11.00	23.00	21.00	17.00	25.00	18.00	23.00
27.00	21.00	25.00	23.00	15.00	9.00	27.00	25.00
26.00	13.00	9.00	25.00	24.00	19.00	26.00	9.00
27.00	23.00	27.00	24.00	16.00	18.00	27.00	27.00
14.00	24.00	14.00	19.00	17.00	13.00	14.00	14.00
12.00	9.00	17.00	11.00	14.00	18.00	12.00	17.00
27.00	14.00	14.00	21.00	9.00	12.00	27.00	14.00
						20.00	15.00
						19.00	16.00
						21.00	10.00
						22.00	14.00
						22.00	14.00
						26.00	14.00
						18.00	19.00
						19.00	16.00

						23.00	17.00
						26.00	14.00
						11.00	12.00
						20.00	9.00
						25.00	18.00
						12.00	23.00
						15.00	18.00
						27.00	9.00
						22.00	10.00
						15.00	21.00
						13.00	26.00
						16.00	16.00
						11.00	14.00
						10.00	19.00
						27.00	9.00
						11.00	21.00
						21.00	23.00
						13.00	25.00
						23.00	24.00
						24.00	19.00
						9.00	11.00
						14.00	21.00

Panel G

Riverine, ♂	Riverine, ♀	Estuarine, ♂	Estuarine, ♀	Marine, ♂	Marine, ♀	Riverine, both	Estuarine, both
2.78	2.41	2.85	2.98	2.99	2.28	2.78	2.85
2.89	2.99	2.94	2.73	2.86	2.83	2.89	2.94
3.38	2.84	3.17	2.70	3.33	2.73	3.38	3.17
2.64	2.62	2.83	2.88	3.01	3.04	2.64	2.83
2.57	2.59	2.78	3.38	2.60	2.84	2.57	2.78
2.90	2.65	2.98	2.78	2.85	2.55	2.90	2.98
2.98	2.83	2.60	3.50	3.33	2.61	2.98	2.60
3.37	3.07	2.85	2.55	2.66	2.83	3.37	2.85
2.57	2.88	2.64	3.48	3.41	2.50	2.57	2.64
3.40	2.52	3.12	2.91	2.60	2.96	3.40	3.12
2.61	3.19	2.87	2.83	3.45	3.02	2.61	2.87
3.49	2.58	3.36	3.05	2.55	3.04	3.49	3.36
2.82	3.16	2.97	3.47	2.82	2.52	2.82	2.97
3.10	3.45	3.31	2.90	2.66	3.16	3.10	3.31
3.47	3.34	3.00	2.61	2.51	2.79	3.47	3.00
2.77	2.67	2.81	2.63	2.66	3.44	2.77	2.81
3.04	2.75	3.20	3.40	2.60	3.22	3.04	3.20
2.85	2.73	2.93	2.77	3.01	3.34	2.85	2.93
3.36	3.45	2.81	2.85	3.39	2.51	3.36	2.81
2.62	2.52	3.10	3.39	2.84	2.92	2.62	3.10
3.32	3.42	3.34	3.12	2.54	2.97	3.32	3.34
2.85	3.30	2.53	3.28	2.61	2.71	2.85	2.53
3.16	3.14	2.94	2.89	2.68	3.31	3.16	2.94
3.21	3.14	2.94	2.79	2.85	3.25	3.21	2.94
3.39	3.47	2.89	2.82	2.81	2.63	3.39	2.89
3.08	2.60	3.27	2.71	3.25	2.61	3.08	3.27
3.22	3.31	3.06	3.27	3.50	3.31	3.22	3.06
2.50	2.73	3.15	2.92	3.09	3.20	2.50	3.15
3.41	2.63	2.95	2.51	2.61	3.26	3.41	2.95
2.85	2.95	2.63	3.06	2.95	2.88	2.85	2.63
						2.41	2.98
						2.99	2.73
						2.84	2.70
						2.62	2.88
						2.59	3.38
						2.65	2.78
						2.83	3.50
						3.07	2.55
						2.88	3.48

						2.52	2.91
						3.19	2.83
						2.58	3.05
						3.16	3.47
						3.45	2.90
						3.34	2.61
						2.67	2.63
						2.75	3.40
						2.73	2.77
						3.45	2.85
						2.52	3.39
						3.42	3.12
						3.30	3.28
						3.14	2.89
						3.14	2.79
						3.47	2.82
						2.60	2.71
						3.31	3.27
						2.73	2.92
						2.63	2.51
						2.95	3.06

Panel J

Riverine, ♂	Riverine, ♀	Estuarine, ♂	Estuarine, ♀	Marine, ♂	Marine, ♀	Riverine, both	Estuarine, both
0.77	0.82	0.79	0.85	0.82	0.84	0.77	0.79
0.77	0.81	0.78	0.83	0.77	0.85	0.77	0.78
0.80	0.76	0.81	0.83	0.81	0.85	0.80	0.81
0.83	0.76	0.78	0.81	0.81	0.84	0.83	0.78
0.76	0.83	0.84	0.85	0.81	0.84	0.76	0.84
0.78	0.76	0.81	0.82	0.80	0.84	0.78	0.81
0.85	0.85	0.80	0.81	0.83	0.84	0.85	0.80
0.78	0.79	0.80	0.82	0.82	0.84	0.78	0.80
0.82	0.85	0.79	0.85	0.80	0.85	0.82	0.79
0.77	0.84	0.84	0.84	0.81	0.81	0.77	0.84
0.78	0.80	0.76	0.78	0.80	0.82	0.78	0.76
0.79	0.79	0.77	0.84	0.77	0.85	0.79	0.77
0.80	0.78	0.85	0.77	0.82	0.81	0.80	0.85
0.78	0.77	0.79	0.81	0.84	0.84	0.78	0.79
0.83	0.85	0.81	0.78	0.78	0.79	0.83	0.81
0.79	0.81	0.81	0.80	0.81	0.77	0.79	0.81
0.79	0.81	0.79	0.79	0.81	0.76	0.79	0.79
0.82	0.83	0.80	0.76	0.84	0.77	0.82	0.80
0.80	0.82	0.78	0.82	0.78	0.78	0.80	0.78
0.82	0.85	0.85	0.76	0.82	0.78	0.82	0.85
0.81	0.85	0.84	0.79	0.82	0.77	0.81	0.84
0.84	0.82	0.77	0.81	0.79	0.76	0.84	0.77
0.81	0.82	0.81	0.84	0.80	0.77	0.81	0.81
0.77	0.82	0.83	0.78	0.85	0.76	0.77	0.83
0.85	0.77	0.76	0.83	0.83	0.77	0.85	0.76
0.81	0.78	0.79	0.83	0.82	0.78	0.81	0.79
0.83	0.78	0.82	0.79	0.85	0.83	0.83	0.82
0.82	0.78	0.77	0.80	0.83	0.80	0.82	0.77
0.82	0.85	0.82	0.83	0.80	0.77	0.82	0.82
0.78	0.84	0.77	0.83	0.77	0.76	0.78	0.77
						0.82	0.85
						0.81	0.83
						0.76	0.83
						0.76	0.81
						0.83	0.85
						0.76	0.82
						0.85	0.81
						0.79	0.82
						0.85	0.85
						0.84	0.84

						0.80	0.78
						0.79	0.84
						0.78	0.77
						0.77	0.81
						0.85	0.78
						0.81	0.80
						0.81	0.79
						0.83	0.76
						0.82	0.82
						0.85	0.76
						0.85	0.79
						0.82	0.81
						0.82	0.84
						0.82	0.78
						0.77	0.83
						0.78	0.83
						0.78	0.79
						0.78	0.80
						0.85	0.83
						0.84	0.83

Panel M

Riverine, ♂	Riverine, ♀	Estuarine, ♂	Estuarine, ♀	Marine, ♂	Marine, ♀	Riverine, both	Estuarine, both
1.24	1.20	1.12	1.11	0.99	1.09	1.24	1.12
0.87	1.17	1.03	1.17	1.25	1.14	0.87	1.03
0.94	1.28	1.07	1.18	1.23	1.36	0.94	1.07
1.09	1.12	1.11	1.14	0.96	1.13	1.09	1.11
1.23	1.11	1.19	1.12	0.99	1.19	1.23	1.19
0.87	1.12	1.07	1.16	1.29	1.15	0.87	1.07
1.00	1.19	1.05	1.15	1.25	1.24	1.00	1.05
1.02	1.19	1.05	1.11	0.96	1.19	1.02	1.05
1.32	1.15	1.12	1.12	0.98	1.19	1.32	1.12
0.86	1.19	1.11	1.12	1.31	1.17	0.86	1.11
0.92	0.97	1.00	1.10	1.22	1.33	0.92	1.00
1.03	0.85	1.40	0.98	0.90	1.00	1.03	1.40
1.29	1.18	1.20	1.02	1.00	0.90	1.29	1.20
0.87	0.94	1.04	1.15	1.05	1.11	0.87	1.04
0.98	1.03	1.06	1.20	1.19	1.37	0.98	1.06
1.04	0.88	1.07	0.94	0.95	0.91	1.04	1.07
1.28	1.23	1.11	1.09	0.98	1.29	1.28	1.11
0.92	1.01	1.06	1.08	1.36	1.08	0.92	1.06
0.95	1.00	1.03	1.16	1.18	1.31	0.95	1.03
1.08	0.92	1.03	0.99	0.97	0.92	1.08	1.03
1.30	1.29	1.19	1.04	1.00	0.91	1.30	1.19
1.38	1.00	1.02	1.15	1.28	1.00	1.38	1.02
1.25	1.00	1.07	1.18	1.21	1.37	1.25	1.07
1.00	0.88	1.01	0.91	1.00	1.20	1.00	1.01
1.37	1.29	1.08	1.10	1.01	1.19	1.37	1.08
0.90	1.02	1.04	1.18	1.32	1.03	0.90	1.04
1.34	1.27	1.16	1.04	1.03	1.13	1.34	1.16
0.91	1.00	1.02	1.13	1.35	1.06	0.91	1.02
0.97	1.03	1.08	1.18	1.21	1.25	0.97	1.08
1.03	0.90	1.39	0.97	0.91	1.29	1.03	1.39
						1.20	1.11
						1.17	1.17
						1.28	1.18
						1.12	1.14
						1.11	1.12
						1.12	1.16
						1.19	1.15
						1.19	1.11
						1.15	1.12
						1.19	1.12
						0.97	1.10

						0.85	0.98
						1.18	1.02
						0.94	1.15
						1.03	1.20
						0.88	0.94
						1.23	1.09
						1.01	1.08
						1.00	1.16
						0.92	0.99
						1.29	1.04
						1.00	1.15
						1.00	1.18
						0.88	0.91
						1.29	1.10
						1.02	1.18
						1.27	1.04
						1.00	1.13
						1.03	1.18
						0.90	0.97

Panel P

Riverine, ♂	Riverine, ♀	Estuarine, ♂	Estuarine, ♀	Marine, ♂	Marine, ♀	Riverine, both	Estuarine, both
178.00	193.00	141.00	158.00	190.00	201.00	178.00	141.00
151.00	192.00	163.00	159.00	194.00	192.00	151.00	163.00
182.00	206.00	163.00	170.00	191.00	217.00	182.00	163.00
136.00	208.00	145.00	145.00	191.00	192.00	136.00	145.00
169.00	186.00	133.00	131.00	186.00	214.00	169.00	133.00
141.00	205.00	135.00	146.00	199.00	212.00	141.00	135.00
152.00	194.00	166.00	139.00	187.00	195.00	152.00	166.00
177.00	196.00	160.00	151.00	194.00	203.00	177.00	160.00
165.00	186.00	157.00	160.00	185.00	208.00	165.00	157.00
147.00	212.00	132.00	146.00	185.00	190.00	147.00	132.00
150.00	138.00	156.00	126.00	168.00	159.00	150.00	156.00
197.00	158.00	143.00	154.00	148.00	166.00	197.00	143.00
160.00	179.00	161.00	166.00	178.00	192.00	160.00	161.00
193.00	193.00	157.00	189.00	172.00	150.00	193.00	157.00
181.00	160.00	157.00	182.00	172.00	148.00	181.00	157.00
180.00	187.00	187.00	183.00	151.00	159.00	180.00	187.00
191.00	196.00	196.00	170.00	178.00	157.00	191.00	196.00
186.00	168.00	174.00	159.00	160.00	162.00	186.00	174.00
188.00	167.00	185.00	185.00	192.00	174.00	188.00	185.00
182.00	180.00	183.00	184.00	150.00	129.00	182.00	183.00
171.00	172.00	171.00	160.00	141.00	129.00	171.00	171.00
183.00	178.00	187.00	187.00	141.00	148.00	183.00	187.00
161.00	169.00	163.00	165.00	140.00	140.00	161.00	163.00
180.00	167.00	168.00	167.00	135.00	143.00	180.00	168.00
166.00	158.00	170.00	166.00	142.00	127.00	166.00	170.00
163.00	167.00	161.00	168.00	126.00	130.00	163.00	161.00
169.00	157.00	166.00	165.00	131.00	144.00	169.00	166.00
161.00	166.00	170.00	157.00	128.00	131.00	161.00	170.00
160.00	156.00	158.00	165.00	130.00	144.00	160.00	158.00
148.00	154.00	170.00	149.00	155.00	127.00	148.00	170.00
						193.00	158.00
						192.00	159.00
						206.00	170.00
						208.00	145.00
						186.00	131.00
						205.00	146.00
						194.00	139.00
						196.00	151.00
						186.00	160.00
						212.00	146.00
						138.00	126.00
						158.00	154.00

						179.00	166.00
						193.00	189.00
						160.00	182.00
						187.00	183.00
						196.00	170.00
						168.00	159.00
						167.00	185.00
						180.00	184.00
						172.00	160.00
						178.00	187.00
						169.00	165.00
						167.00	167.00
						158.00	166.00
						167.00	168.00
						157.00	165.00
						166.00	157.00
						156.00	165.00
						154.00	149.00

Panel S

Riverine, ♂	Riverine, ♀	Estuarine, ♂	Estuarine, ♀	Marine, ♂	Marine, ♀	Riverine, both	Estuarine, both
0.15	0.12	0.14	0.14	0.12	0.14	0.15	0.14
0.10	0.11	0.15	0.14	0.14	0.14	0.10	0.15
0.08	0.14	0.15	0.13	0.14	0.13	0.08	0.15
0.14	0.12	0.15	0.14	0.11	0.14	0.14	0.15
0.12	0.12	0.13	0.14	0.14	0.14	0.12	0.13
0.13	0.14	0.15	0.13	0.15	0.13	0.13	0.15
0.09	0.13	0.12	0.14	0.12	0.13	0.09	0.12
0.12	0.14	0.14	0.14	0.15	0.14	0.12	0.14
0.09	0.13	0.14	0.14	0.14	0.13	0.09	0.14
0.08	0.14	0.14	0.15	0.13	0.15	0.08	0.14
0.14	0.08	0.12	0.12	0.13	0.14	0.14	0.12
0.09	0.13	0.14	0.08	0.09	0.08	0.09	0.14
0.11	0.14	0.13	0.14	0.15	0.09	0.11	0.13
0.07	0.10	0.14	0.11	0.12	0.12	0.07	0.14
0.12	0.09	0.14	0.15	0.15	0.07	0.12	0.14
0.08	0.08	0.12	0.14	0.15	0.08	0.08	0.12
0.14	0.12	0.13	0.14	0.08	0.08	0.14	0.13
0.12	0.11	0.14	0.14	0.09	0.11	0.12	0.14
0.09	0.11	0.15	0.14	0.08	0.12	0.09	0.15
0.12	0.09	0.13	0.14	0.13	0.14	0.12	0.13
0.15	0.12	0.15	0.12	0.12	0.08	0.15	0.15
0.12	0.14	0.13	0.09	0.10	0.09	0.12	0.13
0.14	0.12	0.13	0.12	0.14	0.10	0.14	0.13
0.15	0.12	0.15	0.14	0.08	0.07	0.15	0.15
0.12	0.14	0.12	0.15	0.14	0.10	0.12	0.12
0.11	0.10	0.12	0.10	0.13	0.11	0.11	0.12
0.15	0.09	0.08	0.08	0.09	0.11	0.15	0.08
0.13	0.15	0.14	0.10	0.09	0.13	0.13	0.14
0.14	0.09	0.15	0.09	0.11	0.14	0.14	0.15
0.14	0.12	0.13	0.07	0.08	0.14	0.14	0.13
						0.12	0.14
						0.11	0.14
						0.14	0.13
						0.12	0.14
						0.12	0.14
						0.14	0.13
						0.13	0.14
						0.14	0.14
						0.13	0.14
						0.14	0.15
						0.08	0.12
						0.13	0.08
						0.14	0.14

						0.10	0.11
						0.09	0.15
						0.08	0.14
						0.12	0.14
						0.11	0.14
						0.11	0.14
						0.09	0.14
						0.12	0.12
						0.14	0.09
						0.12	0.12
						0.12	0.14
						0.14	0.15
						0.10	0.10
						0.09	0.08
						0.15	0.10
						0.09	0.09
						0.12	0.07

Panel V

Riverine, ♂	Riverine, ♀	Estuarine, ♂	Estuarine, ♀	Marine, ♂	Marine, ♀	Riverine, both	Estuarine, both
2.82	3.78	4.67	4.96	4.47	4.00	2.82	4.67
3.50	4.91	4.52	3.34	4.77	3.89	3.50	4.52
5.09	3.78	3.85	5.50	3.51	4.41	5.09	3.85
5.03	5.70	2.94	2.53	4.94	5.50	5.03	2.94
4.85	5.43	3.30	5.04	4.57	3.75	4.85	3.30
4.27	3.71	3.94	5.23	4.82	3.87	4.27	3.94
4.75	5.53	4.68	4.75	3.47	5.15	4.75	4.68
3.48	4.64	5.23	2.63	4.61	5.34	3.48	5.23
2.69	3.84	3.71	5.25	4.17	5.76	2.69	3.71
5.02	5.12	3.31	3.98	2.96	5.18	5.02	3.31
3.66	5.09	2.35	5.09	4.57	2.55	3.66	2.35
4.29	2.53	3.17	4.54	5.12	3.60	4.29	3.17
3.64	3.06	4.52	3.52	2.76	3.39	3.64	4.52
3.16	4.45	3.91	3.75	5.02	5.20	3.16	3.91
4.16	4.53	4.74	4.41	2.68	2.82	4.16	4.74
3.38	5.21	4.93	3.71	3.82	4.21	3.38	4.93
4.97	2.99	2.47	4.27	3.94	3.33	4.97	2.47
3.62	4.53	4.91	3.00	4.67	4.27	3.62	4.91
3.09	4.66	3.48	3.29	3.27	3.24	3.09	3.48
3.29	5.23	3.72	2.90	3.14	2.69	3.29	3.72
3.50	2.52	2.67	2.55	2.81	5.26	3.50	2.67
2.86	2.81	4.70	3.19	4.59	4.71	2.86	4.70
4.45	2.78	3.87	3.56	5.29	4.39	4.45	3.87
2.38	4.66	5.22	4.83	5.25	4.52	2.38	5.22
2.75	3.57	4.76	3.10	2.94	4.96	2.75	4.76
3.39	4.32	2.79	2.44	2.93	4.45	3.39	2.79
2.69	5.02	2.48	4.04	4.35	4.87	2.69	2.48
3.61	3.24	3.87	2.43	4.79	3.95	3.61	3.87
4.35	2.39	4.51	4.97	3.01	4.71	4.35	4.51
2.67	3.00	2.35	4.22	3.99	3.28	2.67	2.35
						3.78	4.96
						4.91	3.34
						3.78	5.50
						5.70	2.53
						5.43	5.04
						3.71	5.23
						5.53	4.75
						4.64	2.63
						3.84	5.25
						5.12	3.98
						5.09	5.09
						2.53	4.54
						3.06	3.52
						4.45	3.75

						4.53	4.41
						5.21	3.71
						2.99	4.27
						4.53	3.00
						4.66	3.29
						5.23	2.90
						2.52	2.55
						2.81	3.19
						2.78	3.56
						4.66	4.83
						3.57	3.10
						4.32	2.44
						5.02	4.04
						3.24	2.43
						2.39	4.97
						3.00	4.22

Panel Y

Riverine, ♂	Riverine, ♀	Estuarine, ♂	Estuarine, ♀	Marine, ♂	Marine, ♀	Riverine, both	Estuarine, both
54.00	141.00	20.00	29.00	16.00	39.00	54.00	20.00
41.00	48.00	11.00	40.00	16.00	42.00	41.00	11.00
38.00	115.00	27.00	47.00	11.00	30.00	38.00	27.00
26.00	152.00	21.00	37.00	18.00	36.00	26.00	21.00
54.00	30.00	11.00	42.00	13.00	40.00	54.00	11.00
45.00	181.00	25.00	44.00	16.00	42.00	45.00	25.00
65.00	38.00	24.00	45.00	18.00	42.00	65.00	24.00
31.00	162.00	9.00	36.00	12.00	34.00	31.00	9.00
43.00	120.00	22.00	32.00	26.00	36.00	43.00	22.00
28.00	183.00	24.00	42.00	22.00	28.00	28.00	24.00
30.00	34.00	36.00	20.00	9.00	26.00	30.00	36.00
50.00	63.00	19.00	38.00	17.00	24.00	50.00	19.00
26.00	51.00	34.00	20.00	21.00	29.00	26.00	34.00
30.00	114.00	6.00	27.00	15.00	14.00	30.00	6.00
40.00	117.00	27.00	10.00	22.00	18.00	40.00	27.00
43.00	32.00	33.00	41.00	15.00	19.00	43.00	33.00
57.00	46.00	29.00	25.00	17.00	26.00	57.00	29.00
35.00	117.00	10.00	41.00	9.00	18.00	35.00	10.00
46.00	78.00	37.00	34.00	19.00	13.00	46.00	37.00
29.00	48.00	30.00	41.00	21.00	27.00	29.00	30.00
53.00	92.00	13.00	15.00	17.00	30.00	53.00	13.00
31.00	27.00	8.00	22.00	18.00	29.00	31.00	8.00
58.00	116.00	15.00	20.00	16.00	29.00	58.00	15.00
52.00	116.00	6.00	8.00	19.00	33.00	52.00	6.00
45.00	112.00	12.00	15.00	23.00	20.00	45.00	12.00
59.00	41.00	13.00	12.00	23.00	21.00	59.00	13.00
60.00	102.00	17.00	23.00	21.00	32.00	60.00	17.00
40.00	48.00	20.00	17.00	21.00	31.00	40.00	20.00
53.00	123.00	28.00	21.00	16.00	22.00	53.00	28.00
56.00	86.00	6.00	17.00	20.00	30.00	56.00	6.00
						141.00	29.00
						48.00	40.00
						115.00	47.00
						152.00	37.00
						30.00	42.00
						181.00	44.00
						38.00	45.00
						162.00	36.00
						120.00	32.00
						183.00	42.00
						34.00	20.00
						63.00	38.00
						51.00	20.00
						114.00	27.00
						117.00	10.00

						32.00	41.00
						46.00	25.00
						117.00	41.00
						78.00	34.00
						48.00	41.00
						92.00	15.00
						27.00	22.00
						116.00	20.00
						116.00	8.00
						112.00	15.00
						41.00	12.00
						102.00	23.00
						48.00	17.00
						123.00	21.00
						86.00	17.00

Marine, both	All, ♂	All, ♀
23.00	16.00	18.00
19.00	10.00	17.00
12.00	22.00	20.00
23.00	16.00	18.00
19.00	10.00	17.00
12.00	22.00	20.00
13.00	11.00	21.00
13.00	16.00	18.00
19.00	10.00	17.00
12.00	22.00	20.00
13.00	11.00	21.00
15.00	8.00	21.00
10.00	19.00	24.00
14.00	19.00	17.00
9.00	20.00	19.00
9.00	20.00	22.00
11.00	9.00	23.00
14.00	25.00	10.00
15.00	19.00	19.00
11.00	9.00	24.00
9.00	11.00	11.00
13.00	10.00	14.00
10.00	18.00	20.00
14.00	12.00	11.00
13.00	17.00	15.00
17.00	25.00	12.00
15.00	9.00	14.00
11.00	14.00	10.00
21.00	19.00	10.00
24.00	12.00	25.00
15.00	16.00	10.00
15.00	16.00	20.00
13.00	14.00	12.00
10.00	15.00	13.00
16.00	12.00	17.00
13.00	11.00	9.00
9.00	24.00	13.00
18.00	20.00	14.00
18.00	19.00	16.00
20.00	18.00	15.00
19.00	22.00	13.00
19.00	15.00	13.00
17.00	12.00	13.00
20.00	13.00	18.00

Panel B

Pre-spawning, ♂	Spawning, ♂	Post-spawning, ♂	Pre-spawning, ♀
25.00	16.00	14.00	10.00
19.00	10.00	19.00	19.00
9.00	22.00	12.00	24.00
11.00	11.00	16.00	11.00
10.00	8.00	16.00	14.00
18.00	19.00	14.00	20.00
12.00	19.00	15.00	11.00
17.00	20.00	12.00	15.00
25.00	20.00	11.00	12.00
9.00	9.00	24.00	14.00
19.00	20.00	13.00	11.00
12.00	19.00	14.00	8.00
10.00	18.00	8.00	16.00
11.00	22.00	20.00	23.00
11.00	15.00	21.00	18.00
8.00	12.00	8.00	8.00
24.00	13.00	23.00	10.00
21.00	13.00	12.00	19.00
19.00	11.00	14.00	15.00
13.00	20.00	13.00	14.00
14.00	13.00	11.00	15.00
15.00	19.00	21.00	19.00
11.00	12.00	24.00	12.00
9.00	13.00	15.00	9.00
13.00	15.00	15.00	18.00
10.00	10.00	13.00	11.00
14.00	14.00	10.00	19.00
13.00	9.00	16.00	17.00
17.00	9.00	13.00	13.00
15.00	11.00	9.00	10.00

23.00	13.00	15.00
17.00	11.00	15.00
15.00	20.00	14.00
15.00	19.00	11.00
19.00	12.00	8.00
12.00	10.00	16.00
9.00	11.00	23.00
18.00	11.00	18.00
11.00	8.00	8.00
19.00	24.00	10.00
17.00	21.00	19.00
13.00	19.00	15.00
10.00	13.00	14.00
23.00	13.00	13.00
15.00	14.00	18.00
18.00	8.00	8.00
22.00	20.00	19.00
9.00	21.00	12.00
18.00	8.00	13.00
15.00	23.00	23.00
20.00	12.00	17.00
15.00	14.00	10.00
20.00	13.00	20.00
	13.00	18.00
	19.00	18.00
	12.00	20.00
	13.00	19.00
	15.00	19.00
	10.00	17.00
	14.00	20.00
	9.00	23.00
	9.00	17.00
	11.00	15.00
	14.00	15.00
	15.00	19.00
	11.00	12.00
	9.00	9.00
	13.00	18.00
	10.00	11.00
	14.00	19.00
	13.00	17.00
	17.00	13.00
	15.00	10.00
	11.00	23.00
	21.00	15.00
	24.00	18.00
	15.00	22.00

	15.00	9.00
	13.00	18.00
	10.00	15.00
	16.00	20.00
	13.00	15.00
	9.00	20.00

Marine, both	All, ♂	All, ♀
26.00	17.00	20.00
21.00	10.00	19.00
13.00	24.00	21.00
14.00	12.00	22.00
16.00	9.00	22.00
22.00	21.00	26.00
14.00	20.00	18.00
9.00	21.00	19.00
9.00	22.00	23.00
21.00	9.00	26.00
15.00	26.00	11.00
27.00	20.00	20.00
11.00	10.00	25.00
9.00	11.00	12.00
14.00	10.00	15.00
21.00	20.00	27.00
25.00	12.00	22.00
13.00	19.00	15.00
18.00	26.00	13.00
17.00	10.00	16.00
21.00	15.00	11.00
22.00	21.00	10.00
27.00	13.00	27.00
17.00	18.00	11.00
15.00	27.00	21.00
24.00	26.00	13.00
16.00	27.00	23.00
17.00	14.00	24.00
14.00	12.00	9.00
9.00	27.00	14.00
22.00	25.00	15.00
21.00	22.00	16.00
24.00	21.00	10.00
22.00	25.00	14.00
21.00	17.00	14.00
19.00	13.00	14.00
24.00	16.00	19.00
27.00	16.00	16.00

Panel E

Pre-spawning, ♂	Spawning, ♂	Post-spawning, ♂	Pre-spawning, ♀
26.00	17.00	15.00	11.00
20.00	10.00	21.00	20.00
10.00	24.00	13.00	25.00
11.00	12.00	18.00	12.00
10.00	9.00	27.00	15.00
20.00	21.00	26.00	27.00
12.00	20.00	27.00	22.00
19.00	21.00	14.00	15.00
26.00	22.00	12.00	13.00
10.00	9.00	27.00	16.00
22.00	25.00	15.00	12.00
14.00	22.00	17.00	9.00
11.00	21.00	10.00	18.00
14.00	25.00	23.00	23.00
13.00	17.00	25.00	18.00
9.00	13.00	9.00	9.00
27.00	16.00	27.00	10.00
26.00	16.00	14.00	21.00
22.00	13.00	17.00	26.00
15.00	25.00	14.00	16.00
15.00	26.00	21.00	18.00
27.00	21.00	22.00	21.00
11.00	13.00	27.00	14.00
9.00	14.00	17.00	11.00
14.00	16.00	15.00	21.00
21.00	22.00	24.00	12.00
25.00	14.00	16.00	22.00
13.00	9.00	17.00	21.00
18.00	9.00	14.00	15.00
17.00	21.00	9.00	12.00

19.00	13.00	17.00
18.00	25.00	14.00
18.00	22.00	12.00
21.00	14.00	9.00
14.00	11.00	18.00
11.00	14.00	23.00
21.00	13.00	18.00
12.00	9.00	9.00
22.00	27.00	10.00
21.00	26.00	21.00
15.00	22.00	26.00
12.00	15.00	16.00
15.00	15.00	14.00
18.00	17.00	19.00
22.00	10.00	9.00
25.00	23.00	21.00
9.00	25.00	23.00
19.00	9.00	25.00
18.00	27.00	24.00
13.00	14.00	19.00
18.00	17.00	11.00
12.00	14.00	21.00
	26.00	22.00
	21.00	21.00
	13.00	24.00
	14.00	22.00
	16.00	21.00
	22.00	19.00
	14.00	24.00
	9.00	27.00
	9.00	19.00
	21.00	18.00
	15.00	18.00
	27.00	21.00
	11.00	14.00
	9.00	11.00
	14.00	21.00
	21.00	12.00
	25.00	22.00
	13.00	21.00
	18.00	15.00
	17.00	12.00
	21.00	15.00
	22.00	18.00
	27.00	22.00
	17.00	25.00
	15.00	9.00

	24.00	19.00
	16.00	18.00
	17.00	13.00
	14.00	18.00
	9.00	12.00

Marine, both	All, ♂	All, ♀
2.99	2.78	2.41
2.86	2.89	2.99
3.33	3.38	2.84
3.01	2.64	2.62
2.60	2.57	2.59
2.85	2.90	2.65
3.33	2.98	2.83
2.66	3.37	3.07
3.41	2.57	2.88
2.60	3.40	2.52
3.45	2.61	3.19
2.55	3.49	2.58
2.82	2.82	3.16
2.66	3.10	3.45
2.51	3.47	3.34
2.66	2.77	2.67
2.60	3.04	2.75
3.01	2.85	2.73
3.39	3.36	3.45
2.84	2.62	2.52
2.54	3.32	3.42
2.61	2.85	3.30
2.68	3.16	3.14
2.85	3.21	3.14
2.81	3.39	3.47
3.25	3.08	2.60
3.50	3.22	3.31
3.09	2.50	2.73
2.61	3.41	2.63
2.95	2.85	2.95
2.28	2.85	2.98
2.83	2.94	2.73
2.73	3.17	2.70
3.04	2.83	2.88
2.84	2.78	3.38
2.55	2.98	2.78
2.61	2.60	3.50
2.83	2.85	2.55
2.50	2.64	3.48

Panel H

Pre-spawning, ♂	Spawning, ♂	Post-spawning, ♂	Pre-spawning, ♀
2.61	2.78	3.32	3.19
3.49	2.89	2.85	2.58
2.82	3.38	3.16	3.16
3.10	2.64	3.21	3.45
3.47	2.57	3.39	3.34
2.77	2.90	3.08	2.67
3.04	2.98	3.22	2.75
2.85	3.37	2.50	2.73
3.36	2.57	3.41	3.45
2.62	3.40	2.85	2.52
2.87	2.85	3.34	2.83
3.36	2.94	2.53	3.05
2.97	3.17	2.94	3.47
3.31	2.83	2.94	2.90
3.00	2.78	2.89	2.61
2.81	2.98	3.27	2.63
3.20	2.60	3.06	3.40
2.93	2.85	3.15	2.77
2.81	2.64	2.95	2.85
3.10	3.12	2.63	3.39
3.45	2.99	2.54	3.02
2.55	2.86	2.61	3.04
2.82	3.33	2.68	2.52
2.66	3.01	2.85	3.16
2.51	2.60	2.81	2.79
2.66	2.85	3.25	3.44
2.60	3.33	3.50	3.22
3.01	2.66	3.09	3.34
3.39	3.41	2.61	2.51
2.84	2.60	2.95	2.92

2.96	3.12	2.91
3.02	2.87	2.83
3.04	3.36	3.05
2.52	2.97	3.47
3.16	3.31	2.90
2.79	3.00	2.61
3.44	2.81	2.63
3.22	3.20	3.40
3.34	2.93	2.77
2.51	2.81	2.85
2.92	3.10	3.39
2.97	3.34	3.12
2.71	2.53	3.28
3.31	2.94	2.89
3.25	2.94	2.79
2.63	2.89	2.82
2.61	3.27	2.71
3.31	3.06	3.27
3.20	3.15	2.92
3.26	2.95	2.51
2.88	2.63	3.06
	2.99	2.28
	2.86	2.83
	3.33	2.73
	3.01	3.04
	2.60	2.84
	2.85	2.55
	3.33	2.61
	2.66	2.83
	3.41	2.50
	2.60	2.96
	3.45	3.02
	2.55	3.04
	2.82	2.52
	2.66	3.16
	2.51	2.79
	2.66	3.44
	2.60	3.22
	3.01	3.34
	3.39	2.51
	2.84	2.92
	2.54	2.97
	2.61	2.71
	2.68	3.31
	2.85	3.25
	2.81	2.63
	3.25	2.61

	3.50	3.31
	3.09	3.20
	2.61	3.26
	2.95	2.88

Marine, both	All, ♂	All, ♀
0.82	0.77	0.82
0.77	0.77	0.81
0.81	0.80	0.76
0.81	0.83	0.76
0.81	0.76	0.83
0.80	0.78	0.76
0.83	0.85	0.85
0.82	0.78	0.79
0.80	0.82	0.85
0.81	0.77	0.84
0.80	0.78	0.80
0.77	0.79	0.79
0.82	0.80	0.78
0.84	0.78	0.77
0.78	0.83	0.85
0.81	0.79	0.81
0.81	0.79	0.81
0.84	0.82	0.83
0.78	0.80	0.82
0.82	0.82	0.85
0.82	0.81	0.85
0.79	0.84	0.82
0.80	0.81	0.82
0.85	0.77	0.82
0.83	0.85	0.77
0.82	0.81	0.78
0.85	0.83	0.78
0.83	0.82	0.78
0.80	0.82	0.85
0.77	0.78	0.84
0.84	0.79	0.85
0.85	0.78	0.83
0.85	0.81	0.83
0.84	0.78	0.81
0.84	0.84	0.85
0.84	0.81	0.82
0.84	0.80	0.81
0.84	0.80	0.82
0.85	0.79	0.85
0.81	0.84	0.84

Panel K

Pre-spawning, ♂	Spawning, ♂	Post-spawning, ♂	Pre-spawning, ♀
0.78	0.77	0.81	0.80
0.79	0.77	0.84	0.79
0.80	0.80	0.81	0.78
0.78	0.83	0.77	0.77
0.83	0.76	0.85	0.85
0.79	0.78	0.81	0.81
0.79	0.85	0.83	0.81
0.82	0.78	0.82	0.83
0.80	0.82	0.82	0.82
0.82	0.77	0.78	0.85
0.76	0.79	0.84	0.78
0.77	0.78	0.77	0.84
0.85	0.81	0.81	0.77
0.79	0.78	0.83	0.81
0.81	0.84	0.76	0.78
0.81	0.81	0.79	0.80
0.79	0.80	0.82	0.79
0.80	0.80	0.77	0.76
0.78	0.79	0.82	0.82
0.85	0.84	0.77	0.76
0.80	0.82	0.82	0.82
0.77	0.77	0.79	0.85
0.82	0.81	0.80	0.81
0.84	0.81	0.85	0.84
0.78	0.81	0.83	0.79
0.81	0.80	0.82	0.77
0.81	0.83	0.85	0.76
0.84	0.82	0.83	0.77
0.78	0.80	0.80	0.78
0.82	0.81	0.77	0.78

0.82	0.76	0.78
0.85	0.77	0.84
0.81	0.85	0.77
0.84	0.79	0.81
0.79	0.81	0.78
0.77	0.81	0.80
0.76	0.79	0.79
0.77	0.80	0.76
0.78	0.78	0.82
0.78	0.85	0.76
0.77	0.84	0.79
0.76	0.77	0.81
0.77	0.81	0.84
0.76	0.83	0.78
0.77	0.76	0.83
0.78	0.79	0.83
0.83	0.82	0.79
0.80	0.77	0.80
0.77	0.82	0.83
0.76	0.77	0.83
	0.82	0.84
	0.77	0.85
	0.81	0.85
	0.81	0.84
	0.81	0.84
	0.80	0.84
	0.83	0.84
	0.82	0.84
	0.80	0.85
	0.81	0.81
	0.80	0.82
	0.77	0.85
	0.82	0.81
	0.84	0.84
	0.78	0.79
	0.81	0.77
	0.81	0.76
	0.84	0.77
	0.78	0.78
	0.82	0.78
	0.82	0.77
	0.79	0.76
	0.80	0.77
	0.85	0.76
	0.83	0.77
	0.82	0.78
	0.85	0.83

	0.83	0.80
	0.80	0.77
	0.77	0.76

Marine, both	All, ♂	All, ♀
0.99	1.24	1.20
1.25	0.87	1.17
1.23	0.94	1.28
0.96	1.09	1.12
0.99	1.23	1.11
1.29	0.87	1.12
1.25	1.00	1.19
0.96	1.02	1.19
0.98	1.32	1.15
1.31	0.86	1.19
1.22	0.92	0.97
0.90	1.03	0.85
1.00	1.29	1.18
1.05	0.87	0.94
1.19	0.98	1.03
0.95	1.04	0.88
0.98	1.28	1.23
1.36	0.92	1.01
1.18	0.95	1.00
0.97	1.08	0.92
1.00	1.30	1.29
1.28	1.38	1.00
1.21	1.25	1.00
1.00	1.00	0.88
1.01	1.37	1.29
1.32	0.90	1.02
1.03	1.34	1.27
1.35	0.91	1.00
1.21	0.97	1.03
0.91	1.03	0.90
1.09	1.12	1.11
1.14	1.03	1.17
1.36	1.07	1.18
1.13	1.11	1.14
1.19	1.19	1.12
1.15	1.07	1.16
1.24	1.05	1.15
1.19	1.05	1.11
1.19	1.12	1.12
1.17	1.11	1.12
1.33	1.00	1.10

Panel N

Pre-spawning, ♂	Spawning, ♂	Post-spawning, ♂	Pre-spawning, ♀
0.92	1.24	1.30	0.97
1.03	0.87	1.38	0.85
1.29	0.94	1.25	1.18
0.87	1.09	1.00	0.94
0.98	1.23	1.37	1.03
1.04	0.87	0.90	0.88
1.28	1.00	1.34	1.23
0.92	1.02	0.91	1.01
0.95	1.32	0.97	1.00
1.08	0.86	1.03	0.92
1.00	1.12	1.19	1.10
1.40	1.03	1.02	0.98
1.20	1.07	1.07	1.02
1.04	1.11	1.01	1.15
1.06	1.19	1.08	1.20
1.07	1.07	1.04	0.94
1.11	1.05	1.16	1.09
1.06	1.05	1.02	1.08
1.03	1.12	1.08	1.16
1.03	1.11	1.39	0.99
1.22	0.99	1.00	1.33
0.90	1.25	1.28	1.00
1.00	1.23	1.21	0.90
1.05	0.96	1.00	1.11
1.19	0.99	1.01	1.37
0.95	1.29	1.32	0.91
0.98	1.25	1.03	1.29
1.36	0.96	1.35	1.08
1.18	0.98	1.21	1.31
0.97	1.31	0.91	0.92

1.00	1.40	0.98
0.90	1.20	1.02
1.11	1.04	1.15
1.37	1.06	1.20
0.91	1.07	0.94
1.29	1.11	1.09
1.08	1.06	1.08
1.31	1.03	1.16
0.92	1.03	0.99
0.91	1.19	1.04
1.00	1.02	1.15
1.37	1.07	1.18
1.20	1.01	0.91
1.19	1.08	1.10
1.03	1.04	1.18
1.13	1.16	1.04
1.06	1.02	1.13
1.25	1.08	1.18
1.29	1.39	0.97
	0.99	1.09
	1.25	1.14
	1.23	1.36
	0.96	1.13
	0.99	1.19
	1.29	1.15
	1.25	1.24
	0.96	1.19
	0.98	1.19
	1.31	1.17
	1.22	1.33
	0.90	1.00
	1.00	0.90
	1.05	1.11
	1.19	1.37
	0.95	0.91
	0.98	1.29
	1.36	1.08
	1.18	1.31
	0.97	0.92
	1.00	0.91
	1.28	1.00
	1.21	1.37
	1.00	1.20
	1.01	1.19
	1.32	1.03
	1.03	1.13
	1.35	1.06

	1.21	1.25
	0.91	1.29

Marine, both	All, ♂	All, ♀
190.00	178.00	193.00
194.00	151.00	192.00
191.00	182.00	206.00
191.00	136.00	208.00
186.00	169.00	186.00
199.00	141.00	205.00
187.00	152.00	194.00
194.00	177.00	196.00
185.00	165.00	186.00
185.00	147.00	212.00
168.00	150.00	138.00
148.00	197.00	158.00
178.00	160.00	179.00
172.00	193.00	193.00
172.00	181.00	160.00
151.00	180.00	187.00
178.00	191.00	196.00
160.00	186.00	168.00
192.00	188.00	167.00
150.00	182.00	180.00
141.00	171.00	172.00
141.00	183.00	178.00
140.00	161.00	169.00
135.00	180.00	167.00
142.00	166.00	158.00
126.00	163.00	167.00
131.00	169.00	157.00
128.00	161.00	166.00
130.00	160.00	156.00
155.00	148.00	154.00
201.00	141.00	158.00
192.00	163.00	159.00
217.00	163.00	170.00
192.00	145.00	145.00
214.00	133.00	131.00
212.00	135.00	146.00
195.00	166.00	139.00
203.00	160.00	151.00
208.00	157.00	160.00
190.00	132.00	146.00
159.00	156.00	126.00
166.00	143.00	154.00

Panel Q

Pre-spawning, ♂	Spawning, ♂	Post-spawning, ♂	Pre-spawning, ♀
150.00	178.00	171.00	138.00
197.00	151.00	183.00	158.00
160.00	182.00	161.00	179.00
193.00	136.00	180.00	193.00
181.00	169.00	166.00	160.00
180.00	141.00	163.00	187.00
191.00	152.00	169.00	196.00
186.00	177.00	161.00	168.00
188.00	165.00	160.00	167.00
182.00	147.00	148.00	180.00
156.00	141.00	171.00	126.00
143.00	163.00	187.00	154.00
161.00	163.00	163.00	166.00
157.00	145.00	168.00	189.00
157.00	133.00	170.00	182.00
187.00	135.00	161.00	183.00
196.00	166.00	166.00	170.00
174.00	160.00	170.00	159.00
185.00	157.00	158.00	185.00
183.00	132.00	170.00	184.00
168.00	190.00	141.00	159.00
148.00	194.00	141.00	166.00
178.00	191.00	140.00	192.00
172.00	191.00	135.00	150.00
172.00	186.00	142.00	148.00
151.00	199.00	126.00	159.00
178.00	187.00	131.00	157.00
160.00	194.00	128.00	162.00
192.00	185.00	130.00	174.00
150.00	185.00	155.00	129.00

192.00	161.00	166.00
150.00	157.00	189.00
148.00	157.00	182.00
159.00	187.00	183.00
157.00	196.00	170.00
162.00	174.00	159.00
174.00	185.00	185.00
129.00	183.00	184.00
129.00	171.00	160.00
148.00	187.00	187.00
140.00	163.00	165.00
143.00	168.00	167.00
127.00	170.00	166.00
130.00	161.00	168.00
144.00	166.00	165.00
131.00	170.00	157.00
144.00	158.00	165.00
127.00	170.00	149.00
	190.00	201.00
	194.00	192.00
	191.00	217.00
	191.00	192.00
	186.00	214.00
	199.00	212.00
	187.00	195.00
	194.00	203.00
	185.00	208.00
	185.00	190.00
	168.00	159.00
	148.00	166.00
	178.00	192.00
	172.00	150.00
	172.00	148.00
	151.00	159.00
	178.00	157.00
	160.00	162.00
	192.00	174.00
	150.00	129.00
	141.00	129.00
	141.00	148.00
	140.00	140.00
	135.00	143.00
	142.00	127.00
	126.00	130.00
	131.00	144.00
	128.00	131.00
	130.00	144.00

	155.00	127.00

Marine, both	All, ♂	All, ♀
0.12	0.15	0.12
0.14	0.10	0.11
0.14	0.08	0.14
0.11	0.14	0.12
0.14	0.12	0.12
0.15	0.13	0.14
0.12	0.09	0.13
0.15	0.12	0.14
0.14	0.09	0.13
0.13	0.08	0.14
0.13	0.14	0.08
0.09	0.09	0.13
0.15	0.11	0.14
0.12	0.07	0.10
0.15	0.12	0.09
0.15	0.08	0.08
0.08	0.14	0.12
0.09	0.12	0.11
0.08	0.09	0.11
0.13	0.12	0.09
0.12	0.15	0.12
0.10	0.12	0.14
0.14	0.14	0.12
0.08	0.15	0.12
0.14	0.12	0.14
0.13	0.11	0.10
0.09	0.15	0.09
0.09	0.13	0.15
0.11	0.14	0.09
0.08	0.14	0.12
0.14	0.14	0.14
0.14	0.15	0.14
0.13	0.15	0.13
0.14	0.15	0.14
0.14	0.13	0.14
0.13	0.15	0.13
0.13	0.12	0.14
0.14	0.14	0.14
0.13	0.14	0.14
0.15	0.14	0.15
0.14	0.12	0.12
0.08	0.14	0.08
0.09	0.13	0.14

Panel T

Pre-spawning, ♂	Spawning, ♂	Post-spawning, ♂	Pre-spawning, ♀
0.14	0.15	0.15	0.08
0.09	0.10	0.12	0.13
0.11	0.08	0.14	0.14
0.07	0.14	0.15	0.10
0.12	0.12	0.12	0.09
0.08	0.13	0.11	0.08
0.14	0.09	0.15	0.12
0.12	0.12	0.13	0.11
0.09	0.09	0.14	0.11
0.12	0.08	0.14	0.09
0.12	0.14	0.15	0.12
0.14	0.15	0.13	0.08
0.13	0.15	0.13	0.14
0.14	0.15	0.15	0.11
0.14	0.13	0.12	0.15
0.12	0.15	0.12	0.14
0.13	0.12	0.08	0.14
0.14	0.14	0.14	0.14
0.15	0.14	0.15	0.14
0.13	0.14	0.13	0.14
0.13	0.12	0.12	0.14
0.09	0.14	0.10	0.08
0.15	0.14	0.14	0.09
0.12	0.11	0.08	0.12
0.15	0.14	0.14	0.07
0.15	0.15	0.13	0.08
0.08	0.12	0.09	0.08
0.09	0.15	0.09	0.11
0.08	0.14	0.11	0.12
0.13	0.13	0.08	0.14

0.12	0.14	0.11
0.07	0.14	0.15
0.08	0.12	0.14
0.08	0.13	0.14
0.11	0.14	0.14
0.12	0.15	0.14
0.14	0.13	0.14
0.08	0.15	0.12
0.09	0.13	0.09
0.10	0.13	0.12
0.07	0.15	0.14
0.10	0.12	0.15
0.11	0.12	0.10
0.11	0.08	0.08
0.13	0.14	0.10
0.14	0.15	0.09
0.14	0.13	0.07
	0.12	0.14
	0.14	0.14
	0.14	0.13
	0.11	0.14
	0.14	0.14
	0.15	0.13
	0.12	0.13
	0.15	0.14
	0.14	0.13
	0.13	0.15
	0.13	0.14
	0.09	0.08
	0.15	0.09
	0.12	0.12
	0.15	0.07
	0.15	0.08
	0.08	0.08
	0.09	0.11
	0.08	0.12
	0.13	0.14
	0.12	0.08
	0.10	0.09
	0.14	0.10
	0.08	0.07
	0.14	0.10
	0.13	0.11
	0.09	0.11
	0.09	0.13
	0.11	0.14
	0.08	0.14

Marine, both	All, ♂	All, ♀
4.47	2.82	3.78
4.77	3.50	4.91
3.51	5.09	3.78
4.94	5.03	5.70
4.57	4.85	5.43
4.82	4.27	3.71
3.47	4.75	5.53
4.61	3.48	4.64
4.17	2.69	3.84
2.96	5.02	5.12
4.57	3.66	5.09
5.12	4.29	2.53
2.76	3.64	3.06
5.02	3.16	4.45
2.68	4.16	4.53
3.82	3.38	5.21
3.94	4.97	2.99
4.67	3.62	4.53
3.27	3.09	4.66
3.14	3.29	5.23
2.81	3.50	2.52
4.59	2.86	2.81
5.29	4.45	2.78
5.25	2.38	4.66
2.94	2.75	3.57
2.93	3.39	4.32
4.35	2.69	5.02
4.79	3.61	3.24
3.01	4.35	2.39
3.99	2.67	3.00
4.00	4.67	4.96
3.89	4.52	3.34
4.41	3.85	5.50
5.50	2.94	2.53
3.75	3.30	5.04
3.87	3.94	5.23
5.15	4.68	4.75
5.34	5.23	2.63
5.76	3.71	5.25
5.18	3.31	3.98
2.55	2.35	5.09
3.60	3.17	4.54
3.39	4.52	3.52
5.20	3.91	3.75

Panel W

Pre-spawning, ♂	Spawning, ♂	Post-spawning, ♂	Pre-spawning, ♀
3.66	2.82	3.50	5.09
4.29	3.50	2.86	2.53
3.64	5.09	4.45	3.06
3.16	5.03	2.38	4.45
4.16	4.85	2.75	4.53
3.38	4.27	3.39	5.21
4.97	4.75	2.69	2.99
3.62	3.48	3.61	4.53
3.09	2.69	4.35	4.66
3.29	5.02	2.67	5.23
2.35	4.67	2.67	5.09
3.17	4.52	4.70	4.54
4.52	3.85	3.87	3.52
3.91	2.94	5.22	3.75
4.74	3.30	4.76	4.41
4.93	3.94	2.79	3.71
2.47	4.68	2.48	4.27
4.91	5.23	3.87	3.00
3.48	3.71	4.51	3.29
3.72	3.31	2.35	2.90
4.57	4.47	2.81	2.55
5.12	4.77	4.59	3.60
2.76	3.51	5.29	3.39
5.02	4.94	5.25	5.20
2.68	4.57	2.94	2.82
3.82	4.82	2.93	4.21
3.94	3.47	4.35	3.33
4.67	4.61	4.79	4.27
3.27	4.17	3.01	3.24
3.14	2.96	3.99	2.69

2.82	4.74	4.41
4.21	4.93	3.71
3.33	2.47	4.27
4.27	4.91	3.00
3.24	3.48	3.29
2.69	3.72	2.90
5.26	2.67	2.55
4.71	4.70	3.19
4.39	3.87	3.56
4.52	5.22	4.83
4.96	4.76	3.10
4.45	2.79	2.44
4.87	2.48	4.04
3.95	3.87	2.43
4.71	4.51	4.97
3.28	2.35	4.22
	4.47	4.00
	4.77	3.89
	3.51	4.41
	4.94	5.50
	4.57	3.75
	4.82	3.87
	3.47	5.15
	4.61	5.34
	4.17	5.76
	2.96	5.18
	4.57	2.55
	5.12	3.60
	2.76	3.39
	5.02	5.20
	2.68	2.82
	3.82	4.21
	3.94	3.33
	4.67	4.27
	3.27	3.24
	3.14	2.69
	2.81	5.26
	4.59	4.71
	5.29	4.39
	5.25	4.52
	2.94	4.96
	2.93	4.45
	4.35	4.87
	4.79	3.95
	3.01	4.71
	3.99	3.28

Marine, both	All, ♂	All, ♀
16.00	54.00	141.00
16.00	41.00	48.00
11.00	38.00	115.00
18.00	26.00	152.00
13.00	54.00	30.00
16.00	45.00	181.00
18.00	65.00	38.00
12.00	31.00	162.00
26.00	43.00	120.00
22.00	28.00	183.00
9.00	30.00	34.00
17.00	50.00	63.00
21.00	26.00	51.00
15.00	30.00	114.00
22.00	40.00	117.00
15.00	43.00	32.00
17.00	57.00	46.00
9.00	35.00	117.00
19.00	46.00	78.00
21.00	29.00	48.00
17.00	53.00	92.00
18.00	31.00	27.00
16.00	58.00	116.00
19.00	52.00	116.00
23.00	45.00	112.00
23.00	59.00	41.00
21.00	60.00	102.00
21.00	40.00	48.00
16.00	53.00	123.00
20.00	56.00	86.00
39.00	20.00	29.00
42.00	11.00	40.00
30.00	27.00	47.00
36.00	21.00	37.00
40.00	11.00	42.00
42.00	25.00	44.00
42.00	24.00	45.00
34.00	9.00	36.00
36.00	22.00	32.00
28.00	24.00	42.00
26.00	36.00	20.00
24.00	19.00	38.00
29.00	34.00	20.00
14.00	6.00	27.00
18.00	27.00	10.00

Panel Z

Pre-spawning, ♂	Spawning, ♂	Post-spawning, ♂	Pre-spawning, ♀
30.00	54.00	53.00	34.00
50.00	41.00	31.00	63.00
26.00	38.00	58.00	51.00
30.00	26.00	52.00	114.00
40.00	54.00	45.00	117.00
43.00	45.00	59.00	32.00
57.00	65.00	60.00	46.00
35.00	31.00	40.00	117.00
46.00	43.00	53.00	78.00
29.00	28.00	56.00	48.00
36.00	20.00	13.00	20.00
19.00	11.00	8.00	38.00
34.00	27.00	15.00	20.00
6.00	21.00	6.00	27.00
27.00	11.00	12.00	10.00
33.00	25.00	13.00	41.00
29.00	24.00	17.00	25.00
10.00	9.00	20.00	41.00
37.00	22.00	28.00	34.00
30.00	24.00	6.00	41.00
9.00	16.00	17.00	26.00
17.00	16.00	18.00	24.00
21.00	11.00	16.00	29.00
15.00	18.00	19.00	14.00
22.00	13.00	23.00	18.00
15.00	16.00	23.00	19.00
17.00	18.00	21.00	26.00
9.00	12.00	21.00	18.00
19.00	26.00	16.00	13.00
21.00	22.00	20.00	27.00

19.00	33.00	41.00
26.00	29.00	25.00
18.00	10.00	41.00
13.00	37.00	34.00
27.00	30.00	41.00
30.00	13.00	15.00
29.00	8.00	22.00
29.00	15.00	20.00
33.00	6.00	8.00
20.00	12.00	15.00
21.00	13.00	12.00
32.00	17.00	23.00
31.00	20.00	17.00
22.00	28.00	21.00
30.00	6.00	17.00
	16.00	39.00
	16.00	42.00
	11.00	30.00
	18.00	36.00
	13.00	40.00
	16.00	42.00
	18.00	42.00
	12.00	34.00
	26.00	36.00
	22.00	28.00
	9.00	26.00
	17.00	24.00
	21.00	29.00
	15.00	14.00
	22.00	18.00
	15.00	19.00
	17.00	26.00
	9.00	18.00
	19.00	13.00
	21.00	27.00
	17.00	30.00
	18.00	29.00
	16.00	29.00
	19.00	33.00
	23.00	20.00
	23.00	21.00
	21.00	32.00
	21.00	31.00
	16.00	22.00
	20.00	30.00

Spawning, ♀	Post-spawning, ♀
18.00	10.00
17.00	10.00
20.00	25.00
21.00	10.00
21.00	20.00
24.00	12.00
17.00	13.00
19.00	17.00
22.00	9.00
23.00	13.00
14.00	13.00
16.00	18.00
15.00	8.00
13.00	19.00
13.00	12.00
13.00	13.00
18.00	23.00
15.00	17.00
15.00	10.00
14.00	20.00
18.00	23.00
18.00	15.00
20.00	18.00
19.00	22.00
19.00	9.00
17.00	18.00
20.00	15.00
23.00	20.00
17.00	15.00
15.00	20.00

Panel C

>23–40, ♂	>23–40, ♀	41–60, ♂	41–60, ♀	60+, ♂	60+, ♀
16.00	18.00	12.00	8.00	9.00	17.00
10.00	17.00	10.00	16.00	13.00	13.00
22.00	20.00	11.00	23.00	10.00	10.00
11.00	21.00	11.00	18.00	14.00	23.00
8.00	21.00	8.00	8.00	21.00	15.00
19.00	24.00	14.00	18.00	24.00	18.00
19.00	17.00	8.00	8.00	15.00	22.00
20.00	19.00	20.00	19.00	15.00	9.00
20.00	22.00	21.00	12.00	13.00	18.00
9.00	23.00	8.00	13.00	10.00	15.00
25.00	10.00	23.00	23.00	16.00	20.00
19.00	19.00	12.00	17.00	13.00	15.00
9.00	24.00	14.00	10.00	9.00	20.00
11.00	11.00	13.00	20.00		10.00
10.00	14.00	13.00	18.00		23.00
18.00	20.00	19.00	18.00		
12.00	11.00	12.00	20.00		
17.00	15.00	13.00	19.00		
25.00	12.00	15.00	19.00		
9.00	14.00	10.00	17.00		
14.00	10.00	14.00	20.00		
19.00	10.00	9.00	23.00		
12.00	25.00	9.00	17.00		
16.00	10.00	11.00	15.00		
16.00	20.00	14.00	15.00		
14.00	12.00	15.00	19.00		
15.00	13.00	11.00			
12.00	17.00	13.00			
11.00	9.00	17.00			
24.00	13.00	15.00			
20.00	14.00	11.00			
19.00	16.00				
18.00	15.00				
22.00	13.00				
15.00	13.00				
12.00	13.00				
13.00	18.00				
13.00	15.00				
11.00	15.00				
20.00	14.00				
19.00	11.00				
24.00	10.00				
21.00	19.00				
19.00	15.00				

13.00	14.00				
13.00	13.00				
	12.00				
	17.00				
	13.00				

Spawning, ♀	Post-spawning, ♀
20.00	11.00
19.00	10.00
21.00	27.00
22.00	11.00
22.00	21.00
26.00	13.00
18.00	23.00
19.00	24.00
23.00	9.00
26.00	14.00
15.00	14.00
16.00	19.00
10.00	9.00
14.00	21.00
14.00	23.00
14.00	25.00
19.00	24.00
16.00	19.00
17.00	11.00
14.00	21.00
22.00	15.00
21.00	18.00
24.00	22.00
22.00	25.00
21.00	9.00
19.00	19.00
24.00	18.00
27.00	13.00
19.00	18.00
18.00	12.00

Panel F

>23–40, ♂	>23–40, ♀	41–60, ♂	41–60, ♀	60+, ♂	60+, ♀
17.00	20.00	14.00	9.00	9.00	21.00
10.00	19.00	11.00	18.00	14.00	15.00
24.00	21.00	14.00	23.00	21.00	12.00
12.00	22.00	13.00	18.00	25.00	15.00
9.00	22.00	9.00	9.00	22.00	18.00
21.00	26.00	17.00	19.00	27.00	22.00
20.00	18.00	10.00	9.00	17.00	25.00
21.00	19.00	23.00	21.00	15.00	9.00
22.00	23.00	25.00	23.00	24.00	19.00
9.00	26.00	9.00	25.00	16.00	18.00
26.00	11.00	27.00	24.00	17.00	13.00
20.00	20.00	14.00	19.00	14.00	18.00
10.00	25.00	17.00	11.00	9.00	12.00
11.00	12.00	14.00	21.00		12.00
10.00	15.00	26.00	22.00		15.00
20.00	27.00	21.00	21.00		
12.00	22.00	13.00	24.00		
19.00	15.00	14.00	22.00		
26.00	13.00	16.00	21.00		
10.00	16.00	22.00	19.00		
15.00	11.00	14.00	24.00		
21.00	10.00	9.00	27.00		
13.00	27.00	9.00	19.00		
18.00	11.00	21.00	18.00		
27.00	21.00	15.00	18.00		
26.00	13.00	27.00	21.00		
27.00	23.00	11.00			
14.00	24.00	13.00			
12.00	9.00	18.00			
27.00	14.00	17.00			
25.00	15.00	21.00			
22.00	16.00				
21.00	10.00				
25.00	14.00				
17.00	14.00				
13.00	14.00				
16.00	19.00				
16.00	16.00				

13.00	17.00				
25.00	14.00				
22.00	12.00				
27.00	10.00				
26.00	21.00				
22.00	26.00				
15.00	16.00				
15.00	14.00				
	14.00				
	21.00				
	15.00				

Spawning, ♀	Post-spawning, ♀
2.41	3.42
2.99	3.30
2.84	3.14
2.62	3.14
2.59	3.47
2.65	2.60
2.83	3.31
3.07	2.73
2.88	2.63
2.52	2.95
2.98	3.12
2.73	3.28
2.70	2.89
2.88	2.79
3.38	2.82
2.78	2.71
3.50	3.27
2.55	2.92
3.48	2.51
2.91	3.06
2.28	2.97
2.83	2.71
2.73	3.31
3.04	3.25
2.84	2.63
2.55	2.61
2.61	3.31
2.83	3.20
2.50	3.26
2.96	2.88

Panel I

>23–40, ♂	>23–40, ♀	41–60, ♂	41–60, ♀	60+, ♂	60+, ♀
2.78	2.41	3.36	3.05	2.66	3.34
2.89	2.99	2.97	3.47	2.51	2.51
3.38	2.84	3.31	2.90	2.66	2.92
2.64	2.62	3.00	2.61	2.60	2.97
2.57	2.59	2.81	2.63	2.61	2.71
2.90	2.65	2.53	3.28	2.68	3.31
2.98	2.83	2.94	2.89	2.85	3.25
3.37	3.07	2.94	2.79	2.81	2.63
2.57	2.88	2.89	2.82	3.25	2.61
3.40	2.52	3.27	2.71	3.50	3.31
2.61	3.19	3.06	3.27	3.09	3.20
3.49	2.58	3.15	2.92	2.61	3.26
2.82	3.16	2.95	2.51	2.95	2.88
3.10	3.45	2.63	3.06		2.92
3.47	3.34	2.99	2.28		2.97
2.77	2.67	2.86	2.83		
3.04	2.75	3.33	2.73		
2.85	2.73	3.01	3.04		
3.36	3.45	2.60	2.84		
2.62	2.52	2.85	2.55		
3.32	3.42	3.33	2.61		
2.85	3.30	2.66	2.83		
3.16	3.14	3.41	2.50		
3.21	3.14	2.60	2.96		
3.39	3.47	3.45	3.02		
3.08	2.60	2.55	3.04		
3.22	3.31	2.82			
2.50	2.73	3.01			
3.41	2.63	3.39			
2.85	2.95	2.84			
2.85	2.98	2.54			
2.94	2.73				
3.17	2.70				
2.83	2.88				
2.78	3.38				
2.98	2.78				
2.60	3.50				
2.85	2.55				
2.64	3.48				

3.12	2.91				
2.87	2.83				
3.20	3.40				
2.93	2.77				
2.81	2.85				
3.10	3.39				
3.34	3.12				
	2.52				
	3.34				
	2.51				

Spawning, ♀	Post-spawning, ♀
0.82	0.85
0.81	0.82
0.76	0.82
0.76	0.82
0.83	0.77
0.76	0.78
0.85	0.78
0.79	0.78
0.85	0.85
0.84	0.84
0.85	0.79
0.83	0.81
0.83	0.84
0.81	0.78
0.85	0.83
0.82	0.83
0.81	0.79
0.82	0.80
0.85	0.83
0.84	0.83
0.84	0.77
0.85	0.76
0.85	0.77
0.84	0.76
0.84	0.77
0.84	0.78
0.84	0.83
0.84	0.80
0.85	0.77
0.81	0.76

Panel L

>23–40, ♂	>23–40, ♀	41–60, ♂	41–60, ♀	60+, ♂	60+, ♀
0.77	0.82	0.77	0.84	0.84	0.77
0.77	0.81	0.85	0.77	0.78	0.78
0.80	0.76	0.79	0.81	0.81	0.78
0.83	0.76	0.81	0.78	0.81	0.77
0.76	0.83	0.81	0.80	0.79	0.76
0.78	0.76	0.77	0.81	0.80	0.77
0.85	0.85	0.81	0.84	0.85	0.76
0.78	0.79	0.83	0.78	0.83	0.77
0.82	0.85	0.76	0.83	0.82	0.78
0.77	0.84	0.79	0.83	0.85	0.83
0.78	0.80	0.82	0.79	0.83	0.80
0.79	0.79	0.77	0.80	0.80	0.77
0.80	0.78	0.82	0.83	0.77	0.76
0.78	0.77	0.77	0.83		0.78
0.83	0.85	0.82	0.84		0.77
0.79	0.81	0.77	0.85		
0.79	0.81	0.81	0.85		
0.82	0.83	0.81	0.84		
0.80	0.82	0.81	0.84		
0.82	0.85	0.80	0.84		
0.81	0.85	0.83	0.84		
0.84	0.82	0.82	0.84		
0.81	0.82	0.80	0.85		
0.77	0.82	0.81	0.81		
0.85	0.77	0.80	0.82		
0.81	0.78	0.77	0.85		
0.83	0.78	0.82			
0.82	0.78	0.84			
0.82	0.85	0.78			
0.78	0.84	0.82			
0.79	0.85	0.82			
0.78	0.83				
0.81	0.83				
0.78	0.81				
0.84	0.85				
0.81	0.82				
0.80	0.81				
0.80	0.82				
0.79	0.85				
0.84	0.84				

0.76	0.78				
0.79	0.79				
0.80	0.76				
0.78	0.82				
0.85	0.76				
0.84	0.79				
	0.81				
	0.77				
	0.78				

Spawning, ♀	Post-spawning, ♀
1.20	1.29
1.17	1.00
1.28	1.00
1.12	0.88
1.11	1.29
1.12	1.02
1.19	1.27
1.19	1.00
1.15	1.03
1.19	0.90
1.11	1.04
1.17	1.15
1.18	1.18
1.14	0.91
1.12	1.10
1.16	1.18
1.15	1.04
1.11	1.13
1.12	1.18
1.12	0.97
1.09	0.91
1.14	1.00
1.36	1.37
1.13	1.20
1.19	1.19
1.15	1.03
1.24	1.13
1.19	1.06
1.19	1.25
1.17	1.29

Panel O

>23–40, ♂	>23–40, ♀	41–60, ♂	41–60, ♀	60+, ♂	60+, ♀
1.24	1.20	1.40	0.98	1.05	1.08
0.87	1.17	1.20	1.02	1.19	1.31
0.94	1.28	1.04	1.15	0.95	0.92
1.09	1.12	1.06	1.20	0.98	0.91
1.23	1.11	1.07	0.94	1.28	1.00
0.87	1.12	1.02	1.15	1.21	1.37
1.00	1.19	1.07	1.18	1.00	1.20
1.02	1.19	1.01	0.91	1.01	1.19
1.32	1.15	1.08	1.10	1.32	1.03
0.86	1.19	1.04	1.18	1.03	1.13
0.92	0.97	1.16	1.04	1.35	1.06
1.03	0.85	1.02	1.13	1.21	1.25
1.29	1.18	1.08	1.18	0.91	1.29
0.87	0.94	1.39	0.97		0.92
0.98	1.03	0.99	1.09		0.91
1.04	0.88	1.25	1.14		
1.28	1.23	1.23	1.36		
0.92	1.01	0.96	1.13		
0.95	1.00	0.99	1.19		
1.08	0.92	1.29	1.15		
1.30	1.29	1.25	1.24		
1.38	1.00	0.96	1.19		
1.25	1.00	0.98	1.19		
1.00	0.88	1.31	1.17		
1.37	1.29	1.22	1.33		
0.90	1.02	0.90	1.00		
1.34	1.27	1.00			
0.91	1.00	1.36			
0.97	1.03	1.18			
1.03	0.90	0.97			
1.12	1.11	1.00			
1.03	1.17				
1.07	1.18				
1.11	1.14				
1.19	1.12				
1.07	1.16				
1.05	1.15				
1.05	1.11				
1.12	1.12				
1.11	1.12				
1.00	1.10				

1.11	1.09				
1.06	1.08				
1.03	1.16				
1.03	0.99				
1.19	1.04				
	0.90				
	1.08				
	1.31				

Spawning, ♀	Post-spawning, ♀
193.00	172.00
192.00	178.00
206.00	169.00
208.00	167.00
186.00	158.00
205.00	167.00
194.00	157.00
196.00	166.00
186.00	156.00
212.00	154.00
158.00	160.00
159.00	187.00
170.00	165.00
145.00	167.00
131.00	166.00
146.00	168.00
139.00	165.00
151.00	157.00
160.00	165.00
146.00	149.00
201.00	129.00
192.00	148.00
217.00	140.00
192.00	143.00
214.00	127.00
212.00	130.00
195.00	144.00
203.00	131.00
208.00	144.00
190.00	127.00

Panel R

>23–40, ♂	>23–40, ♀	41–60, ♂	41–60, ♀	60+, ♂	60+, ♀
178.00	193.00	143.00	154.00	172.00	162.00
151.00	192.00	161.00	166.00	172.00	174.00
182.00	206.00	157.00	189.00	151.00	129.00
136.00	208.00	157.00	182.00	178.00	129.00
169.00	186.00	187.00	183.00	141.00	148.00
141.00	205.00	187.00	187.00	140.00	140.00
152.00	194.00	163.00	165.00	135.00	143.00
177.00	196.00	168.00	167.00	142.00	127.00
165.00	186.00	170.00	166.00	126.00	130.00
147.00	212.00	161.00	168.00	131.00	144.00
150.00	138.00	166.00	165.00	128.00	131.00
197.00	158.00	170.00	157.00	130.00	144.00
160.00	179.00	158.00	165.00	155.00	127.00
193.00	193.00	170.00	149.00		129.00
181.00	160.00	190.00	201.00		129.00
180.00	187.00	194.00	192.00		
191.00	196.00	191.00	217.00		
186.00	168.00	191.00	192.00		
188.00	167.00	186.00	214.00		
182.00	180.00	199.00	212.00		
171.00	172.00	187.00	195.00		
183.00	178.00	194.00	203.00		
161.00	169.00	185.00	208.00		
180.00	167.00	185.00	190.00		
166.00	158.00	168.00	159.00		
163.00	167.00	148.00	166.00		
169.00	157.00	178.00			
161.00	166.00	160.00			
160.00	156.00	192.00			
148.00	154.00	150.00			
141.00	158.00	141.00			
163.00	159.00				
163.00	170.00				
145.00	145.00				
133.00	131.00				
135.00	146.00				
166.00	139.00				
160.00	151.00				
157.00	160.00				
132.00	146.00				
156.00	126.00				
196.00	170.00				

174.00	159.00				
185.00	185.00				
183.00	184.00				
171.00	160.00				
	192.00				
	162.00				
	174.00				

Spawning, ♀	Post-spawning, ♀
0.12	0.12
0.11	0.14
0.14	0.12
0.12	0.12
0.12	0.14
0.14	0.10
0.13	0.09
0.14	0.15
0.13	0.09
0.14	0.12
0.14	0.12
0.14	0.09
0.13	0.12
0.14	0.14
0.14	0.15
0.13	0.10
0.14	0.08
0.14	0.10
0.14	0.09
0.15	0.07
0.14	0.08
0.14	0.09
0.13	0.10
0.14	0.07
0.14	0.10
0.13	0.11
0.13	0.11
0.14	0.13
0.13	0.14
0.15	0.14

Panel U

>23–40, ♂	>23–40, ♀	41–60, ♂	41–60, ♀	60+, ♂	60+, ♀
0.15	0.12	0.14	0.08	0.12	0.11
0.10	0.11	0.13	0.14	0.15	0.12
0.08	0.14	0.14	0.11	0.15	0.14
0.14	0.12	0.14	0.15	0.08	0.08
0.12	0.12	0.12	0.14	0.10	0.09
0.13	0.14	0.13	0.09	0.14	0.10
0.09	0.13	0.13	0.12	0.08	0.07
0.12	0.14	0.15	0.14	0.14	0.10
0.09	0.13	0.12	0.15	0.13	0.11
0.08	0.14	0.12	0.10	0.09	0.11
0.14	0.08	0.08	0.08	0.09	0.13
0.09	0.13	0.14	0.10	0.11	0.14
0.11	0.14	0.15	0.09	0.08	0.14
0.07	0.10	0.13	0.07		0.14
0.12	0.09	0.12	0.14		0.08
0.08	0.08	0.14	0.14		
0.14	0.12	0.14	0.13		
0.12	0.11	0.11	0.14		
0.09	0.11	0.14	0.14		
0.12	0.09	0.15	0.13		
0.15	0.12	0.12	0.13		
0.12	0.14	0.15	0.14		
0.14	0.12	0.14	0.13		
0.15	0.12	0.13	0.15		
0.12	0.14	0.13	0.14		
0.11	0.10	0.09	0.08		
0.15	0.09	0.15			
0.13	0.15	0.09			
0.14	0.09	0.08			
0.14	0.12	0.13			
0.14	0.14	0.12			
0.15	0.14				
0.15	0.13				
0.15	0.14				
0.13	0.14				
0.15	0.13				
0.12	0.14				
0.14	0.14				
0.14	0.14				
0.14	0.15				
0.12	0.12				
0.13	0.14				
0.14	0.14				

0.15	0.14				
0.13	0.14				
0.15	0.12				
	0.09				
	0.11				
	0.12				

Spawning, ♀	Post-spawning, ♀
3.78	2.52
4.91	2.81
3.78	2.78
5.70	4.66
5.43	3.57
3.71	4.32
5.53	5.02
4.64	3.24
3.84	2.39
5.12	3.00
4.96	2.55
3.34	3.19
5.50	3.56
2.53	4.83
5.04	3.10
5.23	2.44
4.75	4.04
2.63	2.43
5.25	4.97
3.98	4.22
4.00	5.26
3.89	4.71
4.41	4.39
5.50	4.52
3.75	4.96
3.87	4.45
5.15	4.87
5.34	3.95
5.76	4.71
5.18	3.28

Panel X

>23–40, ♂	>23–40, ♀	41–60, ♂	41–60, ♀	60+, ♂	60+, ♀
2.82	3.78	3.17	4.54	5.02	4.27
3.50	4.91	4.52	3.52	2.68	3.24
5.09	3.78	3.91	3.75	3.82	2.69
5.03	5.70	4.74	4.41	3.94	5.26
4.85	5.43	4.93	3.71	4.59	4.71
4.27	3.71	4.70	3.19	5.29	4.39
4.75	5.53	3.87	3.56	5.25	4.52
3.48	4.64	5.22	4.83	2.94	4.96
2.69	3.84	4.76	3.10	2.93	4.45
5.02	5.12	2.79	2.44	4.35	4.87
3.66	5.09	2.48	4.04	4.79	3.95
4.29	2.53	3.87	2.43	3.01	4.71
3.64	3.06	4.51	4.97	3.99	3.28
3.16	4.45	2.35	4.22		2.69
4.16	4.53	4.47	4.00		5.26
3.38	5.21	4.77	3.89		
4.97	2.99	3.51	4.41		
3.62	4.53	4.94	5.50		
3.09	4.66	4.57	3.75		
3.29	5.23	4.82	3.87		
3.50	2.52	3.47	5.15		
2.86	2.81	4.61	5.34		
4.45	2.78	4.17	5.76		
2.38	4.66	2.96	5.18		
2.75	3.57	4.57	2.55		
3.39	4.32	5.12	3.60		
2.69	5.02	2.76			
3.61	3.24	4.67			
4.35	2.39	3.27			
2.67	3.00	3.14			
4.67	4.96	2.81			
4.52	3.34				
3.85	5.50				
2.94	2.53				
3.30	5.04				
3.94	5.23				
4.68	4.75				
5.23	2.63				
3.71	5.25				
3.31	3.98				
2.35	5.09				
2.47	4.27				
4.91	3.00				
3.48	3.29				

3.72	2.90				
2.67	2.55				
	3.39				
	4.27				
	3.24				

Spawning, ♀	Post-spawning, ♀
141.00	92.00
48.00	27.00
115.00	116.00
152.00	116.00
30.00	112.00
181.00	41.00
38.00	102.00
162.00	48.00
120.00	123.00
183.00	86.00
29.00	15.00
40.00	22.00
47.00	20.00
37.00	8.00
42.00	15.00
44.00	12.00
45.00	23.00
36.00	17.00
32.00	21.00
42.00	17.00
39.00	30.00
42.00	29.00
30.00	29.00
36.00	33.00
40.00	20.00
42.00	21.00
42.00	32.00
34.00	31.00
36.00	22.00
28.00	30.00

Panel AA

>23–40, ♂	>23–40, ♀	41–60, ♂	41–60, ♀	60+, ♂	60+, ♀
54.00	141.00	19.00	38.00	15.00	18.00
41.00	48.00	34.00	20.00	22.00	13.00
38.00	115.00	6.00	27.00	15.00	27.00
26.00	152.00	27.00	10.00	17.00	30.00
54.00	30.00	33.00	41.00	18.00	29.00
45.00	181.00	8.00	22.00	16.00	29.00
65.00	38.00	15.00	20.00	19.00	33.00
31.00	162.00	6.00	8.00	23.00	20.00
43.00	120.00	12.00	15.00	23.00	21.00
28.00	183.00	13.00	12.00	21.00	32.00
30.00	34.00	17.00	23.00	21.00	31.00
50.00	63.00	20.00	17.00	16.00	22.00
26.00	51.00	28.00	21.00	20.00	30.00
30.00	114.00	6.00	17.00		27.00
40.00	117.00	16.00	39.00		30.00
43.00	32.00	16.00	42.00		
57.00	46.00	11.00	30.00		
35.00	117.00	18.00	36.00		
46.00	78.00	13.00	40.00		
29.00	48.00	16.00	42.00		
53.00	92.00	18.00	42.00		
31.00	27.00	12.00	34.00		
58.00	116.00	26.00	36.00		
52.00	116.00	22.00	28.00		
45.00	112.00	9.00	26.00		
59.00	41.00	17.00	24.00		
60.00	102.00	21.00			
40.00	48.00	9.00			
53.00	123.00	19.00			
56.00	86.00	21.00			
20.00	29.00	17.00			
11.00	40.00				
27.00	47.00				
21.00	37.00				
11.00	42.00				
25.00	44.00				
24.00	45.00				
9.00	36.00				
22.00	32.00				
24.00	42.00				
36.00	20.00				
29.00	25.00				
10.00	41.00				
37.00	34.00				
30.00	41.00				

13.00	15.00				
	29.00				
	18.00				
	13.00				

S6 Figure

Panel A

Riverine, ♂	Riverine, ♀	Estuarine, ♂	Estuarine, ♀	Marine, ♂	Marine, ♀	Riverine, both	Estuarine, both
130.00	126.00	130.00	128.00	138.00	139.00	130.00	130.00
131.00	126.00	132.00	128.00	150.00	131.00	131.00	132.00
131.00	128.00	132.00	128.00	147.00	147.00	131.00	132.00
131.00	129.00	133.00	128.00	141.00	146.00	131.00	133.00
133.00	129.00	133.00	129.00	145.00	130.00	133.00	133.00
135.00	129.00	133.00	129.00	143.00	132.00	135.00	133.00
135.00	130.00	133.00	130.00	153.00	147.00	135.00	133.00
135.00	131.00	133.00	131.00	135.00	133.00	135.00	133.00
135.00	131.00	133.00	131.00	145.00	145.00	135.00	133.00
136.00	131.00	133.00	132.00	148.00	137.00	136.00	133.00
136.00	132.00	135.00	133.00	132.00	133.00	136.00	135.00
137.00	133.00	136.00	133.00	132.00	143.00	137.00	136.00
137.00	134.00	137.00	134.00	132.00	142.00	137.00	137.00
137.00	134.00	140.00	134.00	132.00	134.00	137.00	140.00
137.00	135.00	140.00	134.00	136.00	135.00	137.00	140.00
137.00	135.00	140.00	136.00	137.00	130.00	137.00	140.00
137.00	135.00	140.00	136.00	139.00	137.00	137.00	140.00
137.00	135.00	141.00	136.00	141.00	146.00	137.00	141.00
137.00	137.00	141.00	137.00	141.00	144.00	137.00	141.00
138.00	137.00	141.00	138.00	142.00	145.00	138.00	141.00
139.00	138.00	142.00	138.00	144.00	139.00	139.00	142.00
139.00	138.00	143.00	139.00	145.00	137.00	139.00	143.00
140.00	138.00	143.00	139.00	145.00	145.00	140.00	143.00
141.00	138.00	143.00	140.00	138.00	148.00	141.00	143.00
141.00	139.00	144.00	141.00	145.00	139.00	141.00	144.00
141.00	139.00	144.00	141.00	148.00	133.00	141.00	144.00
141.00	139.00	146.00	141.00	149.00	129.00	141.00	146.00
141.00	139.00	146.00	141.00	153.00	138.00	141.00	146.00
142.00	140.00	146.00	142.00	153.00	147.00	142.00	146.00
144.00	140.00	147.00	142.00	155.00	146.00	144.00	147.00
						126.00	128.00
						126.00	128.00
						128.00	128.00
						129.00	128.00
						129.00	129.00
						129.00	129.00
						130.00	130.00
						131.00	131.00
						131.00	131.00
						131.00	132.00
						132.00	133.00
						133.00	133.00
						134.00	134.00
						134.00	134.00

						135.00	134.00
						135.00	136.00
						135.00	136.00
						135.00	136.00
						137.00	137.00
						137.00	138.00
						138.00	138.00
						138.00	139.00
						138.00	139.00
						138.00	140.00
						139.00	141.00
						139.00	141.00
						139.00	141.00
						139.00	141.00
						140.00	142.00
						140.00	142.00

Panel D

Riverine, ♂	Riverine, ♀	Estuarine, ♂	Estuarine, ♀	Marine, ♂	Marine, ♀	Riverine, both	Estuarine, both
102.00	115.00	110.00	115.00	116.00	116.00	102.00	110.00
102.00	110.00	107.00	113.00	115.00	109.00	102.00	107.00
102.00	111.00	112.00	106.00	111.00	115.00	102.00	112.00
103.00	106.00	112.00	111.00	114.00	108.00	103.00	112.00
107.00	113.00	110.00	112.00	114.00	114.00	107.00	110.00
106.00	105.00	109.00	114.00	113.00	114.00	106.00	109.00
106.00	115.00	110.00	111.00	111.00	112.00	106.00	110.00
113.00	111.00	104.00	110.00	116.00	106.00	113.00	104.00
114.00	100.00	102.00	113.00	111.00	113.00	114.00	102.00
104.00	108.00	107.00	110.00	116.00	109.00	104.00	107.00
105.00	112.00	109.00	107.00	112.00	106.00	105.00	109.00
105.00	104.00	111.00	109.00	113.00	115.00	105.00	111.00
103.00	97.00	102.00	113.00	114.00	107.00	103.00	102.00
103.00	111.00	110.00	101.00	113.00	107.00	103.00	110.00
103.00	100.00	108.00	99.00	116.00	116.00	103.00	108.00
107.00	97.00	108.00	112.00	115.00	113.00	107.00	108.00
108.00	106.00	105.00	108.00	103.00	114.00	108.00	105.00
109.00	101.00	110.00	114.00	115.00	111.00	109.00	110.00
109.00	111.00	111.00	109.00	114.00	109.00	109.00	111.00
110.00	111.00	102.00	114.00	114.00	110.00	110.00	102.00
111.00	108.00	114.00	101.00	115.00	105.00	111.00	114.00
111.00	106.00	102.00	110.00	109.00	111.00	111.00	102.00
111.00	104.00	108.00	109.00	111.00	116.00	111.00	108.00
111.00	103.00	111.00	111.00	117.00	115.00	111.00	111.00
113.00	96.00	112.00	103.00	107.00	114.00	113.00	112.00
113.00	110.00	107.00	107.00	108.00	107.00	113.00	107.00
113.00	101.00	109.00	107.00	115.00	109.00	113.00	109.00
113.00	98.00	113.00	101.00	111.00	112.00	113.00	113.00
114.00	107.00	107.00	105.00	112.00	112.00	114.00	107.00
115.00	111.00	112.00	108.00	116.00	113.00	115.00	112.00
						115.00	115.00
						110.00	113.00
						111.00	106.00
						106.00	111.00
						113.00	112.00
						105.00	114.00
						115.00	111.00
						111.00	110.00
						100.00	113.00
						108.00	110.00
						112.00	107.00
						104.00	109.00
						97.00	113.00
						111.00	101.00
						100.00	99.00

						97.00	112.00
						106.00	108.00
						101.00	114.00
						111.00	109.00
						111.00	114.00
						108.00	101.00
						106.00	110.00
						104.00	109.00
						103.00	111.00
						96.00	103.00
						110.00	107.00
						101.00	107.00
						98.00	101.00
						107.00	105.00
						111.00	108.00

Panel G

Riverine, ♂	Riverine, ♀	Estuarine, ♂	Estuarine, ♀	Marine, ♂	Marine, ♀	Riverine, both	Estuarine, both
2.61	2.88	2.73	2.96	2.37	3.03	2.61	2.73
2.80	3.08	3.20	3.10	3.18	3.05	2.80	3.20
2.80	3.05	3.11	2.91	2.68	2.97	2.80	3.11
2.42	3.00	3.12	2.91	2.61	2.89	2.42	3.12
2.44	2.95	2.67	3.06	2.96	3.05	2.44	2.67
3.20	3.12	2.88	3.12	2.95	2.97	3.20	2.88
2.61	3.17	2.24	3.08	2.91	3.02	2.61	2.24
2.39	2.43	2.47	2.90	2.88	2.31	2.39	2.47
2.59	2.74	2.80	3.14	2.51	3.12	2.59	2.80
2.70	2.68	2.23	2.75	2.35	2.89	2.70	2.23
2.50	2.78	2.29	2.15	2.98	2.74	2.50	2.29
2.58	2.56	2.07	2.06	2.50	2.59	2.58	2.07
3.18	3.00	2.81	2.91	3.20	2.97	3.18	2.81
2.74	2.89	2.95	3.06	2.91	2.37	2.74	2.95
2.65	2.86	2.97	3.07	2.70	2.84	2.65	2.97
2.51	3.01	2.98	2.14	2.67	2.69	2.51	2.98
2.56	2.93	2.65	3.09	3.10	2.66	2.56	2.65
2.38	2.72	2.61	3.14	2.08	3.06	2.38	2.61
2.16	2.60	2.80	2.83	2.21	2.58	2.16	2.80
2.74	2.87	2.60	2.76	2.91	2.79	2.74	2.60
2.90	3.07	2.79	2.87	2.19	2.76	2.90	2.79
2.86	3.02	2.90	2.90	2.85	2.85	2.86	2.90
2.73	3.12	2.92	2.79	2.73	2.73	2.73	2.92
2.85	2.92	2.80	3.03	2.86	2.15	2.85	2.80
3.00	2.73	2.25	2.84	2.28	2.20	3.00	2.25
3.07	2.75	2.21	3.06	2.08	2.14	3.07	2.21
3.06	2.99	3.03	2.79	2.94	2.26	3.06	3.03
3.09	2.97	2.87	3.06	3.00	2.19	3.09	2.87
2.88	2.77	2.82	2.94	2.87	2.21	2.88	2.82
2.66	2.78	2.78	3.02	2.92	2.27	2.66	2.78
						2.88	2.96
						3.08	3.10
						3.05	2.91
						3.00	2.91
						2.95	3.06
						3.12	3.12
						3.17	3.08
						2.43	2.90
						2.74	3.14
						2.68	2.75
						2.78	2.15
						2.56	2.06
						3.00	2.91
						2.89	3.06
						2.86	3.07
						3.01	2.14

						2.93	3.09
						2.72	3.14
						2.60	2.83
						2.87	2.76
						3.07	2.87
						3.02	2.90
						3.12	2.79
						2.92	3.03
						2.73	2.84
						2.75	3.06
						2.99	2.79
						2.97	3.06
						2.77	2.94
						2.78	3.02

Panel J

Riverine, ♂	Riverine, ♀	Estuarine, ♂	Estuarine, ♀	Marine, ♂	Marine, ♀	Riverine, both	Estuarine, both

9.22	10.02	8.97	10.01	9.19	9.41	9.22	8.97
9.60	9.94	9.66	9.44	9.02	9.57	9.60	9.66
8.96	9.80	8.78	10.04	9.01	9.62	8.96	8.78
10.07	9.98	9.05	9.80	9.91	9.78	10.07	9.05
9.11	9.03	10.02	9.76	10.05	9.78	9.11	10.02
8.81	9.92	10.03	9.18	9.37	9.68	8.81	10.03
9.19	9.40	10.05	9.87	10.09	10.05	9.19	10.05
8.90	9.60	10.13	9.47	9.98	9.73	8.90	10.13
9.15	9.67	9.85	10.03	9.73	9.79	9.15	9.85
9.63	9.46	9.75	9.75	9.88	9.70	9.63	9.75
9.25	8.94	9.20	9.10	10.11	9.48	9.25	9.20
8.90	10.39	9.59	10.20	9.62	9.33	8.90	9.59
8.94	9.74	9.39	8.97	9.57	9.42	8.94	9.39
8.94	9.20	9.98	9.53	9.96	9.67	8.94	9.98
8.90	8.87	9.29	9.87	10.09	8.79	8.90	9.29
9.33	10.54	9.13	10.19	9.99	9.28	9.33	9.13
9.21	9.50	9.41	9.12	8.92	9.95	9.21	9.41
9.80	9.49	9.41	10.05	9.40	9.37	9.80	9.41
9.72	10.55	9.25	10.11	10.05	9.42	9.72	9.25
9.28	9.63	10.12	8.90	10.00	9.44	9.28	10.12
9.09	9.32	8.75	8.96	10.14	9.22	9.09	8.75
9.96	9.19	10.08	9.58	9.51	9.33	9.96	10.08
9.36	9.16	9.03	9.38	9.49	8.97	9.36	9.03
10.08	9.55	8.78	9.77	8.76	9.71	10.08	8.78
9.11	10.60	10.19	10.16	9.34	9.09	9.11	10.19
9.50	10.01	8.85	9.41	9.72	9.39	9.50	8.85
9.75	9.53	9.39	10.16	9.11	10.16	9.75	9.39
9.54	9.78	9.76	9.12	9.86	9.08	9.54	9.76
9.12	10.52	9.05	9.07	10.13	9.03	9.12	9.05
9.85	9.36	9.49	8.90	9.15	9.83	9.85	9.49
						10.02	10.01
						9.94	9.44
9.34	9.69	9.48	9.60	9.64	9.50	9.80	10.04
0.37	0.48	0.46	0.44	0.42	0.32	9.98	9.80
8.81	8.87	8.75	8.90	8.76	8.79	9.03	9.76
10.08	10.60	10.19	10.20	10.14	10.16	9.92	9.18
						9.40	9.87
	9.52		9.54		9.57	9.60	9.47
	0.46		0.45		0.38	9.67	10.03
						9.46	9.75
					9.54	8.94	9.10
					0.43	10.39	10.20
						9.74	8.97
						9.20	9.53
						8.87	9.87
						10.54	10.19
						9.50	9.12

						9.49	10.05
						10.55	10.11
						9.63	8.90
						9.32	8.96
						9.19	9.58
						9.16	9.38
						9.55	9.77
						10.60	10.16
						10.01	9.41
						9.53	10.16
						9.78	9.12
						10.52	9.07
						9.36	8.90

Panel M

Riverine, ♂	Riverine, ♀	Estuarine, ♂	Estuarine, ♀	Marine, ♂	Marine, ♀	Riverine, both	Estuarine, both
2.65	3.14	3.24	3.37	3.17	2.89	2.65	3.24

2.61	2.89	3.25	3.38	3.21	3.40	2.61	3.25
3.14	2.91	2.86	2.97	3.07	2.73	3.14	2.86
3.23	3.41	3.27	3.17	2.95	3.16	3.23	3.27
3.34	3.39	2.71	2.61	3.36	3.36	3.34	2.71
2.60	3.16	2.99	2.97	2.83	3.19	2.60	2.99
2.96	2.91	2.91	3.25	2.96	2.80	2.96	2.91
2.91	3.41	2.99	3.19	3.24	3.34	2.91	2.99
2.89	3.29	2.64	2.74	3.38	3.03	2.89	2.64
3.23	2.80	3.39	2.93	3.20	3.26	3.23	3.39
2.98	3.35	3.30	2.78	3.28	2.64	2.98	3.30
2.75	3.26	2.75	3.07	2.63	2.79	2.75	2.75
3.36	2.58	2.92	3.12	2.92	2.63	3.36	2.92
2.79	3.35	3.18	3.10	3.12	2.64	2.79	3.18
3.00	3.50	3.32	2.75	2.69	2.80	3.00	3.32
2.64	2.79	2.60	3.08	2.61	2.64	2.64	2.60
3.34	3.17	2.63	3.30	3.33	3.34	3.34	2.63
2.74	2.47	2.71	2.71	2.77	2.63	2.74	2.71
2.97	2.96	2.68	2.92	3.30	2.71	2.97	2.68
3.04	3.40	2.82	2.98	3.13	2.86	3.04	2.82
2.69	3.60	3.03	3.03	3.30	3.24	2.69	3.03
3.06	2.79	2.90	3.18	3.09	2.86	3.06	2.90
2.93	2.95	2.93	3.03	3.12	3.11	2.93	2.93
2.78	3.13	3.09	3.31	3.36	2.90	2.78	3.09
3.31	2.59	2.63	2.71	3.36	3.39	3.31	2.63
2.89	2.74	2.75	3.14	2.80	3.18	2.89	2.75
2.81	2.60	3.32	3.20	2.75	2.85	2.81	3.32
3.31	2.95	2.66	2.66	2.66	2.79	3.31	2.66
2.75	2.56	2.73	3.01	2.87	2.83	2.75	2.73
3.03	2.99	2.90	2.66	3.32	2.66	3.03	2.90
						3.14	3.37
						2.89	3.38
						2.91	2.97
						3.41	3.17
						3.39	2.61
						3.16	2.97
						2.91	3.25
						3.41	3.19
						3.29	2.74
						2.80	2.93
						3.35	2.78
						3.26	3.07
						2.58	3.12
						3.35	3.10
						3.50	2.75
						2.79	3.08
						3.17	3.30
						2.47	2.71

						2.96	2.92
						3.40	2.98
						3.60	3.03
						2.79	3.18
						2.95	3.03
						3.13	3.31
						2.59	2.71
						2.74	3.14
						2.60	3.20
						2.95	2.66
						2.56	3.01
						2.99	2.66

Panel P

Riverine, ♂	Riverine, ♀	Estuarine, ♂	Estuarine, ♀	Marine, ♂	Marine, ♀	Riverine, both	Estuarine, both
7.77	8.03	5.49	6.63	7.00	7.44	7.77	5.49
6.44	7.28	8.05	6.75	8.30	7.75	6.44	8.05

7.28	7.97	5.83	5.05	7.33	7.37	7.28	5.83
8.08	7.85	8.29	7.97	7.92	8.09	8.08	8.29
7.67	8.22	7.79	5.09	5.06	8.00	7.67	7.79
5.93	7.17	6.99	7.29	6.20	7.19	5.93	6.99
5.67	7.45	5.10	7.43	6.91	7.45	5.67	5.10
6.32	7.84	6.05	7.18	7.63	7.73	6.32	6.05
5.62	7.71	5.08	7.69	8.08	8.28	5.62	5.08
5.56	6.39	7.03	7.21	7.91	7.44	5.56	7.03
5.21	7.11	6.01	6.91	7.10	7.90	5.21	6.01
6.19	6.28	5.90	7.05	7.78	7.73	6.19	5.90
7.10	6.88	5.31	6.93	8.37	8.13	7.10	5.31
7.83	6.31	6.27	5.70	7.87	7.48	7.83	6.27
7.58	6.71	5.87	7.78	7.13	7.62	7.58	5.87
6.17	7.28	5.89	6.36	7.49	7.42	6.17	5.89
5.90	6.60	6.55	5.02	7.86	5.82	5.90	6.55
8.15	6.67	6.98	7.81	7.30	6.47	8.15	6.98
5.72	5.47	7.18	8.27	7.10	5.06	5.72	7.18
5.49	5.18	6.28	7.53	6.35	6.38	5.49	6.28
5.70	5.81	5.42	7.51	7.33	6.01	5.70	5.42
8.15	6.61	7.09	5.18	8.43	8.15	8.15	7.09
7.05	6.96	5.59	7.90	7.61	7.69	7.05	5.59
7.44	5.40	6.39	5.41	7.74	7.88	7.44	6.39
6.74	7.04	6.56	6.92	6.49	8.38	6.74	6.56
7.81	6.82	6.81	7.15	7.87	8.28	7.81	6.81
5.72	6.21	6.03	8.00	8.04	6.13	5.72	6.03
5.71	7.90	7.87	6.31	7.10	6.77	5.71	7.87
7.23	6.95	5.37	6.34	7.77	6.15	7.23	5.37
7.69	5.86	6.92	6.00	5.21	7.02	7.69	6.92
						8.03	6.63
						7.28	6.75
						7.97	5.05
						7.85	7.97
						8.22	5.09
						7.17	7.29
						7.45	7.43
						7.84	7.18
						7.71	7.69
						6.39	7.21
						7.11	6.91
						6.28	7.05
						6.88	6.93
						6.31	5.70
						6.71	7.78
						7.28	6.36
						6.60	5.02
						6.67	7.81
						5.47	8.27

						5.18	7.53
						5.81	7.51
						6.61	5.18
						6.96	7.90
						5.40	5.41
						7.04	6.92
						6.82	7.15
						6.21	8.00
						7.90	6.31
						6.95	6.34
						5.86	6.00

Panel S

Riverine, ♂	Riverine, ♀	Estuarine, ♂	Estuarine, ♀	Marine, ♂	Marine, ♀	Riverine, both	Estuarine, both
25.00	32.00	35.00	29.00	30.00	23.00	25.00	35.00
39.00	36.00	42.00	38.00	37.00	29.00	39.00	42.00
26.00	27.00	42.00	29.00	40.00	37.00	26.00	42.00

25.00	31.00	32.00	24.00	45.00	37.00	25.00	32.00
45.00	36.00	47.00	33.00	37.00	36.00	45.00	47.00
29.00	36.00	37.00	38.00	43.00	26.00	29.00	37.00
23.00	33.00	31.00	27.00	25.00	31.00	23.00	31.00
28.00	35.00	22.00	20.00	39.00	21.00	28.00	22.00
20.00	31.00	45.00	26.00	21.00	32.00	20.00	45.00
46.00	28.00	37.00	36.00	31.00	26.00	46.00	37.00
36.00	43.00	41.00	45.00	42.00	40.00	36.00	41.00
33.00	21.00	43.00	41.00	21.00	45.00	33.00	43.00
43.00	22.00	23.00	31.00	45.00	41.00	43.00	23.00
39.00	36.00	47.00	38.00	43.00	37.00	39.00	47.00
22.00	26.00	37.00	43.00	24.00	42.00	22.00	37.00
40.00	25.00	36.00	42.00	29.00	43.00	40.00	36.00
42.00	39.00	32.00	30.00	33.00	30.00	42.00	32.00
25.00	39.00	37.00	26.00	44.00	32.00	25.00	37.00
46.00	22.00	39.00	24.00	42.00	30.00	46.00	39.00
21.00	22.00	23.00	33.00	38.00	45.00	21.00	23.00
34.00	29.00	20.00	39.00	47.00	33.00	34.00	20.00
46.00	47.00	43.00	35.00	29.00	32.00	46.00	43.00
26.00	25.00	43.00	42.00	43.00	33.00	26.00	43.00
44.00	40.00	40.00	43.00	26.00	36.00	44.00	40.00
41.00	32.00	29.00	34.00	35.00	36.00	41.00	29.00
21.00	35.00	27.00	39.00	39.00	30.00	21.00	27.00
37.00	43.00	26.00	35.00	44.00	35.00	37.00	26.00
32.00	46.00	27.00	42.00	40.00	31.00	32.00	27.00
40.00	40.00	24.00	37.00	21.00	37.00	40.00	24.00
26.00	25.00	38.00	32.00	44.00	21.00	26.00	38.00
						32.00	29.00
						36.00	38.00
						27.00	29.00
						31.00	24.00
						36.00	33.00
						36.00	38.00
						33.00	27.00
						35.00	20.00
						31.00	26.00
						28.00	36.00
						43.00	45.00
						21.00	41.00
						22.00	31.00
						36.00	38.00
						26.00	43.00
						25.00	42.00
						39.00	30.00
						39.00	26.00
						22.00	24.00
						22.00	33.00

						29.00	39.00
						47.00	35.00
						25.00	42.00
						40.00	43.00
						32.00	34.00
						35.00	39.00
						43.00	35.00
						46.00	42.00
						40.00	37.00
						25.00	32.00

Marine, both	All, ♂	All, ♀
138.00	130.00	126.00
150.00	131.00	126.00
147.00	131.00	128.00
141.00	131.00	129.00
145.00	133.00	129.00
143.00	135.00	129.00
153.00	135.00	130.00
135.00	135.00	131.00
145.00	135.00	131.00
148.00	136.00	131.00
132.00	136.00	132.00
132.00	137.00	133.00
132.00	137.00	134.00
132.00	137.00	134.00
136.00	137.00	135.00
137.00	137.00	135.00
139.00	137.00	135.00
141.00	137.00	135.00
141.00	137.00	137.00
142.00	138.00	137.00
144.00	139.00	138.00
145.00	139.00	138.00
145.00	140.00	138.00
138.00	141.00	138.00
145.00	141.00	139.00
148.00	141.00	139.00
149.00	141.00	139.00
153.00	141.00	139.00
153.00	142.00	140.00
155.00	144.00	140.00
139.00	130.00	128.00
131.00	132.00	128.00
147.00	132.00	128.00
146.00	133.00	128.00
130.00	133.00	129.00
132.00	133.00	129.00
147.00	133.00	130.00
133.00	133.00	131.00
145.00	133.00	131.00
137.00	133.00	132.00
133.00	135.00	133.00
143.00	136.00	133.00
142.00	137.00	134.00
134.00	140.00	134.00

Panel B

Pre-spawning, ♂	Spawning, ♂	Post-spawning, ♂	Pre-spawning, ♀
136.00	130.00	139.00	132.00
137.00	131.00	139.00	133.00
137.00	131.00	140.00	134.00
137.00	131.00	141.00	134.00
137.00	133.00	141.00	135.00
137.00	135.00	141.00	135.00
137.00	135.00	141.00	135.00
137.00	135.00	141.00	135.00
137.00	135.00	142.00	137.00
138.00	136.00	144.00	137.00
135.00	130.00	142.00	133.00
136.00	132.00	143.00	133.00
137.00	132.00	143.00	134.00
140.00	133.00	143.00	134.00
140.00	133.00	144.00	134.00
140.00	133.00	144.00	136.00
140.00	133.00	146.00	136.00
141.00	133.00	146.00	136.00
141.00	133.00	146.00	137.00
141.00	133.00	147.00	138.00
132.00	138.00	144.00	133.00
132.00	150.00	145.00	143.00
132.00	147.00	145.00	142.00
132.00	141.00	138.00	134.00
136.00	145.00	145.00	135.00
137.00	143.00	148.00	130.00
139.00	153.00	149.00	137.00
141.00	135.00	153.00	146.00
141.00	145.00	153.00	144.00
142.00	148.00	155.00	145.00

135.00	140.00	134.00
130.00	140.00	136.00
137.00	140.00	136.00
146.00	141.00	136.00
144.00	141.00	137.00
145.00	141.00	138.00
139.00	142.00	138.00
137.00	143.00	139.00
145.00	143.00	139.00
148.00	143.00	140.00
139.00	144.00	141.00
133.00	144.00	141.00
129.00	146.00	141.00
138.00	146.00	141.00
147.00	146.00	142.00
146.00	147.00	142.00
	138.00	139.00
	150.00	131.00
	147.00	147.00
	141.00	146.00
	145.00	130.00
	143.00	132.00
	153.00	147.00
	135.00	133.00
	145.00	145.00
	148.00	137.00
	132.00	133.00
	132.00	143.00
	132.00	142.00
	132.00	134.00
	136.00	135.00
	137.00	130.00
	139.00	137.00
	141.00	146.00
	141.00	144.00
	142.00	145.00
	144.00	139.00
	145.00	137.00
	145.00	145.00
	138.00	148.00
	145.00	139.00
	148.00	133.00
	149.00	129.00
	153.00	138.00
	153.00	147.00
	155.00	146.00

Marine, both	All, ♂	All, ♀
116.00	102.00	115.00
115.00	102.00	110.00
111.00	102.00	111.00
114.00	103.00	106.00
114.00	107.00	113.00
113.00	106.00	105.00
111.00	106.00	115.00
116.00	113.00	111.00
111.00	114.00	100.00
116.00	104.00	108.00
112.00	105.00	112.00
113.00	105.00	104.00
114.00	103.00	97.00
113.00	103.00	111.00
116.00	103.00	100.00
115.00	107.00	97.00
103.00	108.00	106.00
115.00	109.00	101.00
114.00	109.00	111.00
114.00	110.00	111.00
115.00	111.00	108.00
109.00	111.00	106.00
111.00	111.00	104.00
117.00	111.00	103.00
107.00	113.00	96.00
108.00	113.00	110.00
115.00	113.00	101.00
111.00	113.00	98.00
112.00	114.00	107.00
116.00	115.00	111.00
116.00	110.00	115.00
109.00	107.00	113.00
115.00	112.00	106.00
108.00	112.00	111.00
114.00	110.00	112.00
114.00	109.00	114.00
112.00	110.00	111.00
106.00	104.00	110.00
113.00	102.00	113.00
109.00	107.00	110.00
106.00	109.00	107.00
115.00	111.00	109.00
107.00	102.00	113.00
107.00	110.00	101.00
116.00	108.00	99.00

Panel E

Pre-spawning, ♂	Spawning, ♂	Post-spawning, ♂	Pre-spawning, ♀
105.00	102.00	111.00	112.00
105.00	102.00	111.00	104.00
103.00	102.00	111.00	97.00
103.00	103.00	111.00	111.00
103.00	107.00	113.00	100.00
107.00	106.00	113.00	97.00
108.00	106.00	113.00	106.00
109.00	113.00	113.00	101.00
109.00	114.00	114.00	111.00
110.00	104.00	115.00	111.00
109.00	110.00	114.00	107.00
111.00	107.00	102.00	109.00
102.00	112.00	108.00	113.00
110.00	112.00	111.00	101.00
108.00	110.00	112.00	99.00
108.00	109.00	107.00	112.00
105.00	110.00	109.00	108.00
110.00	104.00	113.00	114.00
111.00	102.00	107.00	109.00
102.00	107.00	112.00	114.00
112.00	116.00	115.00	106.00
113.00	115.00	109.00	115.00
114.00	111.00	111.00	107.00
113.00	114.00	117.00	107.00
116.00	114.00	107.00	116.00
115.00	113.00	108.00	113.00
103.00	111.00	115.00	114.00
115.00	116.00	111.00	111.00
114.00	111.00	112.00	109.00
114.00	116.00	116.00	110.00

113.00	108.00	112.00
114.00	105.00	108.00
111.00	110.00	114.00
109.00	111.00	109.00
110.00	102.00	114.00
105.00	114.00	101.00
111.00	102.00	110.00
116.00	108.00	109.00
115.00	111.00	111.00
114.00	112.00	103.00
107.00	107.00	107.00
109.00	109.00	107.00
112.00	113.00	101.00
112.00	107.00	105.00
113.00	112.00	108.00
	116.00	116.00
	115.00	109.00
	111.00	115.00
	114.00	108.00
	114.00	114.00
	113.00	114.00
	111.00	112.00
	116.00	106.00
	111.00	113.00
	116.00	109.00
	112.00	106.00
	113.00	115.00
	114.00	107.00
	113.00	107.00
	116.00	116.00
	115.00	113.00
	103.00	114.00
	115.00	111.00
	114.00	109.00
	114.00	110.00
	115.00	105.00
	109.00	111.00
	111.00	116.00
	117.00	115.00
	107.00	114.00
	108.00	107.00
	115.00	109.00
	111.00	112.00
	112.00	112.00
	116.00	113.00

Panel H

Marine, both	All, ♂	All, ♀
2.37	2.61	2.88
3.18	2.80	3.08
2.68	2.80	3.05
2.61	2.42	3.00
2.96	2.44	2.95
2.95	3.20	3.12
2.91	2.61	3.17
2.88	2.39	2.43
2.51	2.59	2.74
2.35	2.70	2.68
2.98	2.50	2.78
2.50	2.58	2.56
3.20	3.18	3.00
2.91	2.74	2.89
2.70	2.65	2.86
2.67	2.51	3.01
3.10	2.56	2.93
2.08	2.38	2.72
2.21	2.16	2.60
2.91	2.74	2.87
2.19	2.90	3.07
2.85	2.86	3.02
2.73	2.73	3.12
2.86	2.85	2.92
2.28	3.00	2.73
2.08	3.07	2.75
2.94	3.06	2.99
3.00	3.09	2.97
2.87	2.88	2.77
2.92	2.66	2.78
3.03	2.73	2.96
3.05	3.20	3.10
2.97	3.11	2.91
2.89	3.12	2.91
3.05	2.67	3.06
2.97	2.88	3.12
3.02	2.24	3.08
2.31	2.47	2.90
3.12	2.80	3.14
2.89	2.23	2.75
2.74	2.29	2.15
2.59	2.07	2.06
2.97	2.81	2.91
2.37	2.95	3.06
2.84	2.97	3.07
2.69	2.98	2.14

Pre-spawning, ♂	Spawning, ♂	Post-spawning, ♂	Pre-spawning, ♀
2.50	2.61	2.90	2.78
2.58	2.80	2.86	2.56
3.18	2.80	2.73	3.00
2.74	2.42	2.85	2.89
2.65	2.44	3.00	2.86
2.51	3.20	3.07	3.01
2.56	2.61	3.06	2.93
2.38	2.39	3.09	2.72
2.16	2.59	2.88	2.60
2.74	2.70	2.66	2.87
2.29	2.73	2.79	2.15
2.07	3.20	2.90	2.06
2.81	3.11	2.92	2.91
2.95	3.12	2.80	3.06
2.97	2.67	2.25	3.07
2.98	2.88	2.21	2.14
2.65	2.24	3.03	3.09
2.61	2.47	2.87	3.14
2.80	2.80	2.82	2.83
2.60	2.23	2.78	2.76
2.98	2.37	2.19	2.74
2.50	3.18	2.85	2.59
3.20	2.68	2.73	2.97
2.91	2.61	2.86	2.37
2.70	2.96	2.28	2.84
2.67	2.95	2.08	2.69
3.10	2.91	2.94	2.66
2.08	2.88	3.00	3.06
2.21	2.51	2.87	2.58
2.91	2.35	2.92	2.79

2.66	2.65	3.09
3.06	2.61	3.14
2.58	2.80	2.83
2.79	2.60	2.76
2.76	2.79	2.87
2.85	2.90	2.90
2.73	2.92	2.79
2.15	2.80	3.03
2.20	2.25	2.84
2.14	2.21	3.06
2.26	3.03	2.79
2.19	2.87	3.06
2.21	2.82	2.94
2.27	2.78	3.02
	2.37	3.03
	3.18	3.05
	2.68	2.97
	2.61	2.89
	2.96	3.05
	2.95	2.97
	2.91	3.02
	2.88	2.31
	2.51	3.12
	2.35	2.89
	2.98	2.74
	2.50	2.59
	3.20	2.97
	2.91	2.37
	2.70	2.84
	2.67	2.69
	3.10	2.66
	2.08	3.06
	2.21	2.58
	2.91	2.79
	2.19	2.76
	2.85	2.85
	2.73	2.73
	2.86	2.15
	2.28	2.20
	2.08	2.14
	2.94	2.26
	3.00	2.19
	2.87	2.21
	2.92	2.27

Marine, both	All, ♂	All, ♀

Panel K

Pre-spawning, ♂	Spawning, ♂	Post-spawning, ♂	Pre-spawning, ♀

9.19	9.22	10.02
9.02	9.60	9.94
9.01	8.96	9.80
9.91	10.07	9.98
10.05	9.11	9.03
9.37	8.81	9.92
10.09	9.19	9.40
9.98	8.90	9.60
9.73	9.15	9.67
9.88	9.63	9.46
10.11	9.25	8.94
9.62	8.90	10.39
9.57	8.94	9.74
9.96	8.94	9.20
10.09	8.90	8.87
9.99	9.33	10.54
8.92	9.21	9.50
9.40	9.80	9.49
10.05	9.72	10.55
10.00	9.28	9.63
10.14	9.09	9.32
9.51	9.96	9.19
9.49	9.36	9.16
8.76	10.08	9.55
9.34	9.11	10.60
9.72	9.50	10.01
9.11	9.75	9.53
9.86	9.54	9.78
10.13	9.12	10.52
9.15	9.85	9.36
9.41	8.97	10.01
9.57	9.66	9.44
9.62	8.78	10.04
9.78	9.05	9.80
9.78	10.02	9.76
9.68	10.03	9.18
10.05	10.05	9.87
9.73	10.13	9.47
9.79	9.85	10.03
9.70	9.75	9.75
9.48	9.20	9.10
9.33	9.59	10.20
9.42	9.39	8.97
9.67	9.98	9.53
8.79	9.29	9.87
9.28	9.13	10.19
9.95	9.41	9.12

9.25	9.22	9.09	8.94
8.90	9.60	9.96	10.39
8.94	8.96	9.36	9.74
8.94	10.07	10.08	9.20
8.90	9.11	9.11	8.87
9.33	8.81	9.50	10.54
9.21	9.19	9.75	9.50
9.80	8.90	9.54	9.49
9.72	9.15	9.12	10.55
9.28	9.63	9.85	9.63
9.20	8.97	8.75	9.10
9.59	9.66	10.08	10.20
9.39	8.78	9.03	8.97
9.98	9.05	8.78	9.53
9.29	10.02	10.19	9.87
9.13	10.03	8.85	10.19
9.41	10.05	9.39	9.12
9.41	10.13	9.76	10.05
9.25	9.85	9.05	10.11
10.12	9.75	9.49	8.90
10.11	9.19	10.14	9.48
9.62	9.02	9.51	9.33
9.57	9.01	9.49	9.42
9.96	9.91	8.76	9.67
10.09	10.05	9.34	8.79
9.99	9.37	9.72	9.28
8.92	10.09	9.11	9.95
9.40	9.98	9.86	9.37
10.05	9.73	10.13	9.42
10.00	9.88	9.15	9.44

9.37	9.41	10.05
9.42	9.25	10.11
9.44	10.12	8.90
9.22	8.75	8.96
9.33	10.08	9.58
8.97	9.03	9.38
9.71	8.78	9.77
9.09	10.19	10.16
9.39	8.85	9.41
10.16	9.39	10.16
9.08	9.76	9.12
9.03	9.05	9.07
9.83	9.49	8.90
	9.19	9.41
	9.02	9.57
	9.01	9.62
	9.91	9.78
	10.05	9.78
	9.37	9.68
	10.09	10.05
	9.98	9.73
	9.73	9.79
	9.88	9.70
	10.11	9.48
	9.62	9.33
	9.57	9.42
	9.96	9.67
	10.09	8.79
	9.99	9.28
	8.92	9.95
	9.40	9.37
	10.05	9.42
	10.00	9.44
	10.14	9.22
	9.51	9.33
	9.49	8.97
	8.76	9.71
	9.34	9.09
	9.72	9.39
	9.11	10.16
	9.86	9.08
	10.13	9.03
	9.15	9.83

Marine, both	All, ♂	All, ♀
3.17	2.65	3.14

Panel N

Pre-spawning, ♂	Spawning, ♂	Post-spawning, ♂	Pre-spawning, ♀
2.98	2.65	2.69	3.35

3.21	2.61	2.89
3.07	3.14	2.91
2.95	3.23	3.41
3.36	3.34	3.39
2.83	2.60	3.16
2.96	2.96	2.91
3.24	2.91	3.41
3.38	2.89	3.29
3.20	3.23	2.80
3.28	2.98	3.35
2.63	2.75	3.26
2.92	3.36	2.58
3.12	2.79	3.35
2.69	3.00	3.50
2.61	2.64	2.79
3.33	3.34	3.17
2.77	2.74	2.47
3.30	2.97	2.96
3.13	3.04	3.40
3.30	2.69	3.60
3.09	3.06	2.79
3.12	2.93	2.95
3.36	2.78	3.13
3.36	3.31	2.59
2.80	2.89	2.74
2.75	2.81	2.60
2.66	3.31	2.95
2.87	2.75	2.56
3.32	3.03	2.99
2.89	3.24	3.37
3.40	3.25	3.38
2.73	2.86	2.97
3.16	3.27	3.17
3.36	2.71	2.61
3.19	2.99	2.97
2.80	2.91	3.25
3.34	2.99	3.19
3.03	2.64	2.74
3.26	3.39	2.93
2.64	3.30	2.78
2.79	2.75	3.07
2.63	2.92	3.12
2.64	3.18	3.10
2.80	3.32	2.75
2.64	2.60	3.08
3.34	2.63	3.30
2.63	2.71	2.71

2.75	2.61	3.06	3.26
3.36	3.14	2.93	2.58
2.79	3.23	2.78	3.35
3.00	3.34	3.31	3.50
2.64	2.60	2.89	2.79
3.34	2.96	2.81	3.17
2.74	2.91	3.31	2.47
2.97	2.89	2.75	2.96
3.04	3.23	3.03	3.40
3.30	3.24	3.03	2.78
2.75	3.25	2.90	3.07
2.92	2.86	2.93	3.12
3.18	3.27	3.09	3.10
3.32	2.71	2.63	2.75
2.60	2.99	2.75	3.08
2.63	2.91	3.32	3.30
2.71	2.99	2.66	2.71
2.68	2.64	2.73	2.92
2.82	3.39	2.90	2.98
3.28	3.17	3.30	2.64
2.63	3.21	3.09	2.79
2.92	3.07	3.12	2.63
3.12	2.95	3.36	2.64
2.69	3.36	3.36	2.80
2.61	2.83	2.80	2.64
3.33	2.96	2.75	3.34
2.77	3.24	2.66	2.63
3.30	3.38	2.87	2.71
3.13	3.20	3.32	2.86

2.71	2.68	2.92
2.86	2.82	2.98
3.24	3.03	3.03
2.86	2.90	3.18
3.11	2.93	3.03
2.90	3.09	3.31
3.39	2.63	2.71
3.18	2.75	3.14
2.85	3.32	3.20
2.79	2.66	2.66
2.83	2.73	3.01
2.66	2.90	2.66
	3.17	2.89
	3.21	3.40
	3.07	2.73
	2.95	3.16
	3.36	3.36
	2.83	3.19
	2.96	2.80
	3.24	3.34
	3.38	3.03
	3.20	3.26
	3.28	2.64
	2.63	2.79
	2.92	2.63
	3.12	2.64
	2.69	2.80
	2.61	2.64
	3.33	3.34
	2.77	2.63
	3.30	2.71
	3.13	2.86
	3.30	3.24
	3.09	2.86
	3.12	3.11
	3.36	2.90
	3.36	3.39
	2.80	3.18
	2.75	2.85
	2.66	2.79
	2.87	2.83
	3.32	2.66

Marine, both	All, ♂	All, ♀
7.00	7.77	8.03
8.30	6.44	7.28

Panel Q

Pre-spawning, ♂	Spawning, ♂	Post-spawning, ♂	Pre-spawning, ♀
5.21	7.77	5.70	7.11
6.19	6.44	8.15	6.28

7.33	7.28	7.97
7.92	8.08	7.85
5.06	7.67	8.22
6.20	5.93	7.17
6.91	5.67	7.45
7.63	6.32	7.84
8.08	5.62	7.71
7.91	5.56	6.39
7.10	5.21	7.11
7.78	6.19	6.28
8.37	7.10	6.88
7.87	7.83	6.31
7.13	7.58	6.71
7.49	6.17	7.28
7.86	5.90	6.60
7.30	8.15	6.67
7.10	5.72	5.47
6.35	5.49	5.18
7.33	5.70	5.81
8.43	8.15	6.61
7.61	7.05	6.96
7.74	7.44	5.40
6.49	6.74	7.04
7.87	7.81	6.82
8.04	5.72	6.21
7.10	5.71	7.90
7.77	7.23	6.95
5.21	7.69	5.86
7.44	5.49	6.63
7.75	8.05	6.75
7.37	5.83	5.05
8.09	8.29	7.97
8.00	7.79	5.09
7.19	6.99	7.29
7.45	5.10	7.43
7.73	6.05	7.18
8.28	5.08	7.69
7.44	7.03	7.21
7.90	6.01	6.91
7.73	5.90	7.05
8.13	5.31	6.93
7.48	6.27	5.70
7.62	5.87	7.78
7.42	5.89	6.36
5.82	6.55	5.02
6.47	6.98	7.81
5.06	7.18	8.27

7.10	7.28	7.05	6.88
7.83	8.08	7.44	6.31
7.58	7.67	6.74	6.71
6.17	5.93	7.81	7.28
5.90	5.67	5.72	6.60
8.15	6.32	5.71	6.67
5.72	5.62	7.23	5.47
5.49	5.56	7.69	5.18
6.01	5.49	5.42	6.91
5.90	8.05	7.09	7.05
5.31	5.83	5.59	6.93
6.27	8.29	6.39	5.70
5.87	7.79	6.56	7.78
5.89	6.99	6.81	6.36
6.55	5.10	6.03	5.02
6.98	6.05	7.87	7.81
7.18	5.08	5.37	8.27
6.28	7.03	6.92	7.53
7.10	7.00	7.33	7.90
7.78	8.30	8.43	7.73
8.37	7.33	7.61	8.13
7.87	7.92	7.74	7.48
7.13	5.06	6.49	7.62
7.49	6.20	7.87	7.42
7.86	6.91	8.04	5.82
7.30	7.63	7.10	6.47
7.10	8.08	7.77	5.06
6.35	7.91	5.21	6.38

6.38	6.28	7.53
6.01	5.42	7.51
8.15	7.09	5.18
7.69	5.59	7.90
7.88	6.39	5.41
8.38	6.56	6.92
8.28	6.81	7.15
6.13	6.03	8.00
6.77	7.87	6.31
6.15	5.37	6.34
7.02	6.92	6.00
	7.00	7.44
	8.30	7.75
	7.33	7.37
	7.92	8.09
	5.06	8.00
	6.20	7.19
	6.91	7.45
	7.63	7.73
	8.08	8.28
	7.91	7.44
	7.10	7.90
	7.78	7.73
	8.37	8.13
	7.87	7.48
	7.13	7.62
	7.49	7.42
	7.86	5.82
	7.30	6.47
	7.10	5.06
	6.35	6.38
	7.33	6.01
	8.43	8.15
	7.61	7.69
	7.74	7.88
	6.49	8.38
	7.87	8.28
	8.04	6.13
	7.10	6.77
	7.77	6.15
	5.21	7.02

Marine, both	All, ♂	All, ♀
30.00	25.00	32.00
37.00	39.00	36.00
40.00	26.00	27.00

Panel T

Pre-spawning, ♂	Spawning, ♂	Post-spawning, ♂	Pre-spawning, ♀
36.00	25.00	34.00	43.00
33.00	39.00	46.00	21.00
43.00	26.00	26.00	22.00

45.00	25.00	31.00
37.00	45.00	36.00
43.00	29.00	36.00
25.00	23.00	33.00
39.00	28.00	35.00
21.00	20.00	31.00
31.00	46.00	28.00
42.00	36.00	43.00
21.00	33.00	21.00
45.00	43.00	22.00
43.00	39.00	36.00
24.00	22.00	26.00
29.00	40.00	25.00
33.00	42.00	39.00
44.00	25.00	39.00
42.00	46.00	22.00
38.00	21.00	22.00
47.00	34.00	29.00
29.00	46.00	47.00
43.00	26.00	25.00
26.00	44.00	40.00
35.00	41.00	32.00
39.00	21.00	35.00
44.00	37.00	43.00
40.00	32.00	46.00
21.00	40.00	40.00
44.00	26.00	25.00
23.00	35.00	29.00
29.00	42.00	38.00
37.00	42.00	29.00
37.00	32.00	24.00
36.00	47.00	33.00
26.00	37.00	38.00
31.00	31.00	27.00
21.00	22.00	20.00
32.00	45.00	26.00
26.00	37.00	36.00
40.00	41.00	45.00
45.00	43.00	41.00
41.00	23.00	31.00
37.00	47.00	38.00
42.00	37.00	43.00
43.00	36.00	42.00
30.00	32.00	30.00
32.00	37.00	26.00
30.00	39.00	24.00
45.00	23.00	33.00

39.00	25.00	44.00	36.00
22.00	45.00	41.00	26.00
40.00	29.00	21.00	25.00
42.00	23.00	37.00	39.00
25.00	28.00	32.00	39.00
46.00	20.00	40.00	22.00
21.00	46.00	26.00	22.00
41.00	35.00	20.00	45.00
43.00	42.00	43.00	41.00
23.00	42.00	43.00	31.00
47.00	32.00	40.00	38.00
37.00	47.00	29.00	43.00
36.00	37.00	27.00	42.00
32.00	31.00	26.00	30.00
37.00	22.00	27.00	26.00
39.00	45.00	24.00	24.00
23.00	37.00	38.00	33.00
42.00	30.00	47.00	40.00
21.00	37.00	29.00	45.00
45.00	40.00	43.00	41.00
43.00	45.00	26.00	37.00
24.00	37.00	35.00	42.00
29.00	43.00	39.00	43.00
33.00	25.00	44.00	30.00
44.00	39.00	40.00	32.00
42.00	21.00	21.00	30.00
38.00	31.00	44.00	45.00

33.00	20.00	39.00
32.00	43.00	35.00
33.00	43.00	42.00
36.00	40.00	43.00
36.00	29.00	34.00
30.00	27.00	39.00
35.00	26.00	35.00
31.00	27.00	42.00
37.00	24.00	37.00
21.00	38.00	32.00
	30.00	23.00
	37.00	29.00
	40.00	37.00
	45.00	37.00
	37.00	36.00
	43.00	26.00
	25.00	31.00
	39.00	21.00
	21.00	32.00
	31.00	26.00
	42.00	40.00
	21.00	45.00
	45.00	41.00
	43.00	37.00
	24.00	42.00
	29.00	43.00
	33.00	30.00
	44.00	32.00
	42.00	30.00
	38.00	45.00
	47.00	33.00
	29.00	32.00
	43.00	33.00
	26.00	36.00
	35.00	36.00
	39.00	30.00
	44.00	35.00
	40.00	31.00
	21.00	37.00
	44.00	21.00

Spawning, ♀	Post-spawning, ♀
126.00	138.00
126.00	138.00
128.00	138.00
129.00	138.00
129.00	139.00
129.00	139.00
130.00	139.00
131.00	139.00
131.00	140.00
131.00	140.00
128.00	138.00
128.00	139.00
128.00	139.00
128.00	140.00
129.00	141.00
129.00	141.00
130.00	141.00
131.00	141.00
131.00	142.00
132.00	142.00
139.00	139.00
131.00	137.00
147.00	145.00
146.00	148.00
130.00	139.00
132.00	133.00
147.00	129.00
133.00	138.00
145.00	147.00
137.00	146.00

Panel C

>23–40, ♂	>23–40, ♀	41–60, ♂	41–60, ♀	60+, ♂	60+, ♀
130.00	126.00	136.00	133.00	132.00	146.00
131.00	126.00	137.00	134.00	136.00	144.00
131.00	128.00	140.00	134.00	137.00	145.00
131.00	129.00	140.00	134.00	139.00	139.00
133.00	129.00	140.00	136.00	145.00	137.00
135.00	129.00	143.00	139.00	145.00	145.00
135.00	130.00	143.00	139.00	138.00	148.00
135.00	131.00	143.00	140.00	145.00	139.00
135.00	131.00	144.00	141.00	148.00	133.00
136.00	131.00	144.00	141.00	149.00	129.00
136.00	132.00	146.00	141.00	153.00	138.00
137.00	133.00	146.00	141.00	153.00	147.00
137.00	134.00	146.00	142.00	155.00	146.00
137.00	134.00	147.00	142.00		145.00
137.00	135.00	138.00	139.00		139.00
137.00	135.00	150.00	131.00		
137.00	135.00	147.00	147.00		
137.00	135.00	141.00	146.00		
137.00	137.00	145.00	130.00		
138.00	137.00	143.00	132.00		
139.00	138.00	153.00	147.00		
139.00	138.00	135.00	133.00		
140.00	138.00	145.00	145.00		
141.00	138.00	148.00	137.00		
141.00	139.00	132.00	133.00		
141.00	139.00	132.00	143.00		
141.00	139.00	132.00			
141.00	139.00	141.00			
142.00	140.00	141.00			
144.00	140.00	142.00			
130.00	128.00	144.00			
132.00	128.00				
132.00	128.00				
133.00	128.00				
133.00	129.00				
133.00	129.00				
133.00	130.00				
133.00	131.00				
133.00	131.00				
133.00	132.00				
135.00	133.00				
140.00	136.00				
141.00	136.00				
141.00	137.00				

141.00	138.00				
142.00	138.00				
	142.00				
	146.00				
	144.00				

Spawning, ♀	Post-spawning, ♀
115.00	108.00
110.00	106.00
111.00	104.00
106.00	103.00
113.00	96.00
105.00	110.00
115.00	101.00
111.00	98.00
100.00	107.00
108.00	111.00
115.00	101.00
113.00	110.00
106.00	109.00
111.00	111.00
112.00	103.00
114.00	107.00
111.00	107.00
110.00	101.00
113.00	105.00
110.00	108.00
116.00	105.00
109.00	111.00
115.00	116.00
108.00	115.00
114.00	114.00
114.00	107.00
112.00	109.00
106.00	112.00
113.00	112.00
109.00	113.00

Panel F

>23–40, ♂	>23–40, ♀	41–60, ♂	41–60, ♀	60+, ♂	60+, ♀
102.00	115.00	111.00	109.00	113.00	111.00
102.00	110.00	102.00	113.00	116.00	109.00
102.00	111.00	110.00	101.00	115.00	110.00
103.00	106.00	108.00	99.00	103.00	105.00
107.00	113.00	108.00	112.00	109.00	111.00
106.00	105.00	102.00	110.00	111.00	116.00
106.00	115.00	108.00	109.00	117.00	115.00
113.00	111.00	111.00	111.00	107.00	114.00
114.00	100.00	112.00	103.00	108.00	107.00
104.00	108.00	107.00	107.00	115.00	109.00
105.00	112.00	109.00	107.00	111.00	112.00
105.00	104.00	113.00	101.00	112.00	112.00
103.00	97.00	107.00	105.00	116.00	113.00
103.00	111.00	112.00	108.00		110.00
103.00	100.00	116.00	116.00		105.00
107.00	97.00	115.00	109.00		
108.00	106.00	111.00	115.00		
109.00	101.00	114.00	108.00		
109.00	111.00	114.00	114.00		
110.00	111.00	113.00	114.00		
111.00	108.00	111.00	112.00		
111.00	106.00	116.00	106.00		
111.00	104.00	111.00	113.00		
111.00	103.00	116.00	109.00		
113.00	96.00	112.00	106.00		
113.00	110.00	113.00	115.00		
113.00	101.00	114.00			
113.00	98.00	115.00			
114.00	107.00	114.00			
115.00	111.00	114.00			
110.00	115.00	115.00			
107.00	113.00				
112.00	106.00				
112.00	111.00				
110.00	112.00				
109.00	114.00				
110.00	111.00				
104.00	110.00				
102.00	113.00				
107.00	110.00				
109.00	107.00				
105.00	108.00				
110.00	114.00				
111.00	109.00				
102.00	114.00				

114.00	101.00				
	107.00				
	111.00				
	109.00				

Panel I

Spawning, ♀	Post-spawning, ♀
2.88	3.07
3.08	3.02
3.05	3.12
3.00	2.92
2.95	2.73
3.12	2.75
3.17	2.99
2.43	2.97
2.74	2.77
2.68	2.78
2.96	2.87
3.10	2.90
2.91	2.79
2.91	3.03
3.06	2.84
3.12	3.06
3.08	2.79
2.90	3.06
3.14	2.94
2.75	3.02
3.03	2.76
3.05	2.85
2.97	2.73
2.89	2.15
3.05	2.20
2.97	2.14
3.02	2.26
2.31	2.19
3.12	2.21
2.89	2.27

>23–40, ♂	>23–40, ♀	41–60, ♂	41–60, ♀	60+, ♂	60+, ♀
2.61	2.88	2.07	2.06	2.91	3.06
2.80	3.08	2.81	2.91	2.70	2.58
2.80	3.05	2.95	3.06	2.67	2.79
2.42	3.00	2.97	3.07	3.10	2.76
2.44	2.95	2.98	2.14	2.85	2.85
3.20	3.12	2.90	2.90	2.73	2.73
2.61	3.17	2.92	2.79	2.86	2.15
2.39	2.43	2.80	3.03	2.28	2.20
2.59	2.74	2.25	2.84	2.08	2.14
2.70	2.68	2.21	3.06	2.94	2.26
2.50	2.78	3.03	2.79	3.00	2.19
2.58	2.56	2.87	3.06	2.87	2.21
3.18	3.00	2.82	2.94	2.92	2.27
2.74	2.89	2.78	3.02		2.79
2.65	2.86	2.37	3.03		2.76
2.51	3.01	3.18	3.05		
2.56	2.93	2.68	2.97		
2.38	2.72	2.61	2.89		
2.16	2.60	2.96	3.05		
2.74	2.87	2.95	2.97		
2.90	3.07	2.91	3.02		
2.86	3.02	2.88	2.31		
2.73	3.12	2.51	3.12		
2.85	2.92	2.35	2.89		
3.00	2.73	2.98	2.74		
3.07	2.75	2.50	2.59		
3.06	2.99	3.20			
3.09	2.97	2.08			
2.88	2.77	2.21			
2.66	2.78	2.91			
2.73	2.96	2.19			
3.20	3.10				
3.11	2.91				
3.12	2.91				
2.67	3.06				
2.88	3.12				
2.24	3.08				
2.47	2.90				
2.80	3.14				
2.23	2.75				
2.29	2.15				
2.65	3.09				
2.61	3.14				
2.80	2.83				
2.60	2.76				
2.79	2.87				

	2.97				
	3.06				
	2.58				

Spawning, ♀	Post-spawning, ♀

Panel L

>23–40, ♂	>23–40, ♀	41–60, ♂	41–60, ♀	60+, ♂	60+, ♀

10.02	9.32
9.94	9.19
9.80	9.16
9.98	9.55
9.03	10.60
9.92	10.01
9.40	9.53
9.60	9.78
9.67	10.52
9.46	9.36
10.01	8.96
9.44	9.58
10.04	9.38
9.80	9.77
9.76	10.16
9.18	9.41
9.87	10.16
9.47	9.12
10.03	9.07
9.75	8.90
9.41	9.22
9.57	9.33
9.62	8.97
9.78	9.71
9.78	9.09
9.68	9.39
10.05	10.16
9.73	9.08
9.79	9.03
9.70	9.83

9.22	10.02	9.59	10.20	9.96	9.37
9.60	9.94	9.39	8.97	10.09	9.42
8.96	9.80	9.98	9.53	9.99	9.44
10.07	9.98	9.29	9.87	8.92	9.22
9.11	9.03	9.13	10.19	9.51	9.33
8.81	9.92	10.08	9.58	9.49	8.97
9.19	9.40	9.03	9.38	8.76	9.71
8.90	9.60	8.78	9.77	9.34	9.09
9.15	9.67	10.19	10.16	9.72	9.39
9.63	9.46	8.85	9.41	9.11	10.16
9.25	8.94	9.39	10.16	9.86	9.08
8.90	10.39	9.76	9.12	10.13	9.03
8.94	9.74	9.05	9.07	9.15	9.83
8.94	9.20	9.49	8.90		9.44
8.90	8.87	9.19	9.41		9.22
9.33	10.54	9.02	9.57		
9.21	9.50	9.01	9.62		
9.80	9.49	9.91	9.78		
9.72	10.55	10.05	9.78		
9.28	9.63	9.37	9.68		
9.09	9.32	10.09	10.05		
9.96	9.19	9.98	9.73		
9.36	9.16	9.73	9.79		
10.08	9.55	9.88	9.70		
9.11	10.60	10.11	9.48		
9.50	10.01	9.62	9.33		
9.75	9.53	9.57			
9.54	9.78	9.40			
9.12	10.52	10.05			
9.85	9.36	10.00			
8.97	10.01	10.14			
9.66	9.44				
8.78	10.04				
9.05	9.80				
10.02	9.76				
10.03	9.18				
10.05	9.87				
10.13	9.47				
9.85	10.03				
9.75	9.75				
9.20	9.10				
9.41	9.12				
9.41	10.05				
9.25	10.11				
10.12	8.90				
8.75	8.96				
	9.42				

	9.37				
	9.42				

Spawning, ♀	Post-spawning, ♀
3.14	3.60

Panel O

>23–40, ♂	>23–40, ♀	41–60, ♂	41–60, ♀	60+, ♂	60+, ♀
2.65	3.14	2.75	3.07	3.12	2.63

2.89	2.79
2.91	2.95
3.41	3.13
3.39	2.59
3.16	2.74
2.91	2.60
3.41	2.95
3.29	2.56
2.80	2.99
3.37	3.03
3.38	3.18
2.97	3.03
3.17	3.31
2.61	2.71
2.97	3.14
3.25	3.20
3.19	2.66
2.74	3.01
2.93	2.66
2.89	3.24
3.40	2.86
2.73	3.11
3.16	2.90
3.36	3.39
3.19	3.18
2.80	2.85
3.34	2.79
3.03	2.83
3.26	2.66

2.61	2.89	2.92	3.12	2.69	2.71
3.14	2.91	3.18	3.10	2.61	2.86
3.23	3.41	3.32	2.75	3.33	3.24
3.34	3.39	2.60	3.08	3.09	2.86
2.60	3.16	2.90	3.18	3.12	3.11
2.96	2.91	2.93	3.03	3.36	2.90
2.91	3.41	3.09	3.31	3.36	3.39
2.89	3.29	2.63	2.71	2.80	3.18
3.23	2.80	2.75	3.14	2.75	2.85
2.98	3.35	3.32	3.20	2.66	2.79
2.75	3.26	2.66	2.66	2.87	2.83
3.36	2.58	2.73	3.01	3.32	2.66
2.79	3.35	2.90	2.66		2.86
3.00	3.50	3.17	2.89		3.24
2.64	2.79	3.21	3.40		
3.34	3.17	3.07	2.73		
2.74	2.47	2.95	3.16		
2.97	2.96	3.36	3.36		
3.04	3.40	2.83	3.19		
2.69	3.60	2.96	2.80		
3.06	2.79	3.24	3.34		
2.93	2.95	3.38	3.03		
2.78	3.13	3.20	3.26		
3.31	2.59	3.28	2.64		
2.89	2.74	2.63	2.79		
2.81	2.60	2.92			
3.31	2.95	2.77			
2.75	2.56	3.30			
3.03	2.99	3.13			
3.24	3.37	3.30			
3.25	3.38				
2.86	2.97				
3.27	3.17				
2.71	2.61				
2.99	2.97				
2.91	3.25				
2.99	3.19				
2.64	2.74				
3.39	2.93				
3.30	2.78				
2.63	3.30				
2.71	2.71				
2.68	2.92				
2.82	2.98				
3.03	3.03				
	2.63				
	2.63				

	2.71				

Spawning, ♀	Post-spawning, ♀
8.03	5.81
7.28	6.61

Panel R

>23–40, ♂	>23–40, ♀	41–60, ♂	41–60, ♀	60+, ♂	60+, ♀
7.77	8.03	5.90	7.05	7.87	6.47
6.44	7.28	5.31	6.93	7.13	5.06

7.97	6.96
7.85	5.40
8.22	7.04
7.17	6.82
7.45	6.21
7.84	7.90
7.71	6.95
6.39	5.86
6.63	7.51
6.75	5.18
5.05	7.90
7.97	5.41
5.09	6.92
7.29	7.15
7.43	8.00
7.18	6.31
7.69	6.34
7.21	6.00
7.44	6.01
7.75	8.15
7.37	7.69
8.09	7.88
8.00	8.38
7.19	8.28
7.45	6.13
7.73	6.77
8.28	6.15
7.44	7.02

7.28	7.97	6.27	5.70	7.49	6.38
8.08	7.85	5.87	7.78	7.86	6.01
7.67	8.22	5.89	6.36	8.43	8.15
5.93	7.17	7.09	5.18	7.61	7.69
5.67	7.45	5.59	7.90	7.74	7.88
6.32	7.84	6.39	5.41	6.49	8.38
5.62	7.71	6.56	6.92	7.87	8.28
5.56	6.39	6.81	7.15	8.04	6.13
5.21	7.11	6.03	8.00	7.10	6.77
6.19	6.28	7.87	6.31	7.77	6.15
7.10	6.88	5.37	6.34	5.21	7.02
7.83	6.31	6.92	6.00		6.38
7.58	6.71	7.00	7.44		6.01
6.17	7.28	8.30	7.75		
5.90	6.60	7.33	7.37		
8.15	6.67	7.92	8.09		
5.72	5.47	5.06	8.00		
5.49	5.18	6.20	7.19		
5.70	5.81	6.91	7.45		
8.15	6.61	7.63	7.73		
7.05	6.96	8.08	8.28		
7.44	5.40	7.91	7.44		
6.74	7.04	7.10	7.90		
7.81	6.82	7.78	7.73		
5.72	6.21	8.37			
5.71	7.90	7.30			
7.23	6.95	7.10			
7.69	5.86	6.35			
5.49	6.63	7.33			
8.05	6.75				
5.83	5.05				
8.29	7.97				
7.79	5.09				
6.99	7.29				
5.10	7.43				
6.05	7.18				
5.08	7.69				
7.03	7.21				
6.01	6.91				
6.55	5.02				
6.98	7.81				
7.18	8.27				
6.28	7.53				
5.42	7.51				
	8.13				
	6.47				
	5.06				

Spawning, ♀	Post-spawning, ♀
32.00	29.00
36.00	47.00
27.00	25.00

Panel U

>23–40, ♂	>23–40, ♀	41–60, ♂	41–60, ♀	60+, ♂	60+, ♀
25.00	32.00	43.00	41.00	43.00	32.00
39.00	36.00	23.00	31.00	24.00	30.00
26.00	27.00	47.00	38.00	29.00	45.00

31.00	40.00
36.00	32.00
36.00	35.00
33.00	43.00
35.00	46.00
31.00	40.00
28.00	25.00
29.00	39.00
38.00	35.00
29.00	42.00
24.00	43.00
33.00	34.00
38.00	39.00
27.00	35.00
20.00	42.00
26.00	37.00
36.00	32.00
23.00	33.00
29.00	32.00
37.00	33.00
37.00	36.00
36.00	36.00
26.00	30.00
31.00	35.00
21.00	31.00
32.00	37.00
26.00	21.00

25.00	31.00	37.00	43.00	33.00	33.00
45.00	36.00	36.00	42.00	29.00	32.00
29.00	36.00	43.00	35.00	43.00	33.00
23.00	33.00	43.00	42.00	26.00	36.00
28.00	35.00	40.00	43.00	35.00	36.00
20.00	31.00	29.00	34.00	39.00	30.00
46.00	28.00	27.00	39.00	44.00	35.00
36.00	43.00	26.00	35.00	40.00	31.00
33.00	21.00	27.00	42.00	21.00	37.00
43.00	22.00	24.00	37.00	44.00	21.00
39.00	36.00	38.00	32.00		45.00
22.00	26.00	30.00	23.00		33.00
40.00	25.00	37.00	29.00		
42.00	39.00	40.00	37.00		
25.00	39.00	45.00	37.00		
46.00	22.00	37.00	36.00		
21.00	22.00	43.00	26.00		
34.00	29.00	25.00	31.00		
46.00	47.00	39.00	21.00		
26.00	25.00	21.00	32.00		
44.00	40.00	31.00	26.00		
41.00	32.00	42.00	40.00		
21.00	35.00	21.00	45.00		
37.00	43.00	45.00			
32.00	46.00	44.00			
40.00	40.00	42.00			
26.00	25.00	38.00			
35.00	29.00	47.00			
42.00	38.00				
42.00	29.00				
32.00	24.00				
47.00	33.00				
37.00	38.00				
31.00	27.00				
22.00	20.00				
45.00	26.00				
37.00	36.00				
41.00	45.00				
32.00	30.00				
37.00	26.00				
39.00	24.00				
23.00	33.00				
20.00	39.00				
	41.00				
	32.00				
	30.00				

S7 Figure

Panel A

T. ilisha	A. sinensis	B. sanguinolentus	C. auratus	D. pastinaca	G. morhua	G. mediterraneus	G. maculatum
13.61	17.98	11.10	12.50	23.90		6.50	19.39

Panel B

T. ilisha	A. sinensis	B. sanguinolentus	C. auratus	D. pastinaca	G. morhua	G. mediterraneus	G. maculatum
8.95	12.65	8.00	8.50	15.50		4.10	15.50

Panel C

T. ilisha	A. sinensis	B. sanguinolentus	C. auratus	D. pastinaca	G. morhua	G. mediterraneus	G. maculatum
99.93	226.30	143.26	83.45	304.80	76.51	44.12	239.02

Panel D

T. ilisha	A. sinensis	B. sanguinolentus	C. auratus	D. pastinaca	G. morhua	G. mediterraneus	G. maculatum
5.52	7.66	5.30	5.50	10.60		2.60	7.96

Panel E

T. ilisha	A. sinensis	B. sanguinolentus	C. auratus	D. pastinaca	G. morhua	G. mediterraneus	G. maculatum
3.53	5.28	3.00	4.00	9.50		1.60	5.54

Panel F

T. ilisha	A. sinensis	B. sanguinolentus	C. auratus	D. pastinaca	G. morhua	G. mediterraneus	G. maculatum
16.08	32.88	13.53	17.72	79.33	17.33	3.46	35.78

L. piscatorius	N. melanostomus	N. erebi	O. keta	P. marinus	R. clavata	S. namaycush	S. niger
11.60	10.10	12.20		11.90	26.80	16.00	11.45

L. piscatorius	N. melanostomus	N. erebi	O. keta	P. marinus	R. clavata	S. namaycush	S. niger
9.20	7.60	8.00		11.90	14.00	11.30	8.62

L. piscatorius	N. melanostomus	N. erebi	O. keta	P. marinus	R. clavata	S. namaycush	S. niger
83.82	123.03	76.65	131.08	111.27	368.13	142.00	80.65

L. piscatorius	N. melanostomus	N. erebi	O. keta	P. marinus	R. clavata	S. namaycush	S. niger
5.60	5.10	4.80		4.00	6.70	6.50	5.23

L. piscatorius	N. melanostomus	N. erebi	O. keta	P. marinus	R. clavata	S. namaycush	S. niger
4.20	3.70	2.30		4.00	4.60	4.40	4.89

L. piscatorius	N. melanostomus	N. erebi	O. keta	P. marinus	R. clavata	S. namaycush	S. niger
18.86	15.21	9.90	35.16	12.57	25.07	23.33	20.11

S. plagiostomus	S. porcus	T. mediterraneus	X. gladius
10.32	11.20	7.80	11.70

S. plagiostomus	S. porcus	T. mediterraneus	X. gladius
6.43	7.70	5.00	8.30

S. plagiostomus	S. porcus	T. mediterraneus	X. gladius
70.32	140.28	64.34	76.27

S. plagiostomus	S. porcus	T. mediterraneus	X. gladius
4.23	4.10	3.50	4.00

S. plagiostomus	S. porcus	T. mediterraneus	X. gladius
3.52	2.40	1.70	3.40

S. plagiostomus	S. porcus	T. mediterraneus	X. gladius
11.79	8.30	5.31	10.75
